# Facile and selective *N*-alkylation of gentamicin antibiotics *via* chemoenzymatic synthesis†

**DOI:** 10.1039/d2gc03600b

**Published:** 2022-11-17

**Authors:** Gorjan Stojanovski, Helen C. Hailes, John M. Ward

**Affiliations:** Department of Biochemical Engineering, University College London London WC1E 6BT UK j.ward@ucl.ac.uk; Department of Chemistry, University College London 20 Gordon Street London WC1H 0AJ UK

## Abstract

The rise and spread of antimicrobial resistance has necessitated the development of novel antimicrobials which are effective against drug resistant pathogens. Aminoglycoside antibiotics (AGAs) remain one of our most effective classes of bactericidal drugs. However, they are challenging molecules to selectively modify by chemical synthesis, requiring the use of extensive protection and deprotection steps leading to long, atom- and step-inefficient synthetic routes. Biocatalytic and chemoenzymatic approaches for the generation of AGA derivatives are of interest as they allow access to more concise and sustainable synthetic routes to novel compounds. This work presents a two-step chemoenzymatic route to regioselectively modify the C-6′ position of AGAs. The approach uses a transaminase enzyme to generate an aldehyde on the C-6′ position in the absence of protecting groups, followed by reductive amination to introduce substituents selectively on this position. Seven candidate transaminases were tested for their ability to deaminate a panel of commercially available AGAs. The C-6′ transaminases could deaminate both pseudo di- and trisaccharide AGAs and tolerate the presence or absence of hydroxyl groups on the C-3′- and C-4′-positions. Additionally, sugar substituents on the C-6 hydroxyl were accepted but not on the C-5 hydroxyl. The most promising enzyme, GenB4, was then coupled with a reductive amination step to synthesise eleven novel 6′-gentamicin C1a analogues with conversions of 13–90%. Five of these compounds were active antimicrobials and four of these retained activity against an aminoglycoside-resistant *Escherichia coli*. This approach allows facile and step-efficient access to novel aminoglycoside compounds under mild reaction conditions and could potentially enable the development of greener, sustainable, and more cost-effective syntheses of novel AGAs.

## Abstract 2

A two-step chemoenzymatic route to enable regioselective modification of aminoglycoside antibiotic analogues was developed without the use of protecting groups. This approach allows rapid generation of novel antibiotic analogues.

## Graphical abstract

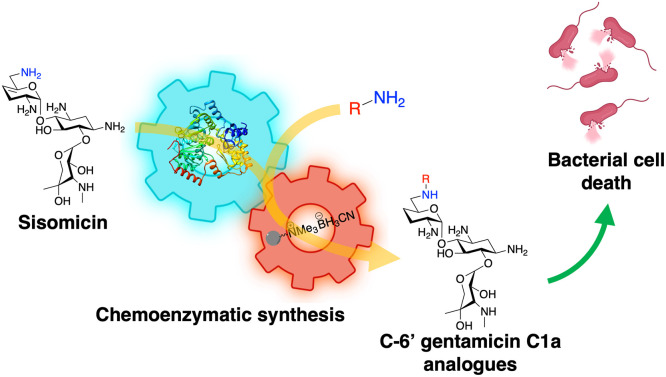

## Introduction

**Scheme 1:**
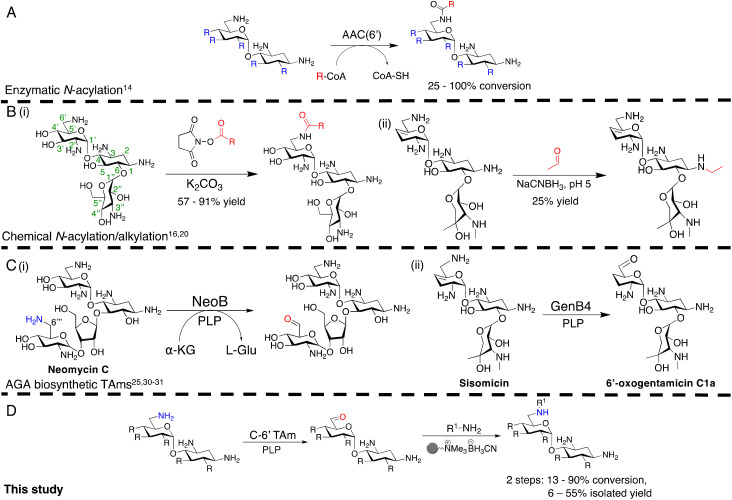
Overview of the prior methods for selective *N*-acylation/alkylation of AGAs, studies on AGA biosynthetic transaminases and our chemoenzymatic *N*-alkylation pathway. (A) Enzymatic acylation using AACs to generate novel acylated products, (B) Chemical approaches for regioselective *N*-acylation and alkylation, (C) Prior studies of reactions catalysed by AGA biosynthetic TAms, (D) The chemoenzymatic approach investigated in this study. R denotes variation at the position shown, and represents –H, –OH or –NH_2_, R on C-5 and C-6 denotes hydroxyl or glycosyl groups. The bead represents polymer-supported cyanoborohydride. Conversions were determined by HPLC. The positions of the aminoglycoside scaffold are labelled on the kanamycin B structure in the left of B(i).

Antimicrobial resistance (AMR) is a major global health crisis which has necessitated the development of novel antibiotics. Additionally, the poor financial incentives involved in antimicrobial research and development adds to the challenges of bringing novel antibiotics to market.^1^ Therefore, novel antibiotics need to both be effective against drug resistant microbes and made by affordable and sustainable methods to support longer term therapeutic success. Aminoglycoside antibiotics (AGAs) are highly potent bactericidal drugs and have been a cornerstone of clinical therapy since their discovery in 1944.^2^ However, their use has been discouraged clinically due to toxicity issues and the rise and spread of AMR. In addition, AGAs are challenging molecules to modify selectively as they are poly-hydroxylated and poly-aminated, often requiring multistep syntheses with protection and deprotection steps.^3–6^ However, mono-acylated/alkylated AGAs are of interest as they maintain potency against resistant pathogens.^7–9^ Therefore, efficient methods of selectively modifying AGAs are of considerable interest. In the past two decades, the characterisation of AGA biosynthetic gene clusters (BGCs) and pathways has enabled the investigation of enzymatic approaches to AGA synthesis. Sustainable enzymatic syntheses are of interest as they have mild reaction conditions and high regioselectivity which reduces side product formation and can ease often complex and costly product isolation. Selective N-1 acylation has been achieved using the butirosin biosynthetic enzymes, BtrG and BtrH.^10^ The Baasov group showed that by using an *N*-acetylcysteamine (SNAc) thioester of (*S*)-α-hydroxybutyric acid, a range of di- and trisaccharide AGAs could be selectively N-1 acylated in >90% yield.^11^ Similarly, aminoglycoside acetyltransferases (AACs), resistance enzymes which modify the N-6′ and N-3 amine groups have been shown to accept larger groups than their natural acetyl moiety and could acylate AGAs selectively in moderate yields (Scheme 1A).^12,13^ More recently, the AAC(6′) enzyme has been shown to acylate N-1 modified AGAs such as amikacin and isepamicin on the N-6′ position in quantitative yields.^14,15^ While effective, the use of AACs has limited scale-up potential due to the high cost of the coenzyme A co-factor used by these enzymes.

Regioselective chemical modification has also been explored. Direct acylation of AGA amino groups using *N*-hydroxysuccinimide esters can be regioselective for a specific amino group in the absence of protecting groups, with yields of 57–91% [Scheme 1B(i)].^3,16–19^

However, the reduction of the subsequent amides to allow a two-step *N*-alkylation, requires strong reducing agents or high hydrogenation pressures and temperatures, which can be dangerous, lead to toxic by-products and have high energy costs when employed on a large-scale. Regioselective ethylation has also been demonstrated using acetaldehyde and sodium cyanoborohydride, but with lower yields of 25% due to poor regioselectivity [Scheme 1B(ii)].^20^ Thus, mild, regioselective and cost-effective amine modification conditions are of considerable interest.

In this study, the aim was to investigate versatile and sustainable approaches toward AGA synthesis using transaminase (TAm) enzymes. From studies on the AGA biosynthetic pathways, TAms were shown to introduce several amine functionalities in the AGA scaffold.^21–28^ We were interested in enzymatic modification of the 6′-amino group due to its benefits for combatting AG resistance mechanisms.^9,29^ Prior research demonstrated that TAms which modify the C-6′ position (C-6′ TAms) naturally convert an aldehyde group to an amine and that these enzymes have considerable substrate scope.^21–24^ Interestingly, the C-6′-TAm, NeoB, from neomycin biosynthesis was also shown to deaminate the C-6′′′ position of neomycin C, generating an aldehyde in this position^21^ [Scheme 1C(i)]. More recently, another biosynthetic TAm, GenB4 from gentamicin biosynthesis, was shown to naturally convert sisomicin to 6′-oxogentamicin C1a in quantitative conversions by isomerisation of the C-4′/C-5′ double bond [Scheme 1C(ii)].^30,31^ These enzymatic reactions were of interest as they showed regioselective amine conversion to a more chemically versatile aldehyde group in a single step. The aldehyde could be utilised to allow regioselective modification of the C-6′ amine without the use of protecting groups. Additionally, the reactions could potentially provide access to analogues of single gentamicin congeners, which have lower toxicities^32^ and are difficult to generate directly from gentamicin obtained from fermentations. Here we explore the deamination reaction of the C-6′ position of AGAs using C-6′ TAms followed by sequential coupling in a reductive amination reaction to create a series of novel mono-alkylated gentamicin C1a AGAs (Scheme 1D). We then assessed the antimicrobial potency of these compounds.

## Results and discussion

### Bioinformatic analysis of C-6′ TAms

AGAs are naturally produced by actinomycetes, and hence are genetically encoded in aminoglycoside BGCs. Therefore, to find C-6′ TAms to test in the deamination reaction, the aminoglycoside BGCs of eleven known aminoglycoside producing organisms were searched (ESI Table S1†). The C-6′ TAm sequence from the firmicute, *Paenibacillus chitinolyticus* DSM 11030, was also included as this organism was found to have a complete butirosin-like gene cluster. In total, fifteen AGA biosynthetic TAm sequences were analysed. All enzymes were class III TAms, and analysis of amino acid sequence identity and homology suggested the presence of five groups with >70% sequence identity within a group and 20–40% sequence identity between groups (ESI Fig. S1A†). Within the groups there were several pairs of sequences with high sequence identity (ESI Fig. S1B†). It was expected that these high-identity homologues would have similar substrate scopes in the deamination reaction. Therefore, seven of these enzymes were selected for testing in the deamination reaction, these being KanB, ForB, PchB, NeoB, GenB1, GenB2 and GenB4 (ESI Fig. S1B†).

### Exploration of enzymatic deamination activity

Prior to this study, Huang and co-workers demonstrated that the C-6′ TAm, NeoB, could deaminate neomycin C on the 6′′′-position in the presence of α-ketoglutarate as an amine acceptor [Scheme 1C(i)].^21^ Therefore, for initial screening, their reaction conditions were used. The deamination activity of the selected enzymes was tested against a panel of eight commercially available aminoglycosides, all of which contained an amino group at the C-6′ position (Table 1). This panel contained a pseudodisaccharide [neamine (1a)], 4,6-disubstituted 2-deoxystreptamine (2-DOS) AGAs [kanamycin B (1b), kanamycin A (1c), tobramycin (1d), gentamicin C1a (1e) and sisomicin (1f)], a 4,5-disubstituted 2-DOS AGA [ribostamycin (1g)] and an N-1 acylated AGA [amikacin (1h)]. This panel was selected for the following reasons – the different substitution patterns present in the aminoglycosides allowed us to determine what effect different sugar substituents on the C-5 and C-6 positions had on the deamination reaction. Within the 4,6 disubstituted AGAs, the C-3′ and C-4′ positions are either unsubstituted or hydroxylated, allowing us to see if substituents close to the C-6′ position affected the reaction. Similarly, amikacin allowed us to see if substrates with bulky modifications on the N-1 position could be accepted by the C-6′ TAms.

**Table:** Relative product areas for deamination screening of aminoglycosides

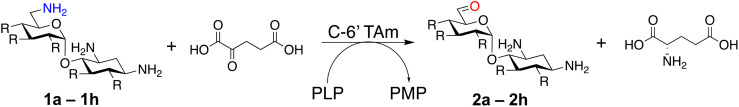								
Substrates	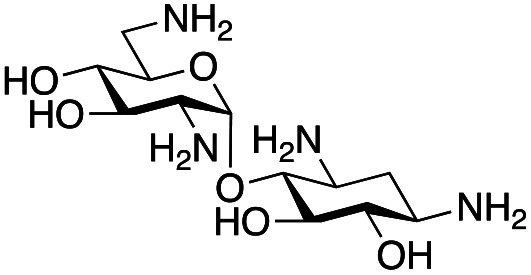	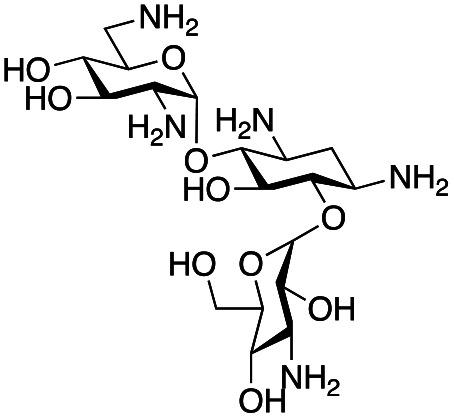	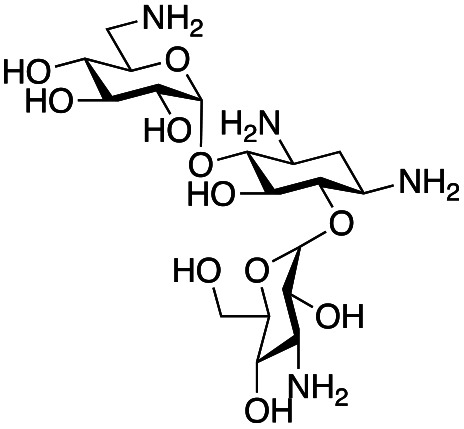	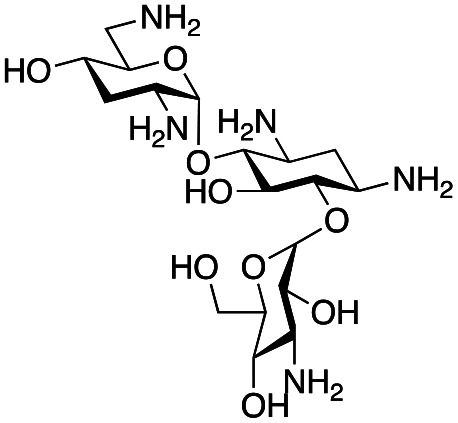	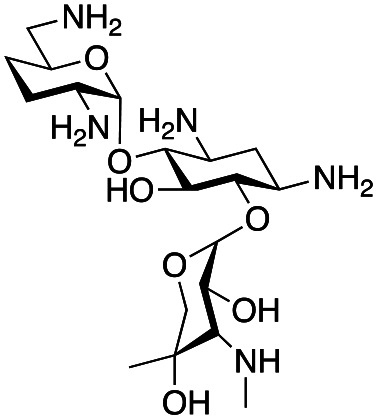	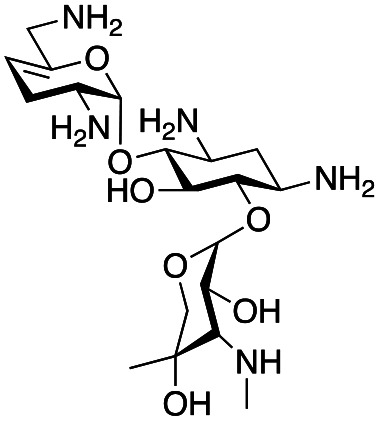	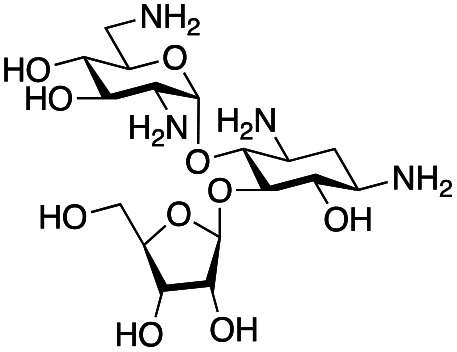	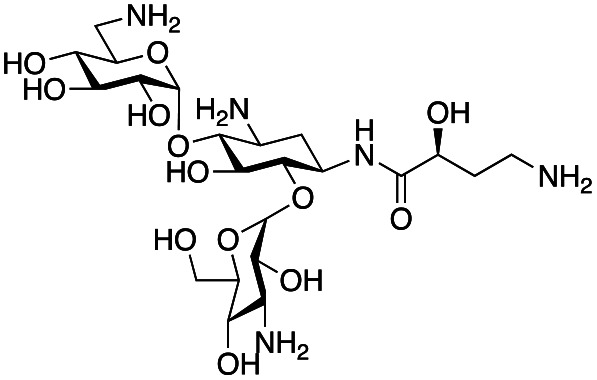
Enzyme	1a	1b	1c	1d	1e	1f	1g	1h
NeoB	19	32	1	3	2	24	—	—
GenB1	3	4	1	1	3	6	—	—
GenB2	1	1	—	5	3	5	—	—
GenB4	—	—	—	1	3	5 (94)^a^	—	—
KanB	—	—	—	—	1	—	—	—
PchB	1	10	1	1	1	2	—	—
ForB	1	—	—	1	3	24	—	—

a GenB4 displayed deamination and isomerisation activities on sisomicin (1f). 5% relative area is for the deamination product, 6′-oxosisomicin (2f), the number in brackets refers to the yield of the isomerisation product, 6′-oxogentamicin C1a (2e).

The selected enzymes were cloned, expressed, and purified (ESI Fig. S2†) and reactions with each of the AGAs were incubated for 24 hours and analysed by high-performance anion exchange chromatography with integrated pulsed amperometric detection (HPAE-IPAD). The aldehyde products formed were reported to be unstable^30,33^ and hence an external product standard was not available for quantification. Thus, deamination was monitored by a combination of relative product area and substrate depletion compared to an external standard (Table 1 and ESI Fig. S3†). Initial reactions showed that 1g and 1h were not accepted as substrates and 1c was only converted at trace levels (Table 1). Overall, the deamination conversions were low which is not unusual given that the aldehyde product is more reactive compared to the amine substrate. Despite this, some key observations were noted. Substrate 1g which has a 4,5-substitution pattern was not deaminated by any of the enzymes tested, while aminoglycoside substrates with a 4,6-substitution pattern (1b–1f) were accepted. Deamination of these substrates was also confirmed by liquid chromatography mass spectrometry (LC-MS) and tandem mass spectrometry (LC-MS/MS) analysis (ESI Fig. S13–S18†). The presence or absence of hydroxyl groups on the C-3′- and C-4′-positions did not appear to affect the deamination reaction, as neamine (1a), kanamycin B (1b) and sisomicin (1f) were all deaminated by NeoB in 19%, 32% and 24% conversions, despite having either no substituents or hydroxyl groups on the C-3′ and C-4′ positions (Table 1).

Intriguingly, the NeoB enzyme readily deaminated 1b with a 32% conversion but not 1c (trace conversion), which is interesting as the only difference between these two molecules is at the C-2′ position, with 1b having an amino group and 1c having a hydroxyl group. In fact, all substrates which were deaminated contained an amino group at the C-2′ position. Prior X-ray crystallographic analysis of NeoB in complex with PLP-neamine showed that the C-2′ amino group interacts by hydrogen bonding with Asp344.^34^ This same interaction was also observed between the C-2′′′ amino group and Asp344 for PLP-neomycin C in complex with NeoB.^34^ This suggested that a C-2′ amino group is important either for positioning the substrate or allowing deamination and transamination to occur. The importance of the C-2′ amino group for activity was also observed by Ban and co-workers for GenB1, who suggested that the amino group displaces a magnesium ion positioned in the negatively charged ‘hole’ in the GenB1 active site.^24^ The lack of conversion with substrate 1h may also be accounted for by the hydroxyl present on the C-2′ position in addition to the bulky N-1 substituent. Compounds 1d and 1e were also deaminated by the enzymes but in trace conversions (Table 1).

Substrate 1f was deaminated by NeoB, ForB and GenB4 in 25%, 24% and quantitative conversions, respectively. NeoB and ForB formed the expected 6′-oxosisomicin (2f) product as confirmed by LC-MS which showed a peak at *m*/*z* 447 consistent with the mass of 2f (ESI Fig. S17†). GenB4 formed some 2f with a 5% conversion, but a different product, 6′-oxogentamicin C1a (2e) was predominantly produced. This was confirmed both by the presence of a new peak at 12.2 minutes in the HPAE-IPAD chromatogram and by LC-MS/MS which showed a peak at *m*/*z* 467 consistent with the mass of the geminal diol of 2e (ESI Fig. S18†). This different reactivity of GenB4 was consistent with prior studies which showed that the enzyme isomerizes the C-4′/C-5′-double bond of 1f.^30,31^ ForB deaminated 1f in equal conversions to NeoB, despite being tested at almost half the enzyme concentration, however it had very little activity on all other substrates tested. Of the other enzymes screened, PchB showed around 10% conversions for 1b but did not show substantial conversion for any of the other substrates tested. GenB1, GenB2 and KanB were not active on any of the substrates tested.

Overall, the most promising enzymes for deamination of the aminoglycosides were NeoB and GenB4, as they could be purified in high protein yields, were stable under the assay conditions and had the highest product conversions for their respective substrates. Thus, NeoB and GenB4 were investigated further.

### Optimisation of GenB4 deamination reaction conditions

**Fig. 1:**
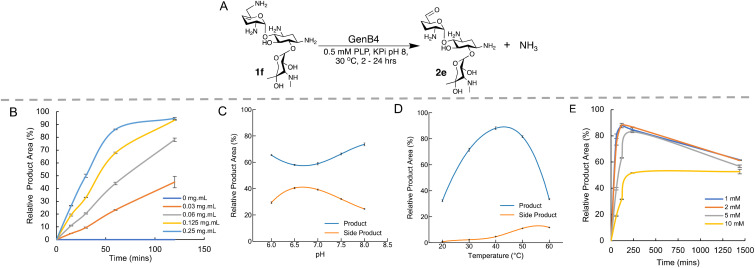
Optimisation of the GenB4 reaction conditions. (A) Scheme of the conditions used in the GenB4 assay. (B) Analysis of varying enzyme concentration over time, reactions were incubated for 2 hours. Means of triplicate reactions are shown, with error bars indicating standard deviations (<±5%). (C) Analysis of GenB4 reaction at different pHs. Reactions were incubated for 24 hours and side product formation is also shown. Reactions were performed in duplicate, means are shown and error bars are standard deviations (<±2%). (D) Analysis of GenB4 reaction at different incubation temperatures. Reactions were incubated for 24 hours and side product formation is also shown. Reactions were performed in duplicate, means are shown and error bars are standard deviations (<±1.5%). (E) Analysis of GenB4 reaction at different 1f concentrations. Reactions were performed in duplicate, means are shown and error bars are standard deviations (<±2%). The decrease in product yield observed at 1440 min reaction time is due to side-product formation.

GenB4 and NeoB had the highest activities in the preliminary screen and hence deamination conditions for each enzyme were optimised. GenB4 converted 1f to 2e in near quantitative yield. Additionally, Li and co-workers demonstrated that GenB4 does not require an amine acceptor for the C-4′/C-5′ double bond isomerisation^31^ (Fig. 1A), which is favourable as it reduces the number of components present in the reaction and could potentially ease subsequent product isolation. Therefore, the reaction conditions of the GenB4 mediated isomerisation of sisomicin (1f) were explored.

Firstly, the reaction was examined in terms of enzyme concentration. Prior studies on the GenB4 enzyme used substrate concentrations of 2 mM and 0.75–1 mg mL^−1^ of enzyme,^30,31^ which is quite high when viewed from a sustainability perspective. In the preliminary screen described, quantitative conversion of 1f was observed in 24 hours using 0.2 mg mL^−1^ of GenB4. Therefore, it was important to explore this further. 2 mM of 1f was incubated under the conditions described in the presence of 0.03–0.25 mg mL^−1^ GenB4 and time-point samples were analysed by HPAE-IPAD (Fig. 1B). This showed that complete conversion of 1f was achieved after 2 hours with 0.125–0.25 mg mL^−1^ of GenB4, which are 3–4-fold lower concentrations than those used in reported assays.^30,31^ Product conversions were similar in the presence and absence of α-ketoglutarate, which agreed with results reported by other groups^30,31^ (ESI Fig. S4A†). Therefore, for all other optimisation experiments of GenB4, α-ketoglutarate was not added. The next factors examined were reaction pH, temperature, and substrate concentration. For these experiments the conditions were separately tested by varying the pH from 6.0–8.0, the temperature from 20 °C–60 °C and the sisomicin concentration from 1–10 mM, respectively (Fig. 1C–E). This showed that overall, reaction pH mildly affected conversions and the greatest conversions were observed at pH 8.0. GenB4 had a broad temperature tolerance, with an optimum observed at 40 °C and the enzyme was still highly active at 50 °C. Finally, the substrate concentration assay suggested that maximal conversion was observed up to a substrate concentration of 5 mM, while 10 mM of substrate led to an approximate 50% decrease in conversion.

Interestingly in these experiments, a second peak was observed at 3.2 min in the HPAE-IPAD chromatogram, that appeared to increase linearly over time (ESI Fig. S4B†). The formation of this peak was more prevalent at pH 7.0, higher reaction temperatures and increased as the concentration of 1f increased (Fig. 1C and D, ESI Fig. S4B†). While the identity of this product was not known, it was speculated that it could be a degradation product of 6′-oxogentamicin, as its formation followed generation of the aldehyde and was greater at higher temperatures. However, it was not known if its formation was dependent on GenB4. Substrate 1f was incubated with GenB4 and PLP for 1 hour at 45 °C, and after this the GenB4 enzyme was either denatured by incubation at 95 °C for 10 minutes or maintained at 45 °C. After this both reactions were incubated for a further 24 hours. Samples were taken at 0, 1 hour and 24 hours of reaction and analysed by HPAE-IPAD (ESI Fig. S5†). This showed that in the reactions incubated at 95 °C, the peak at 3.2 min was predominant and very little of the aldehyde was present (ESI Fig. S5†). After 24 hours, both the reactions in which the GenB4 enzyme was denatured and where the GenB4 enzyme was intact looked similar. This suggested that formation of the second unknown product was not dependent on GenB4 and was heavily temperature dependent.

Analysis of the heat-treated GenB4 reaction by LC-MS showed the presence of two peaks with a mass of *m*/*z* 431 (one at 7 min and another at 11 min). This mass was consistent with the mass of an intramolecular imine or a doubly charged self-dimer of 2e and LC-MS/MS analysis showed fragments consistent with some of these products (ESI Fig. S6†). This result was further verified by reduction of the imine with polymer supported cyanoborohydride which generated products with a mass of *m*/*z* 433 consistent with the mass of an intramolecular amine or a doubly charged reduced self-dimer of 2e (ESI Fig. S6†). Overall, this suggested that the 2e aldehyde was quite reactive in solution, and for downstream experiments, high reaction temperatures, neutral pHs and prolonged incubation times were avoided. Additionally, once the aldehyde was prepared, it was used directly in the next step to minimise side product formation.

In contrast to GenB4, the NeoB enzyme utilises a classic ping-pong bi-bi reaction mechanism to generate its aldehyde product.^21^ Thus, the reaction conditions were tested with varying enzyme concentrations and using l-glutamate dehydrogenase to convert the l-glutamate by-product back to the amine acceptor α-ketoglutarate.^35^ However, neither approach led to substantial improvements in product conversions (data not shown). Therefore, only the GenB4 enzyme was tested in chemoenzymatic cascades.

### Optimization of coupled reductive amination conditions

The generation of an aldehyde at the C-6′ position allows a diverse range of reactions to be performed, such as reduction, aldol condensation and organocatalytic click chemistry involving azide-aldehyde [3 + 2] cycloadditions.^36^ We decided to couple the GenB4 reaction with a reductive amination reaction, as it allowed a diverse range of alkylated amines to be introduced selectively at this position, which can be challenging to achieve without prior protection of competing amine groups. In the presence of cyanoborohydride, two side reactions can occur, direct reduction of the aldehyde to an alcohol and for amine containing aldehydes, irreversible reduction of intramolecular imines and self-dimers (ESI Fig. S7†). Therefore, optimisation of the reductive amination conditions was initially investigated and benzylamine (3f) and phenethylamine (3g) were used as model substrates.

Firstly, the reaction pH for the reductive amination was optimized as low pHs promoted formation of the alcohol by-product and neutral pHs led to more intramolecular amine/self-dimer formation. For this, 2e was formed using GenB4, which was then reacted with 3f/3g and cyanoborohydride. HPLC analysis of the 3f reactions indicated that the highest product conversions were obtained at pH 5.0 (ESI Fig. S8A†). This was also observed when using 3g, and LC-MS analysis suggested that product conversion dropped at pH 4.0 due to greater direct aldehyde reduction (ESI Fig. S8B†). At higher pHs of 6.0–7.0, no difference in product formation was observed, while the aldehyde concentration decreased, possibly indicating decreased aldehyde stability at these pHs (ESI Fig. S8B†). Formation of the desired product also increased as the number of equivalents of 3g increased (ESI Fig. S8C†).

Reductive aminations are often performed in organic solvents as water present in the reaction can both hydrolyse the imine intermediate and promote alcohol by-product formation. Methanol had previously been used as a solvent for reductive amination when applied to the synthesis of 6′-analogues of neomycin B.^37^ Methanol was also of interest as it denatures GenB4 prior to the chemical reaction and is a relatively environmentally benign organic solvent.^38,39^ However, at high methanol concentrations the solubility of the aldehyde substrate may be limiting. A preliminary screen of different concentrations of methanol for reductive amination was conducted. After the GenB4 reaction, samples were fractionated, the volume was reduced by rotary evaporation and the samples were diluted with methanol to final concentrations of 50–90% of methanol. A reductive amination reaction with 3g was then performed and analysed by LC-MS (ESI Fig. S9A†). This showed that comparable product conversions were obtained between 50–60% of methanol, but this decreased at higher methanol concentrations (ESI Fig. S9A†). To test if substrate solubility was the issue, the solubility of gentamicin C1a was examined. This showed that from 10–40% methanol content, gentamicin C1a was readily soluble, but decreased rapidly between 40–50% methanol and above 60% methanol, gentamicin C1a was no longer soluble (ESI Fig. S9B†). To check this further, 2e solubility was directly assessed (ESI Fig. S9C†). This suggested that most of the aldehyde was still present in a 50% methanol : water solution. Hence as solubility of the substrate was considered an issue above 60% methanol, 50% methanol was considered ideal.

### Synthesis of gentamicin C1a analogues *via* the GenB4 and reductive amination route

With the reductive amination parameters optimised, the conditions were tested on a panel of 16 other amines in addition to 3f and 3g (Table 2). This consisted of ten aromatic amines (3a–3j) and eight non-aromatic amines (3k–3r), representative of simple aromatic and aliphatic amines and modifications of aminoglycosides which had been previously shown to maintain antimicrobial potency.^29,37,40–45^

Preliminary amine reactivities in the coupled GenB4 + reductive amination synthetic route

**Table:** 

Compound	3a	3b	3c	3d	3e	3f	3g	3h	3i
Amine	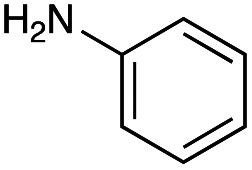	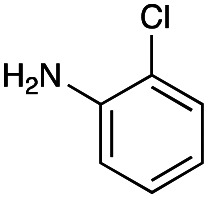	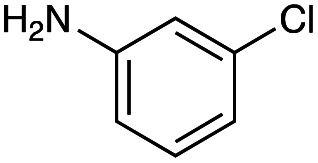	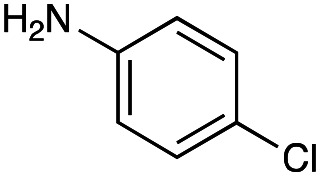	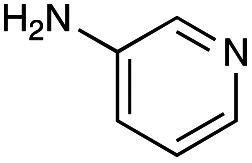	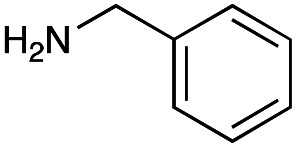	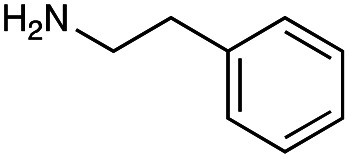	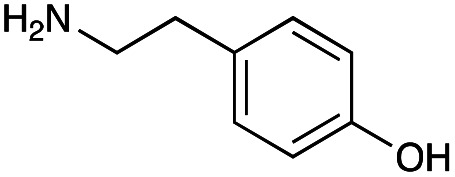	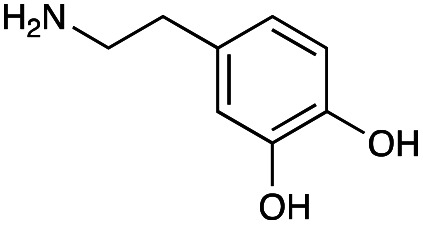
Reactivity^a^	+++	+	+++	+++	++	++	+	+	Trace

**Table:** 

Compound	3j	3k	3l	3m	3n	3o	3p	3q	3r
Amine	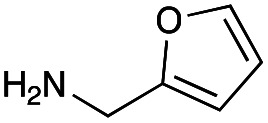	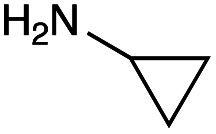	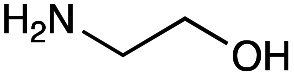	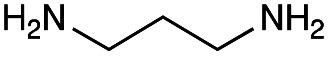	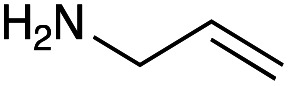	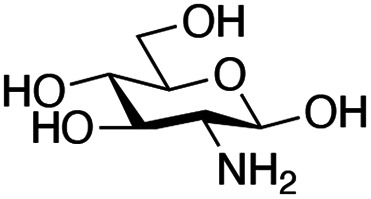	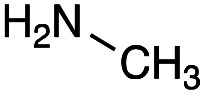	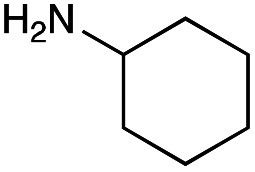	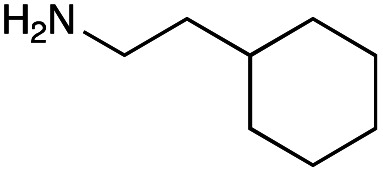
Reactivity^a^	++	+++	+	++	+	++	Trace	−	−

a Initial comparison of the different amine reactivities. Relative peak areas were obtained from the selective ion monitoring chromatograms of the geminal diol of 2e (substrate, *m*/*z* 467), desired reduced amine product (product, mass varied depending on product; 4a = *m*/*z* 526, 4b = *m*/*z* 560, 4c = *m*/*z* 560, 4d = *m*/*z* 560, 4e = *m*/*z* 527, 4f = *m*/*z* 540, 4g = *m*/*z* 554, 4h = *m*/*z* 570, 4i = *m*/*z* 586, 4j = *m*/*z* 530, 4k = *m*/*z* 490, 4l = *m*/*z* 494, 4m = *m*/*z* 507, 4n = *m*/*z* 490, 4o = *m*/*z* 612, 4p = *m*/*z* 464, 4q = *m*/*z* 532, 4r = *m*/*z* 560), 6′-hydroxygentamicin C1a (alcohol, *m*/*z* 451) and intramolecular amine by-products (side products, *m*/*z* 433). The areas obtained were summed to obtain a total area and each area was then divided by this total to obtain the relative peak area as a percentage. Reactivity grades are as follows: “+++” = >75% product peak area, “++” = 40–75% product peak area, “+” = 20–40% product peak area, “trace” = 10–20% product peak area, “−” = <10% product peak area.

Prior to scaling up the reaction, the reductive amination was tested in a preliminary screen with the 18 amines to determine amine reactivity. As such, 10 equivalents of each amine (12.5 mM) were used and the reductive amination was conducted at room temperature for 18 hours. The results were analysed by LC-MS analysis (Table 2, for MS spectra and characterisation of all compounds see ESI†).

**Fig. 2:**
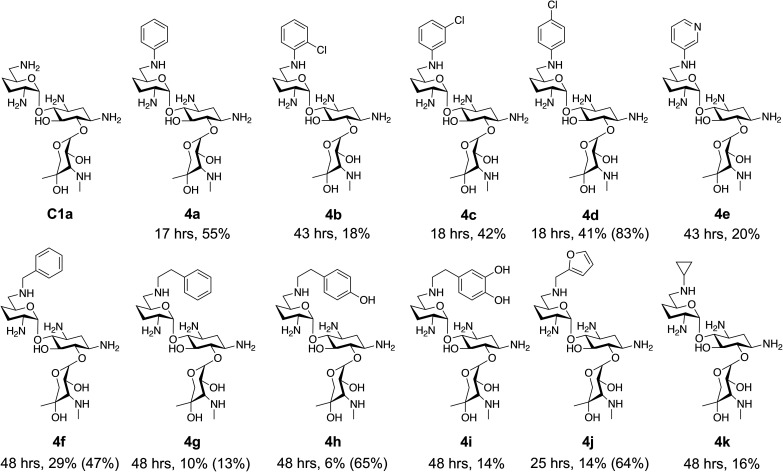
The 6′-gentamicin C1a derivatives tested for antimicrobial activity. Time in hours refers to the time incubated in the presence of cyanoborohydride. Percentage values are isolated yields. Values in brackets are the conversion yields determined by HPLC (standard deviation = <±7%). Compounds 4a–4f were purified directly by semi-preparative HPLC following reaction work-up. Compounds 4g–4j were purified by tetraphenylborate precipitation (see Experimental) followed by semi-preparative HPLC.

This showed that aniline and its *meta*- and *para*- analogues (3a, 3c–3d) had high conversions with up to 99% product peak area and complete consumption of the aldehyde substrate. Substitution on the *ortho*-position (3b) reduced the reactivity. The next best reactivities were observed with 3e, 3k, 3m and amines with aromatic aminomethyl groups (3f and 3j). Substrates 3g–3i reacted with reduced conversions due to reduced stability of the imine which is not conjugated in these substrates (Table 2). Non-aromatic amines with alkyl chains (3l and 3n) also had lower reactivities. 1,3-Diaminopropane (3m) had better reactivity than other alkylamines due to the presence of two amino groups in the molecule which can react with 2e. d-Glucosamine (3o) had a surprisingly good conversion. Methylamine (3p), cyclohexylamine (3q) and 2-cyclohexylethylamine (3r) had low conversions. Overall, for the poorly reacting amines, higher amine equivalents were needed to drive the reaction toward the desired aminated product along with longer reaction times. Reductive amination with amines 3p–3r were not pursued further due to low reactivities. Thus, 15 of the tested amines were taken forward for larger scale reactions and isolation of products 4a–4o was explored (Fig. 2). After reaction, the products were isolated by a modified version of tetraphenylborate precipitation,^46^ followed by semi-preparative HPLC. However, isolated yields were low (6–17%) using this procedure. Direct isolation of the product by semi-preparative HPLC for the aniline (3a) and benzylamine (3f) reactions, improved the product yields to 50% and 29%, respectively and this approach was used for the 3b–3e reactions. For products 4l–4o, while reactions were successful on a larger scale, the products were not obtained in sufficient purity for characterisation purposes by NMR spectroscopy.

To determine reaction conversions, compounds 3d, 3f–3h and 3j were used in a reductive amination reaction. After 41 hours of reaction, most substrates tested had reaction conversions of around 50–80% (Fig. 2). However, 3g had low reaction conversions. This indicated that for most compounds issues related to compound purification accounted for the low isolated product yields for the upscaled reactions. With the novel products isolated, their antimicrobial activity was then assessed.

### Antimicrobial activity of novel gentamicin C1a analogues

The GenB4 reaction was effectively coupled with a reductive amination to provide access to a series of eleven C-6′ gentamicin C1a analogues (Fig. 2). It was of interest to determine the antimicrobial activity of the synthesised compounds.

They were initially tested on a panel of six wild-type bacteria representative of Gram-negative and Gram-positive bacteria and major microbial pathogens such *Staphylococcus aureus*, *Klebsiella pneumoniae* and *Salmonella typhimurium* (Table 3). This testing showed some interesting observations. Firstly, the aryl derivatives (compounds 4a–4e) were inactive against all organisms in the tested concentration range (Table 3).

**Table:** MIC data from antimicrobial screening of wild-type bacteria

Organism	MIC (μM)											
	C1a	4a	4b	4c	4d	4e	4f	4g	4h	4i	4j	4k
*E. coli*	16–32	>28	>27	>27	>27	>28	7–14	14–27	27–54	> 26	7–14	4–8
*S. aureus*	16–32	>113	>108	>108	>108	>113	7–14	55–109	54–107	26–52	14–28	8–15
*P. aeruginosa*	16–32	>113	>108	>108	>108	>113	28–56	>109	>107	>105	28–56	15–30
*S. typhimurium*	16–32	>28	>27	>27	>27	>28	7–14	14–27	>27	>26	7–14	8
*B. subtilis*	1–2	>7	>7	>7	>7	>7	0.45–1	0.45–1	0.8–1.7	>7	0.45–1	0.23–0.47
*K. pneumoniae*	1–2	>7	>7	>7	>7	>7	0.45–1	0.45–1	0.8–1.7	>7	0.45–1	1–2
*E. coli* + pQR1865	128–254	—	—	—	—	—	7–14	27–55	27–53	—	7–14	8–15
*E. coli* + pSa	128–254	—	—	—	—	—	28–56	55–109	107–214	—	28–56	60–120
*E. coli* + R26	128–254	—	—	—	—	—	28–56	27–55	107–214	—	56–113	60–120

However, once the alkyl chain next to the 6′-amino group was increased by one or two carbon atoms, highly potent derivatives were observed. The benzyl- and furfuryl-derivatives (compounds 4f and 4j) had MICs of 0.45–56 μM against all tested organisms, similar to gentamicin C1a (Table 3). The phenethyl derivative (4g) had similar MICs to gentamicin C1a against *B. subtilis* and *K. pneumoniae* but was 1 to 4-fold less effective than gentamicin C1a against the other organisms. The addition of a hydroxy group in the *para*-position of the aromatic ring did not significantly affect antimicrobial potency (compare compounds 4g and 4h in Table 3). However, the addition of a further hydroxy group in the *meta*-position (4i) negatively affected antimicrobial potency.

The cyclopropyl derivative (4k) was 2 to 4-fold more potent compared to gentamicin C1a with MICs of 0.23–15 μM. It also had similar potency to gentamicin C1a against *P. aeruginosa*, while all other compounds tested were less effective against *P. aeruginosa* (Table 3). While the phenyl derivatives were inactive, some growth inhibition was seen at 27 μM with the 4-chlorophenyl derivative (4d) against *E. coli* ATCC 25922, which may suggest *para*-substituents on other scaffolds could have equivalent or enhanced antimicrobial potency. While the furfuryl- and cyclopropyl-derivatives (4j and 4k) were highly potent in this screen, there is a caveat to this as they were not as pure as the other compounds tested, with estimated purities of 85% and 80% respectively. The impurities present may themselves possess antibiotic activity or have synergistic activity with 4j or 4k, but the preliminary antibiotic activity testing described here highlights that these modifications may be beneficial for maintaining antimicrobial potency.

Overall, from the testing on wild-type organisms, five compounds were found to have antimicrobial activities similar to gentamicin C1a, these being compounds 4f–4h, 4j and 4k (Fig. 2). Subsequently, these compounds were tested against drug resistant bacteria to determine if they maintained their antimicrobial activity.

### Antimicrobial activity of compounds against drug-resistant organisms

When testing antimicrobial compounds against drug-resistant bacteria, it is often common to screen against strain isolates which express single aminoglycoside resistance genes or combinations of these. However in a clinical setting, it is not possible to select which antibiotic resistances are present or absent in a drug resistant infection.

Additionally, many of these genes reside on large multi-drug resistance plasmids which render an organism resistant to a plethora of antibiotic classes. To test for activity against drug resistant organisms, two multi-drug resistance plasmids were selected: pSa (a 37 kb IncW plasmid which has an *aac(6′)-Ib* gene) and R26 (an 80 kb IncP-1α derivative which has an *aac(3)-Ia* gene).^47,48^ The *aac(6′)-Ib* and *aac(3)* genes both confer resistance to gentamicin C1a.^49^ Plasmid pSa also has an *ant(3′′)-Ia* gene which confers resistance to streptomycin and spectinomycin but not to 2-DOS AGAs.^49,50^

Plasmid R26 contains an *ant(3′′)-Ia* gene and also an *aph(3′)-I* which phosphorylates the hydroxyl on the C-3′ position in 2-DOS AGAs. However, gentamicin and its derivatives do not have a hydroxyl group on the C-3′ position and thus are not affected by this gene.^49^ Additionally, a pUC19 plasmid containing a *Bgl*II fragment of pSa which has just the *aac(6′)-Ib* gene and *ant(3′′)-I* genes (pQR1865) was used to see if the compounds were effective against an *aac(6′)-Ib* gene in a simpler genetic context.

For an isogenic background, the plasmids were transformed into competent cells of *E. coli* ATCC 25922 and kanamycin resistant colonies were selected. From these colonies, plasmids were isolated and digested, and agarose gel electrophoresis suggested that pQR1865 was successfully transformed (ESI Fig. S10†). However, transformation of pSa and R26 was not clear by gel electrophoresis due to the presence of other native plasmids in *E. coli* ATCC 25922 (see ESI Discussion†). Thus, the antibiotic resistance profiles of the strains were also tested. The *E. coli* host strain was sensitive to gentamicin and kanamycin, while the strains containing the plasmids were resistant (ESI Fig. S11†). The host strain was mildly resistant to streptomycin, while this was enhanced in the strains carrying the resistance plasmid (ESI Fig. S11†). This suggested that the plasmids were successfully transformed. Thus, the five active compounds from the wild type MIC testing were then screened against these three gentamicin resistant strains (Table 3 and ESI Fig. S12†).

The first observation was that all resistance plasmid transformed strains were resistant to gentamicin C1a with MIC values of 128–254 μM, which is 8-fold higher than its potency against wild type *E. coli* (Table 3). In contrast, all C-6′ derivatives tested were not inhibited by the *aac(6′)-Ib* gene, as they had similar antimicrobial activity against *E. coli* carrying pQR1865 as they did against the parent *E. coli* (Table 3 and ESI Fig. S12†). For the strains carrying pSa and R26, a decrease in activity was observed, with compounds 4f, 4g, 4h and 4j seeing a 4-fold increase in MIC and compound 4k seeing a 16-fold increase (Table 3). The benzyl derivative (4f) appeared to be the most potent compound against R26 and pSa carrying *E. coli* with an MIC of 28–56 μM and the largest zones of clearing when tested on agar plates (Table 3 and ESI Fig. S12†). Overall, this showed that the compounds were effective against an organism expressing an *aac(6′)-Ib* gene when the gene is present in a simpler genetic background. While these compounds were more potent against multi-drug resistant *E. coli* strains compared to gentamicin C1a, their activity was still inhibited when the aminoglycoside resistance genes were present in multi-drug resistance plasmids. This may suggest that the action of other genes in the plasmids may inhibit the compounds' activity.

## Conclusions

In summary, the GenB4 isomerisation of sisomicin (1f) was utilised to selectively generate an aldehyde on the C-6′ position. This aldehyde was then converted *via* a reductive amination to generate a series of novel *N*-monoalkylated gentamicin C1a analogues in relatively high conversions and under mild reaction conditions. Five of these compounds (4f–4h, 4j and 4k) were effective as antimicrobials with similar potency to gentamicin C1a, and four of these compounds (4f, 4g, 4j and 4k) were effective against a drug-resistant *E. coli* strain. The two-step sequential synthesis shown here is advantageous compared to prior chemical techniques as it is selective, has mild reaction conditions and greater potential scalability due to more cost-effective co-factor requirements. It also provides access to gentamicin C1a as a core scaffold, which is of interest due to its lower ototoxicity compared to other gentamicin congeners.^32^ Additionally, while aldehyde reactivity is a potential issue, we demonstrate that reactions can still proceed in relatively high conversions under the reaction conditions used. Finally, generation of the aldehyde creates a versatile functional group allowing a wide range of reactions to be performed. Thus, novel AGAs could be rapidly generated and explored. Such selective and efficient synthetic routes to novel antibiotics are of increasing importance for the sustainable development of effective clinical therapeutics to tackle AMR.

## Author contributions

G. S. investigated the enzymatic reactions and developed the methodologies. All authors contributed to the conceptualisation of the project, and supervision was provided by H. C. H. and J. M. W. The original draft of the manuscript was written by G. S. The manuscript has been reviewed and edited by all contributing authors.

## Conflicts of interest

There are no conflicts to declare.

## Supplementary Material

GC-024-D2GC03600B-s001
