# Supplementary material for: Facile and selective N-alkylation of gentamicin antibiotics via chemoenzymatic synthesis
Source: Green Chem. 2022 Nov 17;24(24):9542–51. doi: 10.1039/d2gc03600b (PMC9744104; doi:10.1039/d2gc03600b)
Supplement: GC-024-D2GC03600B-s001 [file GC-024-D2GC03600B-s001.pdf]

Electronic Supplementary Material (ESI) for Green Chemistry.
This journal is © The Royal Society of Chemistry 2022

Supporting Information

Facile and selective *N*-alkylation of gentamicin antibiotics via chemoenzymatic synthesis

Gorjan Stojanovski[a,b], Helen C. Hailes[b] and John M. Ward*[,a]

Table of Contents

1. Experimental 2
 1.1 General molecular biology methods 2
 1.2 General chemistry methods 2
 1.3 Genome mining, multiple-sequence alignment and phylogenetic analysis 2
 1.4 Cloning, expression and purification of C-6' TAms 2
 1.5 AGA C-6' TAm deamination reactions 3
 1.6 GenB4 isomerisation assay and reaction optimisation 4
 1.7 Analysis of GenB4 reaction side product formation 4
 1.8 Reductive amination pH assay 4
 1.9 Reductive amination methanol content assay 5
 1.10 Substrate solubility tests 5
 1.11 Reductive amination conversion assay 6
 1.12 Antimicrobial activity screening of 6'-gentamicin C1a derivatives 6
 1.13 Aminoglycoside purification methods 7
 1.14 Analytical Procedures 8
 1.15 General synthesis of 6'-gentamicin C1a derivatives 9
2. Supplementary Discussion 16
3. Supplementary Tables 17
4. Supplementary Figures 20
5. LC-MS analysis of 6'-oxoaminoglycosides 30
6. NMR and HRMS Spectral Data 41
7. LC-MS analysis of reductive amination products 74
8. References 90

1. Experimental

1.1 General molecular biology methods

Molecular biology reagents were obtained from New England Biolabs (NEB) or Thermo Scientific. Spin column purification and gel extraction of DNA fragments was performed using a Monarch PCR and DNA Clean-up kit and DNA Gel Extraction kit using the manufacturer's instructions, respectively. Mini-preps of plasmid DNA were performed using a QIAprep Spin Miniprep Kit (QIAGEN) using the manufacturer's instructions. Synthesis of oligonucleotides for PCR, synthetic genes and Sanger DNA sequencing were all performed by Eurofins Genomics.

1.2 General chemistry methods

All compounds were purchased from Sigma-Aldrich, Alfa Aesar or Acros Organics and used without further purification. Freeze drying of samples was performed using a SP VirTis Benchtop Pro with Omnitronics (SP Scientific) with the freeze drier connected to a vacuum pump (Edwards). Centrifugal evaporation was performed using a Jouan RC1022 centrifugal evaporator connected to a Heto cooling trap and vacuum pump.

^{1}H and ^{13}C nuclear magnetic resonance (NMR) spectra were recorded at 600 MHz and 150 MHz, respectively on a Bruker Avance III 600 instrument equipped with a helium-cooled cryoprobe. NMR spectra were recorded in deuterium oxide [reference peak: ^{1}H NMR: HOD (4.79 ppm), ^{13}C: TFA salt peaks (quartets at 163.7 and 117.0 ppm)]. For ^{1}H NMR spectra, chemical shifts are quoted to the nearest 0.01 ppm, with signal splittings reported as: broad (br), singlet (s), doublet (d), triplet (t), quartet (q), multiplet (m) or combinations thereof. For ^{13}C NMR spectra, chemical shifts are reported to the nearest 0.1 ppm. All NMR data were obtained in the UCL Chemistry NMR Facility.

High-resolution ASAP-HESI mass spectra were acquired using an ASAP probe integrated into a HESI ion source connected to a Q-Exactive Plus mass spectrometer. All mass spectral data were obtained in the UCL Chemistry Mass Spectrometry Facility.

1.3 Genome mining, multiple-sequence alignment and phylogenetic analysis

Protein sequences for literature reported C-6' TAms and sequence homologues were searched for in the sequence data of 12 aminoglycoside producing organisms with published BGCs (Table S1), to give 15 TAm sequences. These sequences were passed through the ClustalOmega web service to generate a percentage identity matrix, which was visualised in Python 3.0 using the *seaborn* package. They were subsequently aligned using the MAFFT program with default settings in Jalview 2.10.5, and a phylogenetic tree was created using the maximum-likelihood method and 500 bootstrap repetitions in MEGA X. FigTree v1.4.4 was used to visualise and annotate the phylogenetic tree.

1.4 Cloning, expression and purification of C-6' TAms

1.4.1 Cloning of C-6' TAm homologues

NeoB, KanB, ForB, GenB1, GenB2 and GenB4 genes were codon-optimised and prepared synthetically by Eurofins Genomics. The capture vectors from Eurofins containing the NeoB,

KanB, ForB, GenB1, GenB2 and GenB4 genes were digested with *Nde*I and *Xho*I, DNA fragments were purified by gel extraction and ligated into pET28a(+) digested with the same enzymes. These constructs had a 6x-His tag at the *N*-terminus followed by a thrombin cleavage site. The gene encoding PchB was amplified from *Paenibacillus chitinolyticus* DSM 11030 genomic DNA by PCR using the following conditions: 1x Phusion High-Fidelity PCR master mix with HF buffer (2x stock from NEB), 1 µM forward and reverse primers, 1 µL template DNA, 10% DMSO, up to 50 µL with sterile H_2O [Thermocycling conditions - 98 °C/5 mins, 30 cycles of (98 °C/10 secs, 72 °C /30 secs, 72 °C/45 secs), 72 °C/7 mins]. Primers are described in Table S2. PCR products were digested with *Sap*I and cloned into pET29a(+)-*sacB*[1] to give plasmid pQR2763. This construct contained a C-terminal 6x-His tag following the last amino acid. All plasmids were verified by restriction digestion and DNA sequencing.

1.4.2 Expression of C-6' TAms

The plasmids were transformed into *E. coli* BL21(DE3) and a single colony was inoculated into 10 mL Terrific Broth (TB, Merck) containing kanamycin (50 µg/mL) and incubated at 37 °C/250 rpm overnight. Overnight cultures were diluted 100-fold in fresh TB media containing kanamycin (50 µg/mL) and 0.5 M sorbitol and grown at 37 °C/250 rpm until an OD_{600} of 0.6 - 0.8. They were subsequently induced with 0.5 mM IPTG and expressed at 25 °C/200 rpm overnight. Expression was assessed by SDS-PAGE analysis.

1.4.3 Enzyme purification and storage

Cell pellets were harvested 8000 rpm/10 mins and resuspended in 10 mL lysis buffer (50 mM Na_2HPO_4 pH 7.4, 0.5 M NaCl, 20 mM imidazole, 0.1 mM PLP). Cell suspensions were sonicated 10 sec ON/10 sec OFF for 10 cycles on ice using a Soniprep 150 and centrifuged 11,000 rpm/45 mins/4 °C. The lysates were purified by immobilised metal ion affinity chromatography via the 6x-His tag using Ni^{2+} Sepharose resin columns (Sepharose Fast Flow, GE Healthcare) and a vacuum manifold. Proteins were washed with lysis buffer containing a stepwise gradient of imidazole [20 mM (50 mL), 50 mM (50 mL), 100 mM (30 mL)] prior to elution with lysis buffer containing 500 mM imidazole (10 mL). Protein elution was monitored by a Bradford assay and protein purity was assessed by SDS-PAGE. Purified proteins were precipitated with ammonium sulfate and stored at 4 °C.

1.5 AGA C-6’ TAm deamination reactions

Prior to deamination screening the enzyme solutions were prepared as follows: An appropriate volume of ammonium sulfate precipitated protein was centrifuged 21,130 x *g*/30 mins/4 °C and resuspended in 300 µL of the assay buffer (50 mM Tris-HCl pH 8.0). This solution was transferred to a Amicon Ultra 0.5 centrifugal filter (3k MWCO, Merck Millipore) pre-equilibrated with the assay buffer and the solution was centrifuged at 13,000 x *g*/20 mins/ 4 °C. The concentrated protein solution was diluted with 350 - 400 µL of assay buffer and centrifuged once more at 13,000 x *g*/20 mins/ 4 °C. This step was repeated once again. The protein solution was collected by inverting the filter into an empty collection tube and centrifuging at 1000 x *g*/5 mins/4 °C. This solution was diluted with assay buffer to the volume required for the assay. The protein concentration was checked by a Bradford assay and then used in the reactions. Upon purification, KanB and ForB tended to aggregate and precipitate out of solution. GenB2 was also obtained in poor protein yields during protein purification. As a result, all three enzymes were tested at lower protein concentrations (ForB and GenB2: 0.1 mg/mL, KanB: 0.07 mg/mL) in deamination assays.

Deamination reactions for screening C-6’ TAms followed the conditions from Huang and co-workers[2], these were as follows: 50 mM Tris-HCl pH 8.0, 2 mM α-ketoglutarate, 0.4 mM

aminoglycoside substrate, 0.5 mM PLP, 0.1 - 0.2 mg/mL C-6' TAm, 200 µL reaction volume, incubated at 30 °C/500 rpm, 24 hrs. 10 µL samples were taken at 24 hrs post t_0, mixed with an equal volume of 0.5% TFA solution, and diluted 20-fold in MilliQ H_2O. Samples were either analysed immediately or frozen at -20 °C prior to analysis by HPAE-IPAD. Aminoglycoside substrates tested: neamine trihydrochloride, kanamycin A sulfate, kanamycin B sulfate, tobramycin, gentamicin C1a, sisomicin sulfate, ribostamycin sulfate, amikacin. The salt content was accounted for when determining substrate concentrations in assays.

1.6 GenB4 isomerisation assay and reaction optimisation

A typical GenB4 isomerisation assay slightly modified the conditions from Chen and colleagues[3], The final reaction conditions were as follows: 50 mM potassium phosphate (KPi) buffer pH 8.0, 2 mM sisomicin, 0.5 mM PLP, 0.2 mg/mL GenB4 enzyme prepared as described above in section 1.5.

The reaction was optimised in terms of enzyme concentration, amine acceptor necessity, reaction pH, temperature and sisomicin concentration. For enzyme concentration, the assay conditions from above were used in the presence of 0.03, 0.06, 0.125 and 0.25 mg/mL GenB4 enzyme in triplicate. The assay with and without 2 mM α-ketoglutarate followed the conditions described with and without 2 mM a-ketoglutarate and 0.125 mg/mL GenB4. The reaction pH was varied from pH 7.0, 7.5, 8.0, 8.5 and 9.0 using KPi buffer and performed in duplicate. The temperature assay was performed in duplicate and reactions were incubated at 20 °C, 30 °C, 40 °C, 50 °C and 60 °C. Finally, the sisomicin concentration assay was performed in duplicate using the conditions described and varying the sisomicin concentration: 1, 2, 5, and 10 mM of sisomicin.

All optimisation reactions were performed on a 200 µL scale. Enzyme concentration and amine acceptor necessity reactions were incubated for 2 hrs and 10 µL samples were taken at 0, 15, 30, 60 and 120 mins of reaction. All other reactions were incubated for 24 hrs and 10 µL samples were taken at 0, 60, 120, 240 and 1440 mins of reaction time. All reaction samples were mixed with an equivalent volume of 0.5% TFA solution and frozen at -20 °C. Prior to analysis by HPAE-IPAD, samples were thawed, centrifuged 1000 *x g*/4 °C/2 mins and diluted 62.5-fold in MilliQ H_2O.

1.7 Analysis of GenB4 reaction side product formation

A GenB4 reaction was set-up in triplicate (200 µL reactions) as described in section 1.6. After 1 hr of incubation at 30 °C, the reactions were incubated at 95 °C for 1 hr and a 10 µL sample was taken. The reactions were then further incubated at 30 °C for 23 hrs, and a second 10 µL sample was taken. Samples were processed for HPAE-IPAD analysis as described in section 1.5. For LC-MS analysis, a GenB4 reaction with sisomicin was prepared using the conditions described in 1.6 and incubated for 2 hrs at 35 °C. The reaction was then heated at 95 °C for 30 mins. The pH was reduced to 5.0 with AcOH and 10 equivalents of polymer supported cyanoborohydride (polymer = Amberlite IRA-400) was added and this mixture was incubated at 25 °C/1000 rpm/17 hrs. A sample of the reaction was diluted 25-fold and analysed by LC-MS using the method described in section 1.14.2.

1.8 Reductive amination pH assay

GenB4 enzyme solution was prepared as described in section 1.5 and added to a final concentration of 0.3 mg/mL in a reaction (6.5 mL) containing 50 mM NaPi buffer pH 8.0, 0.5 mM PLP and 2.5 mM sisomicin. The reaction was incubated for 1 hr at 30 °C/175 rpm. After 1 hr, an equal volume of cold methanol was added directly to the enzymatic reaction and the

mixture was centrifuged 10,000 rpm/4 °C for 5 minutes to remove the protein from the reaction mixture. The supernatant was transferred to a new tube. The reaction was then split into fractions in triplicate and the pH adjusted to 4.0, 5.0, 6.0 and 7.0 with concentrated AcOH, giving 12 fractions in total. Ten equivalents of phenethylamine (**3g**) were added respectively and the reaction stirred for 1 hr at 25 °C. Ten equivalents of polymer-supported cyanoborohydride was added and the reaction was further incubated at 25 °C for 16 - 17 hrs. In a separate experiment, The same reaction procedure was followed, but 20 equivalents of benzylamine was added instead of 10 equivalents of phenethylamine.

After incubation, the reactions containing phenethylamine (**3g**) were diluted 25-fold and analysed by LC-MS using the method described in section 1.14.2. For the benzylamine (**3f**) reactions, the reactions were briefly centrifuged, 800 μL of the supernatant was separated from the beads and MeOH from the reaction was removed by centrifugal evaporation for 4 hrs at room temperature. The remaining aqueous phase was made up to 400 μL with 50 mM NaPi buffer pH 8.0 and the pH was adjusted to 10.0 - 11.0 with 12 M NaOH. The excess benzylamine was extracted with EtOAc (3 x 400 μL) and the pH of the aqueous phase was re-acidified to pH 7.0 - 8.0 with 1 M HCl. These solutions were analysed by analytical HPLC. For conditions see section 1.14.3 and Table S4. Samples were kept at -20 °C prior to analysis.

1.9 Reductive amination methanol content assay

GenB4 enzyme solution was prepared as described in section 1.5 and added to a final concentration of 0.1 mg/mL in a reaction containing 50 mM NaPi buffer pH 8.0, 0.5 mM PLP and 2.5 mM sisomicin. The reaction was incubated for 3 hrs at 30 °C/175 rpm. The reaction was then split into 5 x 500 μL equal fractions. An equal volume of cold MeOH was added to one of the fractions to give a final MeOH content of 50%. For the remaining fractions, the volume of water was reduced by rotary evaporation. The volumes were then adjusted to 400 μL, 300 μL, 200 μL and 100 μL and 600 μL, 700 μL, 800 μL and 900 μL of cold MeOH was added respectively to give final methanol contents of 60%, 70%, 80% and 90%. All samples were adjusted to pH 7.0 with AcOH, 15 equivalents of phenethylamine was added and reactions were incubated for 1 hr/25 °C/1000 rpm. Ten equivalents of polymer-supported cyanoborohydride was added and the reaction was further incubated at 25 °C for 16 - 17 hrs. After incubation, the reactions were diluted 50-fold and analysed by LC-MS using the method described in section 1.14.2.

1.10 Substrate solubility tests

GenB4 enzyme solution was prepared as described in section 1.5 and the reaction with sisomicin was performed in triplicate (3 x 500 μL) as described in section 1.6. After 1 hr of incubation, 10 μL of the reaction was sampled and mixed with an equivalent volume of 0.5% TFA. To the remaining reactions, an equal volume of cold methanol was added, 10 μL of the reaction was sampled and mixed with an equivalent volume of 0.5% TFA. These samples were analysed by HPAE-IPAD using the method for 6’-oxogentamicin C1a, see section 1.14.1.

For gentamicin C1a solubility tests, 9 x 100 μL solutions of 12.5 mM gentamicin C1a in 200 mM NaPi buffer pH 5.0 were prepared in triplicate (27 solutions total). Three solutions were kept at 100 μL. The remaining solutions were diluted with water in triplicate to 200 μL, 300 μL, 400 μL, 500 μL, 600 μL, 700 μL, 800 μL and 900 μL final volumes. To these solutions, cold methanol was added to give a final volume of 1 mL in each solution. These solutions were centrifuged for 2 mins at 10,000 x *g*/4 °C. Samples were diluted 62.5-fold and analysed by HPAE-IPAD using a 3 mM KOH isocratic method for 20 mins.

1.11 Reductive amination conversion assay

GenB4 enzyme solution was prepared as described in section 1.5 and a 7.5 mL reaction was performed as described in section 1.6. After 1 hr, an equal volume of cold methanol was added and the mixture was centrifuged 10000 rpm/4°C for 5 minutes to remove the protein from the reaction mixture. The supernatant was transferred to a new tube. The reaction was split into 14 x 500 µL fractions and adjusted to pH 5.0 with concentrated AcOH. Ten equivalents of amines **3a**, **3f** - **3r** were added and reactions were stirred for 1 hr at 25 °C/1000 rpm. Ten equivalents of polymer-supported cyanoborohydride was added and the reaction was further incubated at 25 °C for 17 - 18 hrs. Samples were subsequently diluted 25-fold in water and analysed by LC-MS using the method described in section 1.14.2. Amines **3b** - **3e** (Table 2.15) were assayed on a separate day, a larger scale GenB4 reaction (20 mL) was set-up as above and split into 4 x 5 mL fractions. The procedure then followed as described above, 10 equivalents of each amine were added and samples for LC-MS analysis were taken after 18 hrs of incubation with PS-CBH.

For HPLC determination of reaction conversion, the GenB4 reaction was performed, methanol added and the pH adjusted to pH 5.0 as described above. The reaction was split into fractions and 10 equivalents of 4-chloroaniline (**3d**), 20 equivalents of benzylamine (**3f**), phenethylamine (**3g**), furfurylamine (**3j**) or 25 equivalents of tyramine (**3h**) were added and reactions were stirred for 1 hr at 25 °C. Ten equivalents of polymer-supported cyanoborohydride was added and the reaction further incubated at 25 °C for 41 hrs. After incubation, the reactions were briefly centrifuged, 800 µL of the supernatant was separated from the beads and the MeOH from the reaction was removed by centrifugal evaporation for 4 - 5 hrs at room temperature. The remaining aqueous phase was basified to pH 10.0 - 11.0 with 12 M NaOH and the excess amine was extracted with EtOAc (3 x 400 µL). The pH of the aqueous phase was re-acidified to pH 7.0 - 8.0 with 1 M HCl and these solutions were analysed by analytical HPLC. Variations in sample volume due to pH adjustments were recorded and accounted for to determine final analyte concentrations prior to HPLC analysis. For HPLC conditions see section 1.14.3 and Table S4. Samples were kept at -20 °C prior to analysis.

1.12 Antimicrobial activity screening of C-6’ gentamicin C1a derivatives

1.12.1 Minimal inhibitory concentration determination

Minimal inhibitory concentration assays were performed according to the guidelines specified in the literature[4]. Assays were performed in triplicate in sterile 96-well plates. The following wild-type bacteria were streaked onto LB agar: *Escherichia coli* ATCC 25922, *Pseudomonas aeruginosa* ATCC 27853, *Staphylococcus aureus* ATCC 29213, *Salmonella typhimurium* LT2, *Bacillus subtilis* RM125 and *Klebsiella pneumoniae*. Four colonies of each strain were inoculated in Mueller Hinton broth and incubated overnight at 37 °C/250 rpm. The overnight culture was diluted 100-fold and incubated until the cells reached log-phase. At this point the cells were diluted to an OD_{625} approximately equivalent to a CFU/mL of 1 x 10^8 and then diluted 100-fold to give a concentration of approximately 1 x 10^6 CFU/mL. All dilutions were done in Mueller Hinton broth.

C-6’ gentamicin C1a derivatives were dissolved in MilliQ H_2O to a final concentration of 2 mg/mL (free base basis) and then diluted to the following concentrations dependent on the bacterium being tested: *E. coli* and *S. typhimurium* - 4 µg/mL, *P. aeruginosa* and *S. aureus* - 16 µg/mL, *B. subtilis* and *K. pneumoniae* - 1 µg/mL in Mueller Hinton broth. These solutions were subsequently serially two-fold diluted 6 times in Mueller Hinton broth in each well of the

microtiter plate to give the antibiotic concentrations ranges tested. An equivalent volume of cells were added to each well to a final cell concentration of approximately 5 x 10^5 CFU/mL. All plates contained a negative control which had no cells, a positive control lacking antibiotic and a gentamicin C1a positive control in triplicate to control for interplate variation. Plates were sealed with Breathe-Easier sealing film (Diversified Biotech) and incubated at 37 °C static overnight. After incubation, the OD_{600} was measured. For MIC determination, the turbidity measurements were background subtracted with the negative control, the turbidity of each well was averaged across replicates and expressed as a percentage turbidity compared to the positive control lacking antibiotic. The antibiotic concentration which caused a greater than 90% reduction in turbidity compared to control lacking antibiotic was taken as the MIC.

1.12.2 Generation of *E. coli* ATCC 25922 strains carrying resistance plasmids and testing

Chemically competent cells of *E. coli* ATCC 25922 were transformed with resistance plasmids R26 (80 kb, carrying an *aac(3)-I* gene), pSa (37 kb, carrying an *aac(6')-Ib* gene) and pQR1865 (A *Bgl*II fragment of pSa which contains the *aac(6')-Ib* and *ant(3")* genes). Transformants were screened for the presence of each plasmid by mini-prep and restriction digestion using *Not*I for R26 and *Hind*III for pSa and pQR1865. Transformants were also screened for antibiotic resistance on LB media containing 10 µg/mL gentamicin C1a, 50 µg/mL kanamycin or 100 µg/mL streptomycin and grown at 37 °C for 24 hrs.

Antimicrobial activity testing was performed in triplicate against the resistant *E. coli* strains as described above in section 1.12.1. The antibiotic concentration range tested was 32 µg/mL - 0.0625 µg/mL.

1.12.3 Antimicrobial activity screening on solid media

E. coli ATCC 25922, *E. coli* ATCC 25922 + pSa, *E. coli* ATCC 25922 + R26, *E. coli* ATCC 25922 + pQR1865 were grown in LB overnight at 37 °C/250 rpm, diluted to an OD_{600} of 0.141 and 100 µL was spread onto LB solid media containing 2 % agar and dried. Using a flame-sterilised metal cork borer, wells were created in the media, and 100 µL of test antibiotics (0.1 mg/mL aqueous solutions) were added to each well. Gentamicin C1a was used as a positive control. Plates were incubated upright at 37 °C for 1 day, after which antibiotic activity was assessed for the presence and size of zones of clearing.

1.13 Aminoglycoside purification methods

1.13.1 Tetraphenylborate precipitation

The method used was adapted from that reported by Katano and co-workers[5]. The residues obtained from work-up of the GenB4 + reductive amination reactions were dissolved in 500 µL of H_2O and mixed with an equal of volume of 300 mM sodium tetraphenylborate aqueous solution. The white precipitate formed was centrifuged at max. speed for 1.5 mins and the supernatant discarded. The precipitate was washed 3 times with 1 mL of H_2O, centrifuging at the max. speed each time, and removing the supernatant. The pellet obtained was dissolved in 1 mL of acetone by vortexing and then an additional 1 mL of acetone was added. 50 µL of 5 M HCl(aq.) solution was added dropwise and a white to off-white precipitate formed after centrifugation. This pellet was washed 3 times with 1 mL of acetone and then dried by rotary evaporation to obtain a crudely purified aminoglycoside product as the HCl salt.

1.13.2 Ion exchange resin exchange procedures

Exchange of Amberlite CG50(H^+) resin for CG-50(NH_4^+) form

The method used follows that described by Chen and colleagues[6]. Amberlite CG-50(H^+ form) resin (Type I, 100 - 200 mesh, Sigma-Aldrich) was suspended in 5 times the resin volume of 1 M NH_4OH(aq.) solution and shaken at 150 rpm for 4 hrs at room temperature. The resin was then washed with 5 times the resin volume of 1 M HCl(aq.) solution followed by another 5 times volume of 1 M NH_4OH(aq.) solution. Between resin acid-base wash steps the resin was washed with deionised water to remove residual NH_4OH and HCl until the pH of the solution was near neutral. The resin was collected by vacuum filtration and stored at room temperature in a glass bottle.

Exchange of Amberchrom 1 x 2 (Cl^-) resin for 1 x 2 (OH^-) form

The method used follows that described by Umezawa and Kondo[7]. Amberchrom 1 x 2 (Cl^- form) resin (200 - 400 mesh, Sigma-Aldrich) was transferred to a large glass column using deionised water and then washed with 2 resin volumes of 1% Na_2SO_4 solution followed by 10 resin volumes of 10 % NaOH(aq.) solution. The resin was then washed with deionised water until the pH of the eluted solution was near neutral. The resin was dried and stored in a glass bottle at room temperature.

1.14 Analytical Procedures

1.14.1 High performance anion exchange chromatography with integrated pulsed amperometric detection (HPAE-IPAD)

HPAE-IPAD analysis was performed using a Reagent-Free Ion Chromatography System (ICS) (ICS-5000+ system, Thermo Scientific) fitted with CarboPac PA1 (4 x 250 mm) anion exchange column with a CarboPac PA1 guard column (4 x 50 mm), an Eluent Generator with a KOH 500 cartridge and an electrochemical detector (gold electrode). The flow rate was 1 mL/min and column temperature was 30 °C. The sample tray was cooled to 4 °C. An injection volume of 10 µL was used and samples were prepared in 96-well polypropylene plates. Analytical methods used are described in Table S3.

1.14.2 Liquid chromatography - mass spectrometry

Low resolution liquid-chromatography tandem mass spectrometry (LC-MS/MS) analyses were performed on a Hypersil GOLD C_{18} column (150 x 2.1 mm, 1.9 µm particle size, Thermo Scientific) fitted on an Accela 600 LC system connected to a Finnigan LTQ Linear ion trap mass spectrometer. Analyses were performed in polypropylene vials (Waters). The mobile phases were A: H_2O + 0.1 % pentafluoropropionic acid (PFPA), B: MeCN + 0.1 % PFPA and the gradient method previously reported[8] was adapted: flow rate: 200 µL/min, column temperature: 40 °C, 90:10 A:B to 50:50 A:B, 0 - 20 mins, 50:50 A:B to 90:10 A:B 20 - 20.1 mins, 90:10 A:B, 20.1 - 25 mins.

1.14.3 Analytical high pressure liquid chromatography (HPLC)

Analytical HPLC analysis was performed on an Agilent 1260 Infinity analytical LC system fitted with an C_{18}-AR (4.6 x 150 mm, ACE) analytical column and guard column. The injection volume was 10 µL, the detection wavelength was 214 nm and the column temperature was 25 °C. The mobile phases were A: H_2O + 0.1 % trifluoroacetic acid, B: MeOH. Compound specific gradient methods are described in Table S4.

1.14.4 Semi-preparative HPLC

Semi-preparative HPLC was performed on an Agilent 1260 Infinity II preparative LC system fitted with an Agilent ZORBAX 300SB-C_{18} (250 x 9.4 mm, 5 µm particle size) column. The mobile phases were A: H_2O + 0.1 % trifluoroacetic acid, B: MeOH or MeCN. Preparative separations were performed at room temperature with a flow rate of 4.2 mL/min. Samples were prepared in polypropylene vials (Waters) and fractions were collected in 13 x 100 mm polypropylene tubes (Greiner Bio-One). Compound specific gradient methods used for preparative LC are described in Table S5.

1.15 General synthesis of 6'-gentamicin C1a derivatives

GenB4 enzyme solution was prepared as described in section 1.5 and added to a final concentration of 0.3 - 0.5 mg/mL in a reaction containing 50 mM NaPi buffer pH 8.0, 0.5 mM PLP and 2.5 mM sisomicin. The reaction was incubated for 1 hr at 30 °C/175 rpm. After 1 hr, an equal volume of cold MeOH was added and the mixture was centrifuged 10,000 rpm/4°C for 5 minutes to remove the protein from the reaction mixture. The supernatant was transferred to a new Falcon tube and the pH adjusted to 5.0 with concentrated AcOH. 10 - 25 Equivalents of amine were added, and the reaction stirred for 1 hr at 25 °C. Ten equivalents of polymer-supported cyanoborohydride was added and the reaction further incubated at 25 °C for 17 - 48 hrs. Reaction progress was monitored by LC-MS as described in section 1.14.2. After incubation, the beads were separated from the reaction mixture and the reaction solvent was removed by rotary evaporation. The remaining residue was washed with EtOAc (3 x 1 mL) to remove excess amine. The compounds were then further purified as specified below for each compound. For NMR and mass spectrometry spectra see Appendices.

Prior to activity testing all compounds were converted to hydrochloride salts by dissolution in 0.2 M HCl solution and freeze-drying. Gentamicin C1a free base obtained from Carbosynth was also converted to a HCl salt by this same process. The products were hygroscopic and were stored under argon at -20 °C.

6'-*N*-(Phenyl)-gentamicin C1a (4a)

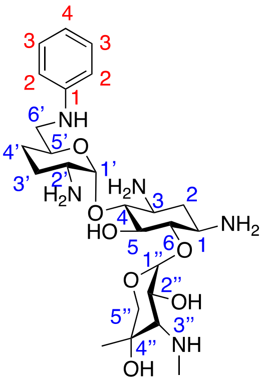

Ten equivalents of aniline (**3a**) were added to the reaction and the reductive amination was incubated for 17 hrs. The product was purified by semi-preparative HPLC to give **4a** as a pale yellow crystalline solid. Isolated yield: 55%. **^{1}H NMR** (600 MHz; D_2O) δ 7.56 (2H, t, *J* = 7.8 Hz, Ar-H), 7.48 (1H, t, *J* = 7.8 Hz, Ar 4-H), 7.42 (2H, d, *J* = 7.8 Hz, Ar-H), 5.82 (1H, d, *J* = 3.6 Hz, 1'-H), 5.10 (1H, d, *J* = 3.8 Hz, 1"-H), 4.35 - 4.28 (1H, m, 5'-H), 4.25 (1H, dd, *J* = 10.9, 3.8 Hz, 2"-H), 4.05 - 3.95 (2H, m, 5"-H_a, 4/6-H), 3.83 (1H, t, *J* = 9.4 Hz, 5-H), 3.78 (1H, t, *J* = 9.4 Hz, 4/6-H), 3.67 - 3.47 (7H, m), 2.93 (3H, s, 3"-*N*-CH_3), 2.55 (1H, dt, *J* = 12.6, 4.3 Hz, 2-H_{eq}), 2.10 - 1.90 (4H, m, 3'-H_2, 4'-H, 2-H_{ax}), 1.74 - 1.65 (1H, m, 4'-H), 1.36 (3H, s, 4"-CH_3); **^{13}C NMR** (150

MHz; D_2O) δ 137.7, 131.0, 128.9, 121.6, 102.0, 96.3, 84.4, 78.2, 74.9, 70.5, 68.5, 66.9, 66.8, 63.9, 54.4, 50.2, 49.4, 49.0, 35.1, 28.5, 26.3, 21.5, 21.2; ***m/z*** [MS ES+] R(*t*): 11.8 min, 526 $[M + H]^+$, 548 $[M + Na]^+$; [MS/MS ES+ of *m/z* 526] 509, 367, 322, 205, 160; [HRMS ES+] Found $[M + H]^+$ 526.3234; $C_{25}H_{43}N_5O_7$ requires 526.3235.

6’-*N*-(2-Chlorophenyl)-gentamicin C1a (4b)

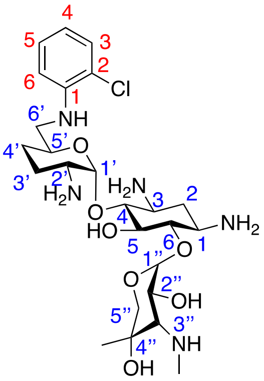

Ten equivalents of 2-chloroaniline (**3b**) were added to the reaction and the reductive amination was incubated for 43 hrs. The product was purified by semi-preparative HPLC to give **4b** as a yellow crystalline solid. Isolated yield: 11%. **^{1}H NMR** (600 MHz; D_2O) δ 7.41 (1H, dd, *J* = 7.9, 1.3 Hz, Ar 3-H), 7.28 (1H, td, *J* = 7.9, 1.3 Hz, Ar 5-H), 6.98 (1H, dd, *J* = 7.9, 1.3 Hz, Ar 6-H), 6.85 (1H, td, *J* = 7.9, 1.3 Hz, Ar 4-H), 5.52 (1H, d, *J* = 3.7 Hz, 1’-H), 5.09 (1H, d, *J* = 3.9 Hz, 1”-H), 4.24 (1H, dd, *J* = 10.9, 3.9 Hz, 2”-H), 4.20 - 4.15 (1H, m, 5’-H), 4.01 (1H, d, *J* = 12.8 Hz, 5”-H_a), 3.84 (1H, t, *J* = 9.4 Hz, 4/6-H), 3.77 (1H, t , *J* = 9.4 Hz, 5-H), 3.73 (1H, t , *J* = 9.4 Hz, 4/6-H), 3.62 (1H, dt, *J* = 11.8, 3.7 Hz, 2’-H), 3.57 - 3.47 (4H, m, 5”-H_b, 1-H, 3-H, 3”-H), 3.45 - 3.37 (2H, m, 6’-H_2), 2.93 (3H, s, 3”-*N*-CH_3), 2.50 (1H, dt, *J* = 12.8, 4.3 Hz, 2-H_{eq}), 2.11 - 2.04 (1H, m, 3’/4’-H), 2.03 - 1.95 (2H, m, 3’/4’-H), 1.85 (1H, q, *J* = 12.8 Hz, 2-H_{ax}), 1.70 - 1.61 (1H, m, 4’-H), 1.36 (3H, s, 4”-CH_3); **^{13}C NMR** (150 MHz; D_2O) δ 144.1, 130.3, 128.9, 120.6, 120.1, 114.1, 101.9, 97.3, 84.2, 80.5, 74.4, 70.5, 70.4, 68.4, 66.8, 63.9, 50.1, 49.7, 49.4, 48.0, 35.1, 28.3, 26.1, 21.9, 21.5; ***m/z*** [MS ES+] R(*t*): 13.2 min, 560 $[M + H]^+$, 582 $[M + Na]^+$; [MS/MS ES+ of *m/z* 560] 543, 401, 322, 205, 163, 160; [HRMS ES+] Found $[M + H]^+$ 560.2853; $C_{25}H_{42}ClN_5O_7$ requires 560.2845.

6’-*N*-(3-Chlorophenyl)-gentamicin C1a (4c)

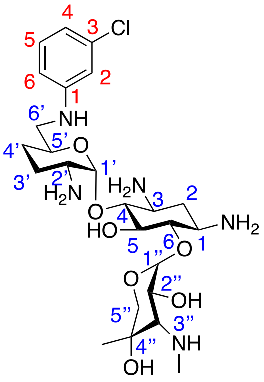

Ten equivalents of 3-chloroaniline (**3c**) were added to the reaction and the reductive amination was incubated for 18 hrs. The product was purified by semi-preparative HPLC to give **4c** as a yellow crystalline solid. Isolated yield: 42%. **^{1}H NMR** (600 MHz; D_2O) δ 7.36 (1H, t, *J* = 8.0 Hz, Ar 5-H), 7.18 - 7.14 (2H, m, Ar 4-H, Ar 2-H), 7.04 (1H, dd, *J* = 8.0, 1.6 Hz, Ar 6-H), 5.66 (1H, d, *J* = 3.6 Hz, 1'-H), 5.10 (1H, d, *J* = 3.8 Hz, 1"-H), 4.27 - 4.18 (2H, m, 5'-H, 2"-H), 4.01 (1H, d , *J* = 12.8 Hz, 5"-H_a), 3.92 (1H, t , *J* = 9.2 Hz, 4/6-H), 3.80 (1H, t, *J* = 9.2 Hz, 5-H), 3.76 (1H, t, *J* = 9.2 Hz, 4/6-H), 3.63 - 3.45 (6H, m, 2'-H, 1-H, 3-H, 6'-H_a, 3"-H, 5"-H_b), 3.41 (1H, dd, *J* = 14.8, 7.7 Hz, 6'-H_b), 2.93 (3H, s, 3"-*N*-CH_3), 2.53 (1H, dt, *J* = 12.7, 4.3 Hz, 2-H_{eq}), 2.09 - 1.95 (3H, m, 3'-H_2, 4'-H), 1.91 (1H, q, *J* = 12.7 Hz, 2-H_{ax}), 1.70 - 1.60 (1H, m, 4'-H), 1.36 (3H, s, 4"-CH_3); **^{13}C NMR** (150 MHz; D_2O) δ 144.6, 135.6, 131.8, 123.8, 117.8, 116.6, 101.9, 96.8, 84.3, 79.5, 74.6, 70.5, 68.6, 68.5, 66.8, 63.9, 51.1, 50.2, 49.6, 49.2, 35.1, 28.4, 26.2, 21.6, 21.5; ***m/z*** [MS ES+] R(*t*): 13.8 min, 560 $[M + H]^+$, 582 $[M + Na]^+$; [MS/MS ES+ of *m/z* 560] 543, 401, 322, 205, 163, 160; [HRMS ES+] Found $[M + H]^+$ 560.2846; $C_{25}H_{42}ClN_5O_7$ requires 560.2845.

6'-*N*-(4-Chlorophenyl)-gentamicin C1a (4d)

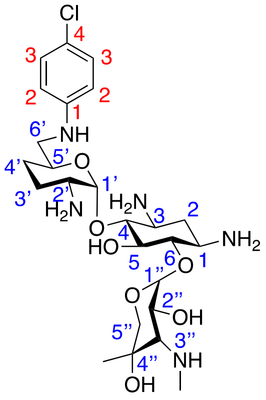

Ten equivalents of 4-chloroaniline (**3d**) were added to the reaction and the reductive amination was incubated for 18 hrs. The product was purified by semi-preparative HPLC to give **4d** as a yellow crystalline solid. Isolated yield: 41%. **^{1}H NMR** (600 MHz; D_2O) δ 7.57 (2H, d, *J* = 8.8 Hz, Ar 3-H), 7.42 (2H, d, *J* = 8.8 Hz, Ar 2-H), 5.83 (1H, d, *J* = 3.6 Hz, 1'-H), 5.09 (1H, d, *J* = 3.8 Hz, 1"-H), 4.36 - 4.28 (1H, m, 5'-H), 4.24 (1H, dd, *J* = 10.9, 3.8 Hz, 2"-H), 4.04 - 3.96 (2H, m, 5"-H_a, 4/6-H), 3.83 (1H, t, *J* = 9.4 Hz, 5-H), 3.77 (1H, t, *J* = 9.4 Hz, 4/6-H), 3.65 - 3.54 (5H, m, 2'-H, 1-H, 3-H, 6'-H_2), 3.52 (1H, d, *J* = 12.9 Hz, 5"-H_b), 3.48 (1H, d, *J* = 10.9 Hz, 3"-H), 2.92 (3H, s, 3"-*N*-CH_3), 2.55 (1H, dt, *J* = 12.6, 4.3 Hz, 2-H_{eq}), 2.08 - 2.03 (2H, m, 3'/4'-H), 2.02 - 1.90 (2H, m, 3'/4'-H, 2-H_{ax}), 1.73 - 1.63 (1H, m, 4'-H), 1.35 (3H, s, 4"-CH_3); **^{13}C NMR** (150 MHz; D_2O) δ 135.9, 134.5, 131.0, 123.5, 102.0, 96.2, 84.4, 78.1, 74.9, 70.5, 68.5, 66.9, 66.8, 63.9, 54.7, 50.2, 49.3, 49.0, 35.1, 28.5, 26.3, 21.5, 21.2; ***m/z*** [MS ES+] R(*t*): 13.8 min, 560 $[M + H]^+$, 582 $[M + Na]^+$; [MS/MS ES+ of *m/z* 560] 543, 401, 322, 205, 163, 160; [HRMS ES+] Found $[M + H]^+$ 560.2855; $C_{25}H_{42}ClN_5O_7$ requires 560.2845.

6'-*N*-(Pyridin-3-ylamino)-gentamicin C1a (4e)

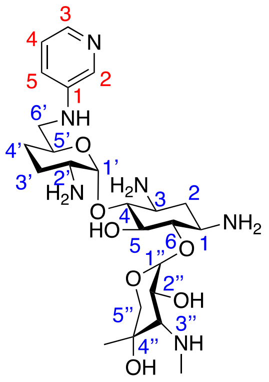

20 equivalents of 3-aminopyridine (**3e**) were added to the reaction and the reductive amination was incubated for 43 hrs. The product was purified by semi-preparative HPLC to give **4e** as a yellow to orange crystalline solid. Isolated yield: 20%. **^{1}H NMR** (600 MHz; D_2O) δ 8.06 (1H, d, *J* = 2.8 Hz, Ar 2-H), 7.98 (1H, d, *J* = 5.5 Hz, Ar 5-H), 7.80 (1H, dd, *J* = 8.9, 2.8 Hz, Ar 3-H), 7.72 (1H, dd, *J* = 8.9, 5.5 Hz, Ar 4-H), 5.60 (1H, d, *J* = 3.5 Hz, 1'-H), 5.09 (1H, d, *J* = 3.7 Hz, 1"-H), 4.24 (1H, dd, *J* = 10.9, 3.7 Hz, 2"-H), 4.21 - 4.15 (1H, m, 5'-H), 4.01 (1H, d, *J* = 12.8 Hz, 5"-H_a), 3.89 (1H, t, *J* = 9.4 Hz, 4/6-H), 3.80 (1H, t, *J* = 9.4 Hz, 5-H), 3.75 (1H, t, *J* = 9.4 Hz, 4/6-H), 3.61 - 3.51 (4H, m, 2'-H, 1-H, 3-H, 5"-H_b), 3.51 - 3.45 (2H, m, 6'-H_a, 3"-H), 3.39 (1H, dd, *J* = 14.3, 6.6 Hz, 6'-H_b), 2.93 (3H, s, 3"-*N*-CH_3), 2.54 (1H, dt, *J* = 12.6, 4.3 Hz, 2-H_{eq}), 2.08 - 1.99 (2H, m, 3'/4'-H), 1.99 - 1.89 (2H, m, 3'/4'-H, 2-H_{ax}), 1.76 - 1.67 (1H, m, 4'-H), 1.36 (3H, s, 4"-CH_3); **^{13}C NMR** (150 MHz; D_2O) δ 148.4, 129.3, 129.1, 127.7, 125.1, 101.9, 97.0, 84.3, 79.6, 74.6, 70.5, 70.3, 68.5, 66.8, 63.9, 50.2, 49.6, 49.3, 46.7, 35.1, 28.3, 25.8, 21.6, 21.5; ***m/z*** [MS ES+] R(*t*): 9.8 min, 527 $[M + H]^+$, 549 $[M + Na]^+$; [MS/MS ES+ of *m/z* 527] 510, 368, 322, 206, 160; [HRMS ES+] Found $[M + H]^+$ 527.3191; $C_{24}H_{42}N_6O_7$ requires 527.3188.

6'-*N*-(Benzyl)-gentamicin C1a (4f)

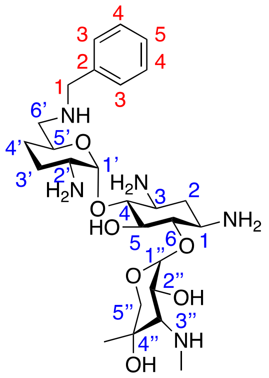

20 equivalents of benzylamine (**3f**) were added to the reaction and the reductive amination was incubated for 48 hrs. The product was purified by semi-preparative HPLC to give **4f** as a white to off-white crystalline solid. Isolated yield: 29%. **^{1}H NMR** (600 MHz; D_2O) δ 7.52 (5H, m, Ar-H), 5.79 (1H, d, *J* = 3.5 Hz, 1'-H), 5.08 (1H, d, *J* = 3.8 Hz, 1"-H), 4.36 - 4.26 (2H, m, *N*-Ar 1-H_2), 4.24 (1H, dd, *J* = 10.9, 3.8 Hz, 2"-H), 4.21 - 4.16 (1H, m, 5'-H), 4.00 (1H, d, *J* = 12.8 Hz, 5"-H_a), 3.93 (1H, t, *J* = 9.5 Hz, 4/6-H), 3.80 (1H, t, *J* = 9.5 Hz, 5-H), 3.74 (1H, t, *J* = 9.5 Hz, 4/6-H), 3.60 - 3.46 (5H, m), 3.30 (1H, dd, *J* = 13.3, 3.2 Hz, 6'-H_a), 3.20 (1H, dd, *J* = 13.3, 7.8 Hz, 6'-H_b), 2.93 (3H, s, 3"-*N*-CH_3), 2.53 (1H, dt, *J* = 12.5, 4.3 Hz, 2-H_{eq}), 2.06 - 1.98 (2H, m, 3'/4'-H), 1.96 - 1.86 (2H, m, 3'/4'-H, 2-H_{ax}) 1.66 - 1.56 (1H, m, 4'-H), 1.36 (3H, s, 4"-CH_3); **^{13}C NMR** (150 MHz; D_2O) δ 130.8, 130.6, 130.5, 129.9, 102.0, 95.9, 84.5, 77.5, 74.9, 70.5, 68.5,

66.8, 66.2, 63.9, 52.4, 50.6, 50.2, 49.3, 48.9, 35.0, 28.4, 26.4, 21.5, 20.9; ***m/z*** [MS ES+] R(*t*): 12.0 min, 540 [M + H]$^+$, 562 [M + Na]$^+$; [MS/MS ES+ of *m/z* 540] 523, 381, 322, 219, 160; [HRMS ES+] Found [M + H]$^+$ 540.3392; $C_{26}H_{45}N_5O_7$ requires 540.3392.

6'-*N*-(Phenethyl)-gentamicin C1a (4g)

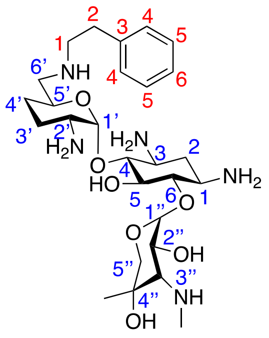

20 equivalents of phenethylamine (**3g**) were added to the reaction and the reductive amination was incubated for 41 hrs. The product was purified by tetraphenylborate precipitation and semi-preparative HPLC to give **4g** as a white crystalline solid. Isolated yield: 10%. **^{1}H NMR** (600 MHz; D_2O) δ 7.44 (2H, m, Ar-H), 7.40 - 7.33 (3H, m, Ar-H), 5.80 (1H, d, *J* = 3.6 Hz, 1'-H), 5.10 (1H, d, *J* = 3.8 Hz, 1"-H), 4.25 (1H, dd, *J* = 10.9, 3.8 Hz, 2"-H), 4.24 - 4.18 (1H, m, 5'-H), 4.02 (1H, d, *J* = 12.8 Hz, 5"-H_a), 3.97 (1H, t, *J* = 9.5 Hz, 4/6-H), 3.82 (1H, t, *J* = 9.5 Hz, 5-H), 3.77 (1H, t, *J* = 9.5 Hz, 4/6-H), 3.62 - 3.52 (4H, m, 5"-H_b, 1-H, 3-H, 2'-H), 3.50 (1H, d, *J* = 10.9, 3"-H), 3.40 - 3.35 (2H, m, Ar 1-H_2), 3.33 (1H, dd, *J* = 13.3, 3.3 Hz, 6'-H_a), 3.24 (1H, dd, *J* = 13.3, 7.4 Hz, 6'-H_b), 3.11 - 3.04 (2H, m, Ar 2-H_2), 2.94 (3H, s, 3"-*N*-CH_3), 2.56 (1H, dt, *J* = 12.6, 4.3 Hz, 2-H_{eq}), 2.06 - 2.00 (2H, m, 3'/4'-H), 2.00 - 1.91 (2H, m, 3'/4'-H, 2-H_{ax}) 1.70 - 1.58 (1H, m, 4'-H), 1.37 (3H, s, 4"-CH_3); **^{13}C NMR** (150 MHz; D_2O) δ 136.9, 129.7, 129.4, 128.1, 102.0, 96.0, 84.5, 77.7, 75.0, 70.5, 68.5, 66.8, 66.2, 63.9, 51.2, 50.2, 50.0, 49.3, 48.9, 35.1, 32.0, 28.5, 26.3, 21.5, 21.0; ***m/z*** [MS ES+] R(*t*): 13.3 min, 554 [M + H]$^+$, 576 [M + Na]$^+$; [MS/MS ES+ of *m/z* 554] 537, 395, 322, 233, 160; [HRMS ES+] Found [M + H]$^+$ 554.3549; $C_{27}H_{47}N_5O_7$ requires 554.3548.

6'-*N*-(4-hydroxyphenethyl)-gentamicin C1a (4h)

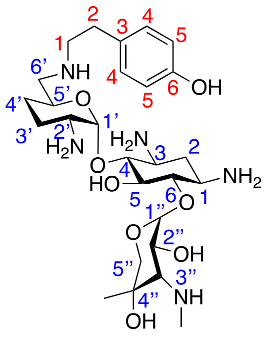

25 equivalents of tyramine (**3h**) were added to the reaction and the reductive amination was incubated for 48 hrs. 2-propanol was used to remove excess amine instead of EtOAc. The product was purified by tetraphenylborate precipitation and semi-preparative HPLC to give **4h** as a white to off-white crystalline solid. Isolated yield: 6%. **^{1}H NMR** (600 MHz; D_2O) δ 7.22 (2H, d, *J* = 8.4 Hz, Ar 4-H), 6.91 (2H, d, *J* = 8.4 Hz, Ar 5-H), 5.79 (1H, d, *J* = 3.6 Hz, 1'-H), 5.10 (1H, d, *J* = 3.8 Hz, 1"-H), 4.25 (1H, dd, *J* = 10.9, 3.8 Hz, 2"-H), 4.23 - 4.18 (1H, m, 5'-H), 4.02 (1H, d, *J* = 12.8 Hz, 5"-H_a), 3.96 (1H, t, *J* = 9.5 Hz, 4/6-H), 3.81 (1H, t, *J* = 9.5 Hz, 5-H),

3.76 (1H, t, *J* = 9.5 Hz, 4/6-H), 3.63 - 3.52 (4H, m, 5"-H_b, 1-H, 3-H, 2'-H), 3.50 (1H, d, *J* = 10.9, 3"-H), 3.34 - 3.30 (3H, m, 6'-H_a, Ar 1-H_2), 3.22 (1H, dd, *J* = 13.3, 7.4 Hz, 6'-H_b), 3.01 - 2.97 (2H, m, Ar 2-H_2), 2.94 (3H, s, 3"-*N*-CH_3), 2.56 (1H, dt, *J* = 12.5, 4.3 Hz, 2-H_{eq}), 2.06 - 2.00 (2H, m, 3'/4'-H), 1.99 - 1.90 (2H, m, 3'/4'-H, 2-H_{ax}) 1.68 - 1.59 (1H, m, 4'-H), 1.37 (3H, s, 4"-CH_3); **^{13}C NMR** (150 MHz; D_2O) δ 155.3, 130.8, 128.7, 116.4, 102.0, 95.9, 84.5, 77.6, 75.0, 70.5, 68.5, 66.9, 66.2, 63.9, 59.9, 51.1, 50.2, 50.1, 49.3, 49.0, 35.1, 31.1, 28.5, 26.3, 21.5, 21.0; ***m/z*** [MS ES+] R(*t*): 11.0 min, 570 $[M + H]^+$, 592 $[M + Na]^+$; [MS/MS ES+ of *m/z* 570] 553, 411, 322, 249, 160; [HRMS ES+] Found $[M + H]^+$ 570.3503; $C_{27}H_{47}N_5O_8$ requires 570.3497.

6'-*N*-(3,4-dihydroxyphenethyl)-gentamicin C1a (4i)

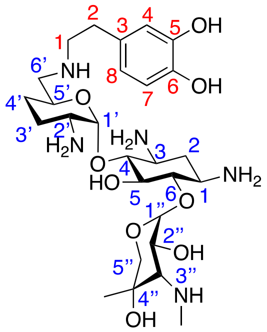

25 equivalents of dopamine hydrochloride (**3i**) were added to the reaction and the reductive amination was incubated for 48 hrs. MeOH was used to remove excess amine instead of EtOAc. The product was purified by tetraphenylborate precipitation and semi-preparative HPLC to give **4i** as a white crystalline solid. Isolated yield: 14%. **^{1}H NMR** (600 MHz; D_2O) δ 6.91 (1H, d, *J* = 8.2 Hz, Ar 7-H), 6.85 (1H, d, *J* = 2.1 Hz, Ar 4-H), 6.77 (1H, dd, *J* = 8.2, 2.1 Hz, Ar 8-H), 5.79 (1H, d, *J* = 3.6 Hz, 1'-H), 5.10 (1H, d, *J* = 3.8 Hz, 1"-H), 4.25 (1H, dd, *J* = 10.9, 3.8 Hz, 2"-H), 4.23 - 4.17 (1H, m, 5'-H), 4.02 (1H, d, *J* = 12.8 Hz, 5"-H_a), 3.97 (1H, t, *J* = 9.5 Hz, 4/6-H), 3.81 (1H, t, *J* = 9.5 Hz, 5-H), 3.76 (1H, t, *J* = 9.5 Hz, 4/6-H), 3.63 - 3.52 (4H, m, 5"-H_b, 1-H, 3-H, 2'-H), 3.50 (1H, d, *J* = 10.9, 3"-H), 3.34 - 3.28 (3H, m, 6'-H_a, Ar 1-H_2), 3.21 (1H, dd, *J* = 13.3, 7.4 Hz, 6'-H_b), 2.97 - 2.91 (5H, m, 3"-*N*-CH_3, Ar 2-H_2), 2.56 (1H, dt, *J* = 12.5, 4.3 Hz, 2-H_{eq}), 2.08 - 1.99 (2H, m, 3'/4'-H), 1.99 - 1.90 (2H, m, 3'/4'-H, 2-H_{ax}) 1.68 - 1.59 (1H, m, 4'-H), 1.37 (3H, s, 4"-CH_3); **^{13}C NMR** (150 MHz; D_2O) δ 144.9, 143.7, 129.5, 121.7, 117.2, 117.1, 102.0, 96.0, 84.5, 77.7, 75.0, 70.5, 68.5, 66.8, 66.2, 63.9, 51.1, 50.2, 50.0, 49.3, 48.9, 35.0, 31.2, 28.5, 26.3, 21.5, 20.9; ***m/z*** [MS ES+] R(*t*): 10.3 min, 586 $[M + H]^+$, 608 $[M + Na]^+$; [MS/MS ES+ of *m/z* 586] 569, 427, 322, 265, 160; [HRMS ES+] Found $[M + H]^+$ 586.3446; $C_{27}H_{47}N_5O_9$ requires 586.34465.

6'-*N*-(Furfuryl)-gentamicin C1a (4j)

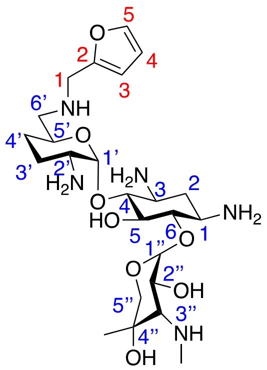

15 equivalents of furfurylamine (**3j**) were added to the reaction and the reductive amination was incubated for 25 hrs. The product was purified by tetraphenylborate precipitation and 2 rounds of semi-preparative HPLC to give **4j** as a white to off-white crystalline solid. Isolated yield: 14% (approximately 85% pure by proton NMR). **^{1}H NMR** (600 MHz; D_2O) δ 7.63 (1H, d, *J* = 1.9 Hz, Ar 5-H), 6.67 (1H, d, *J* = 3.3 Hz, Ar 3-H), 6.53 (1H, dd, *J* = 3.3, 1.9 Hz, Ar 4-H), 5.82 (1H, d, *J* = 3.6 Hz, 1'-H), 5.11 (1H, d, *J* = 3.8 Hz, 1"-H), 4.42 - 4.32 (2H, m, Ar 1-H_2), 4.25 (1H, dd, *J* = 10.9, 3.8 Hz, 2"-H), 4.23 - 4.18 (1H, m, 5'-H), 4.03 (1H, d, *J* = 12.8 Hz, 5"-H_a), 3.99 (1H, t, *J* = 9.5 Hz, 4/6-H), 3.84 (1H, t, *J* = 9.5 Hz, 5-H), 3.79 (1H, t, *J* = 9.5 Hz, 4/6-H), 3.65 - 3.55 (3H, m, 1-H, 3-H, 2'-H), 3.55 - 3.51 (2H, m, 5"-H_b, 3"-H), 3.32 (1H, dd, *J* = 13.2, 3.3 Hz, 6'-H_a), 3.19 (1H, dd, *J* = 13.2, 7.6 Hz, 6'-H_b), 2.94 (3H, s, 3"-*N*-CH_3), 2.56 (1H, dt, *J* = 12.6, 4.3 Hz, 2-H_{eq}), 2.08 - 2.01 (2H, m, 3'/4'-H), 2.01 - 1.91 (2H, m, 3'/4'-H, 2-H_{ax}) 1.68 - 1.57 (1H, m, 4'-H), 1.37 (3H, s, 4"-CH_3); **^{13}C NMR** (150 MHz; D_2O) δ 145.6, 144.6, 113.9, 111.7, 102.0, 95.9, 84.4, 77.5, 75.0, 70.5, 68.5, 66.8, 66.2, 63.9, 50.3, 50.2, 49.3, 48.9, 44.2, 35.0, 28.5, 26.3, 21.5, 21.0; ***m/z*** [MS ES+] R(*t*): 10.8 min, 530 $[M + H]^+$, 552 $[M + Na]^+$; [MS/MS ES+ of *m/z* 530] 513, 371, 322, 209, 160; [HRMS ES+] Found $[M + H]^+$ 530.3186; $C_{24}H_{43}N_5O_8$ requires 530.3184.

6'-*N*-(Cyclopropyl)-gentamicin C1a (4k)

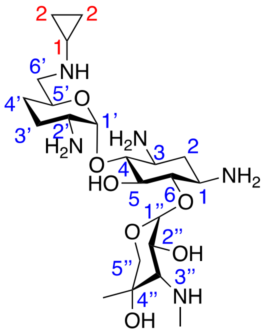

20 equivalents of cyclopropylamine (**3k**) were added to the reaction and the reductive amination was incubated for 48 hrs. The solvent was separated from the beads and the volume reduced *in vacuo*. The remaining residue was washed 3 times with 1 mL EtOAc, and then purified by tetraphenylborate precipitation. The residue obtained was dissolved in 500 µL of H_2O and loaded onto a column of Amberlite CG-50 (NH_4^+) (3 mL) and the product purified using a gradient of 0.1 - 1 M NH_4OH. Product containing fractions were combined and freeze dried. This product was dissolved in minimal H_2O and loaded onto a column of Dowex 1 x 2 (OH^- form) resin (9 mL), the product was eluted with H_2O. Pure product containing fractions were combined to give **4k** as a white powder. Isolated yield: 16% (approximately 80% pure by proton NMR). **^{1}H NMR** (600 MHz; D_2O) δ 5.82 (1H, d, *J* = 3.6 Hz, 1'-H), 5.11 (1H, d, *J* = 3.8 Hz, 1"-H), 4.25 (1H, dd, *J* = 10.9, 3.8 Hz, 2"-H), 4.23 - 4.18 (1H, m, 5'-H), 4.04 (1H, d, *J* = 12.8 Hz, 5"-H_a), 4.00 (1H, t, *J* = 9.4 Hz, 4/6-H), 3.85 (1H, t, *J* = 9.4 Hz, 5-H), 3.80 (1H, t, *J* = 9.4 Hz, 4/6-H), 3.65 - 3.55 (4H, m, 1-H, 3-H, 2'-H, impurity peaks), 3.55 - 3.51 (m, 2H, 5"-H_b, 3"-H), 3.44 (1H, dd, *J* = 13.3, 3.4 Hz, 6'-H_a), 3.33 (1H, dd, *J* = 13.3, 7.6 Hz, 6'-H_b), 2.94 (3H, s, 3"-*N*-CH_3), 2.82 - 2.77 (1H, m, 1-H), 2.57 (1H, dt, *J* = 12.6, 4.3 Hz, 2-H_{eq}), 2.10 - 2.01 (2H, m, 3'/4'-H), 2.01 - 1.94 (2H, m, 3'/4'-H, 2-H_{ax}), 1.68 - 1.59 (1H, m, 4'-H), 1.37 (3H, s, 4"-CH_3), 0.96 - 0.91 (m, 4H, 2-H_4); **^{13}C NMR** (150 MHz; D_2O) δ 102.0, 95.8, 84.4, 77.5, 75.0, 70.5, 68.6, 66.9, 66.1, 63.9, 51.9, 50.2, 49.3, 49.0, 35.1, 31.6, 28.5, 26.3, 21.5, 21.0, 3.6, 3.4; ***m/z*** [MS ES+] R(*t*): 9.2 min, 490 $[M + H]^+$, 512 $[M + Na]^+$; [MS/MS ES+ of *m/z* 490] 473, 331, 322, 169, 160; [HRMS ES+] Found $[M + H]^+$ 490.3238; $C_{22}H_{43}N_5O_7$ requires 490.3235.

2. Supplementary Discussion

The *E. coli* ATCC 25922 host strain appeared to have 4 naturally occurring plasmids, as six bands were observed at 1000 - 1500, 2000, 3000, 4000 and two above 10,000 bp in the undigested lane of the control strain (Figure S10). The top bands (orange asterisk) could be open circle (OC) and super-coiled (SC) forms of a medium copy number plasmid, while the faint bands around 3000 - 4000 could be OC and SC forms of a plasmid which is present at a low copy number per cell. The two other plasmids appear to have higher copy numbers. Upon digestion with *Hind*III, the same bands were observed apart from a shift of the band at ~ 2000 bp to ~ 3000 bp (suggesting that the band at 2000 bp is the SC form of a low molecular weight plasmid), disappearance of the top band above 10,000 bp and two additional faint bands at 2000 and 2500 bp (Figure S10). For the *Not*I digest of *E. coli* ATCC 25922 plasmids, no difference was observed compared to the undigested plasmid lane (Figure S10). The genome of *E. coli* ATCC 25922 has been sequenced and it was shown that the organism contains two plasmids, one at 48 kb and another at 24 kb[9]. The 48 kb plasmid is not cut by *Hind*III and is linearised by *Not*I. Conversely, the 24 kb plasmid is not cut by *Not*I but is cut by *Hind*III at 3 sites, producing bands of 20.9 kb, 2.6 kb and 0.6 kb in length. The bands observed above 10000 bp and ~ 2500 bp in the *Hind*III digest may correspond to the 24 kb plasmid. The band at ~ 3000 bp may be the linearised form of the small plasmid which is observed around 2000 bp. Similar band patterns were observed for all other lanes for the pSa and R26 clones (Figure S10). In contrast to pQR1865, the low copy number of pSa and R26 made it difficult to determine if the plasmids were present by gel electrophoresis analysis alone.

3. Supplementary Tables

Gene Name	Organism	Accession Number	Reference
neoB	*Streptomyces fradiae* DSM 40063	AJ629247.1	10
ribB	*Streptomyces ribosidificus* NRRL B-11466	AJ744850.1	10
livB	*Streptomyces lividus* ATCC 21178	AJ748832.1	10
parB	*Streptomyces rimosus subsp. paromomycinus* NRRL 2455	AJ628955.1	10
btrB	*Bacillus circulans* SANK 72073	AB097196.1	11
pchB	*Paenibacillus chitinolyticus* DSM 11030	BBJT01000002.1	12
kanB	*Streptomyces kanamyceticus* DSM 40500	AJ628422.1	10
tobB	*Streptoalloteichus tenebrarius* DSM 40477T	AJ810851.1	10
forB	*Micromonospora olivasterospora* DSM 43868	AJ628421.1	10
istB	*Streptomyces tenjimariensis* ATCC 31603	AJ845083.1	10
sis5	*Micromonospora inyonensis* TS 388	FJ160413.1	13
genB1	*Micromonospora echinospora* DSM 43036	AJ628149.1	10
genB2	*Micromonospora echinospora* DSM 43036	AJ628149.1	10
genB3	*Micromonospora echinospora* DSM 43036	AJ628149.1	10
genB4	*Micromonospora echinospora* DSM 43036	AJ628149.1	10

Table S1: Sources of C-6’ TAm sequence data used in this study.

Oligo Name	Sequence
PchB fw	AAAGCTCTTCGATGAAACAGGATATGGTTGAAAATTCGGAAAAGC
PchB rev	AAAGCTCTTCGGTGACCCCCACCGATCAGATTATCGAG

Table S2: Oligonucleotides for PCR amplification of PchB.

Compound	KOH concentration gradient	R_t (min)
**1a**	0.85 mM KOH, 0 – 5 mins, 0.85 – 1.75 mM KOH, 5 – 5.5 mins, 1.75 mM KOH, 5.5 – 10 mins	9.2
**1b**	0.85 mM KOH, 0 – 5 mins, 0.85 – 1.5 mM KOH, 5 – 5.5 mins, 1.5 mM KOH, 5.5 – 10 mins	8.7
**1c**	0.85 mM KOH, 0 – 5 mins, 0.85 – 1.75 mM KOH, 5 – 5.5 mins, 1.75 mM KOH, 5.5 – 10 mins	8.3
**1d**	1 mM KOH, 0 – 5 mins, 1-2 mM KOH, 5-5.5 mins, 2 mM KOH, 5.5 – 10 mins	9.0
**1e**	1 mM KOH, 0 – 5 mins, 1 – 3 mM KOH, 5 – 5.5 mins, 3 mM KOH, 5.5 – 10 mins	12.7
**1e** (M2)[a]	3 mM KOH isocratic for 20 mins	8.4
**2e**	3 mM KOH isocratic for 20 mins	2.7
**1f**	0.85 mM KOH, 0 – 5 mins, 0.85 – 1.5 mM KOH, 5 – 5.5 mins, 1.5 mM KOH, 5.5 – 10 mins	13.0
**1f** (M2)[a]	2 mM KOH, 0 – 5 mins, 2 – 4 mM KOH, 5 – 5.5 mins, 4 mM KOH, 5.5 – 13 mins, 4 – 2 mM KOH, 13 – 13.5 mins, 2 mM KOH, 13.5 – 16 mins	8.5
**1g**	0.85 mM KOH, 0 – 5 mins, 0.85 – 1.75 mM KOH, 5 – 5.5 mins, 1.75 mM KOH, 5.5 – 10 mins	9.4
**1h**	1 mM KOH, 0 – 5 mins, 1-3 mM KOH, 5 – 5.5 mins, 3 mM KOH, 5.5 – 10 mins	9.6

Table S3: Compound-specific conditions for HPAE-IPAD analysis. [a]= M2 means method 2. 3 mM isocratic methods used for substrate solubility tests.

Compound	Gradient used	R_t (min)[a]
6’-(4-chlorophenyl)-gentamicin C1a (**4d**)	35 – 50 %	10.5
6’-(benzyl)-gentamicin C1a (**4f**)	12.5 – 32.5 %	12.4
6’-(phenethyl)-gentamicin C1a (**4g**)	20 – 40 %	12.1
6’-(4-hydroxyphenethyl)-gentamicin C1a (**4h**)	15 – 25 %	9.1
6’-(furfuryl)-gentamicin C1a (**4j**)	10 – 15 %	3.9

Table S4: Compound-specific conditions for analytical HPLC. All gradients were run for 16 mins in methanol followed by a 60 % methanol wash step for 3 mins and re-equilibration at the starting mobile phase percentage for 5 mins. [a] = retention times are shown as an average value for the method, however due to the ion-pairing retention mechanism, the retention times of the compounds drifted substantially over the course of the run. R_t = retention time.

Compound	Gradient used	R_t (min)[a]
6’-(phenyl)-gentamicin C1a (**4a**)	15 – 30 %	6.6
6’-(2-chlorophenyl)-gentamicin C1a (**4b**)	25 – 45 %	11.8
6’-(3-chlorophenyl)-gentamicin C1a (**4c**)	25 – 45 %	8.4
6’-(4-chlorophenyl)-gentamicin C1a (**4d**)	25 – 45 %	10.6
6’-(pyridin-3-ylamino)-gentamicin C1a (**4e**)	2.5 – 15 %	11.6
6’-(benzyl)-gentamicin C1a (**4f**)	10 – 35 %	11.6
6’-(phenethyl)-gentamicin C1a[b] (**4g**)	10 – 20 %	10.8
6’-(4-hydroxyphenethyl)-gentamicin C1a (**4h**)	4 – 15 %	13.8
6’-(3,4-dihydroxyphenethyl)-gentamicin C1a (**4i**)	2.5 – 15 %	9.8
6’-(furfuryl)-gentamicin C1a (**4j**)	5 – 15 %	9.7

Table S5: Compound-specific conditions for semi-preparative HPLC. All gradients were run for 16 mins, methods using methanol then had a 60 % wash step for 3 mins and re-equilibration at the starting mobile phase percentage for 12 mins. [a] = retention times are shown as an average value for the method, however due to the ion-pairing retention mechanism, the retention times of the compounds drifted substantially over the course of a preparative run. [b] = **4g** was purified on the analytical HPLC system described prior using an ACE C_{18}-AR (150 x 4.6 mm) column using acetonitrile as an organic mobile phase, the flow rate was 1 mL/min, the gradient was for 16 mins, followed by a 95% acetonitrile wash step for 2 mins and 5 mins of re-equilibration at the starting mobile phase percentage. R_t = retention time.

4. Supplementary Figures

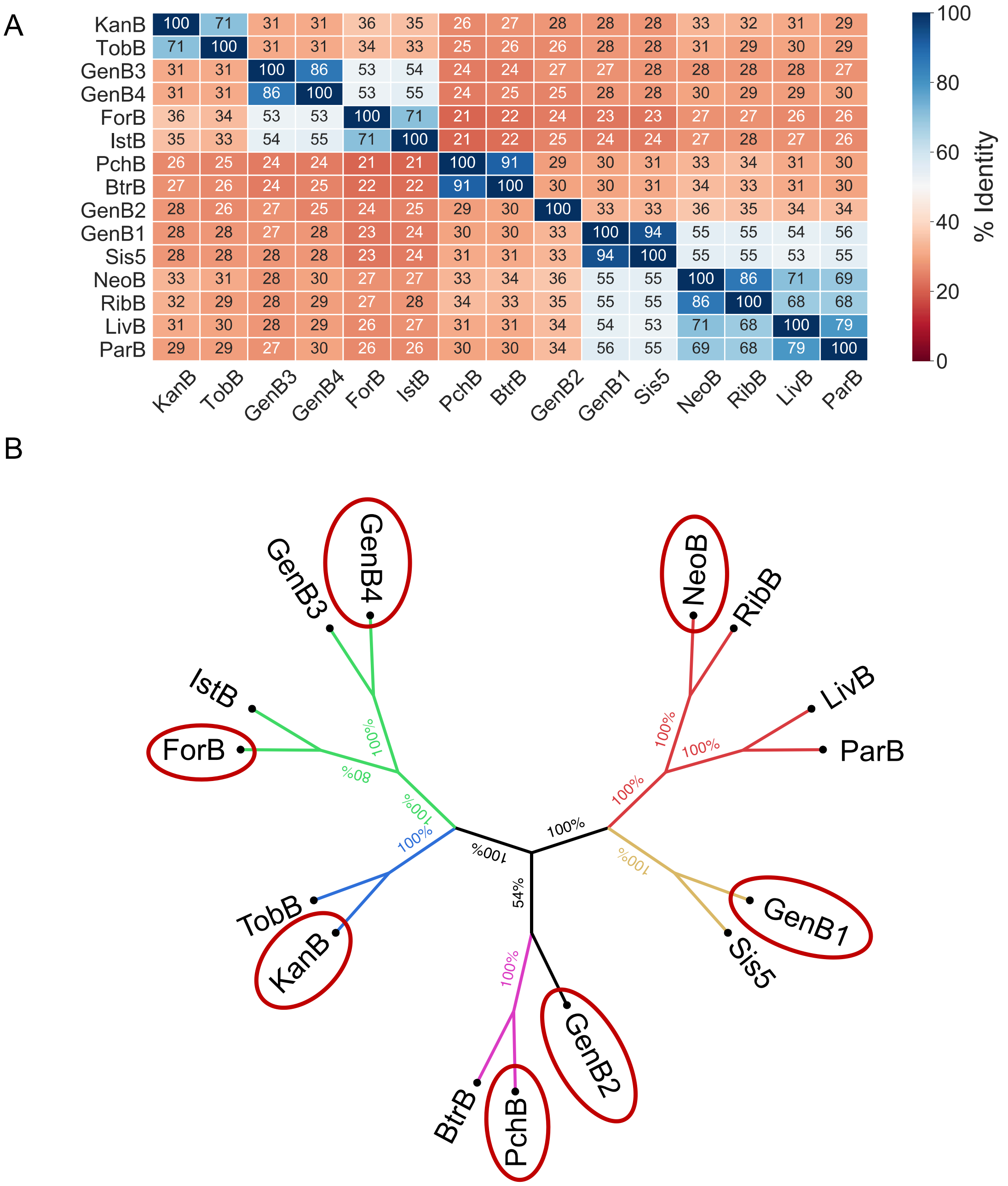

Figure S1: Amino acid sequence analysis of C-6' TAms explored in this study. (A) Heatmap of MSA of C-6' TAms. A percent identity matrix was generated using the ClustalOmega web service, and the figure was visualized in Python 3.6 using the *seaborn* package. (B) Phylogenetic analysis of C-6' TAms. Percentages represent the bootstrap support values for the branches. The high sequence homology groups shown in (A) are coloured to differentiate the groups. Red circles highlight the C-6' TAm enzymes which were selected for further investigation.

A

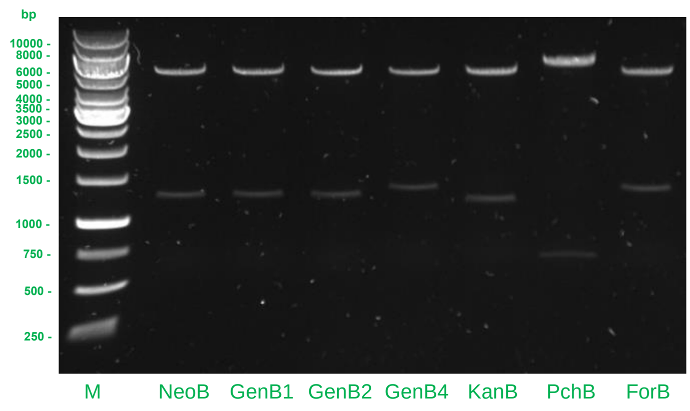

B

Protein	Expected band size (bp)
NeoB	1255
GenB1	1258
GenB2	1249
GenB4	1342
KanB	1183
PchB	605
ForB	1333

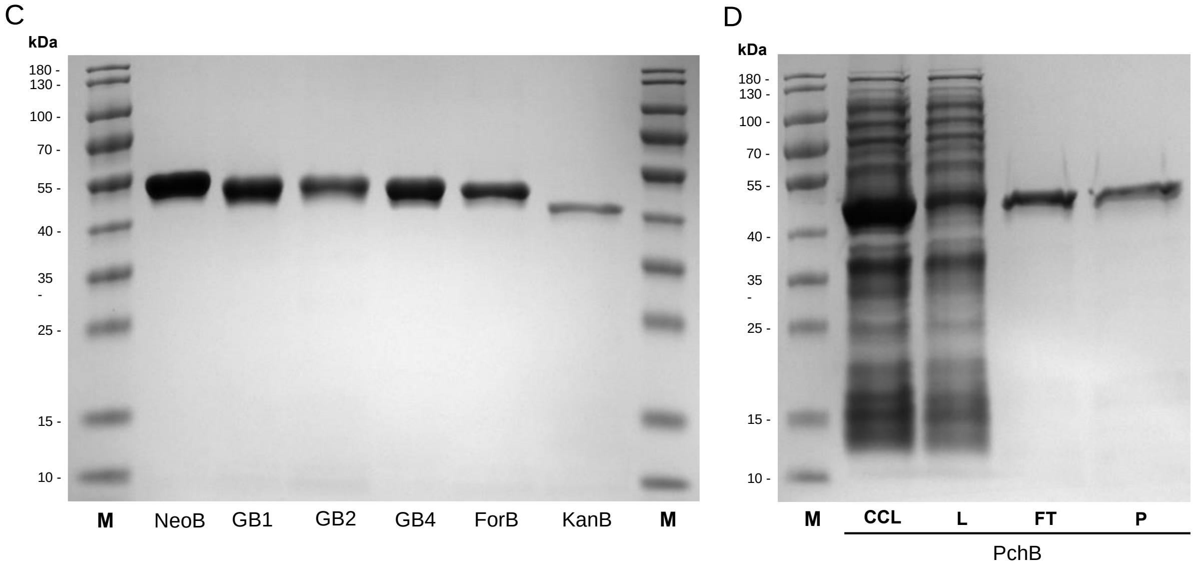

E

Protein	NeoB	GenB1	GenB2	GenB4	ForB	KanB	PchB
MW (kDa)	47	47	47	51	50	45	49

Figure S2: Cloning and Purification of C-6’-TAms. Restriction enzyme digestion analysis of C-6’ TAms cloned into pET28/29 vectors. (A) Agarose gel of the restriction enzyme digests. (B) Table showing the expected fragment sizes for each plasmid. (C) SDS-PAGE analysis of purified C-6’ TAms. (D) SDS-PAGE analysis of purified PchB, CCL = clarified cell lysate, L = eluate obtained during loading of the clarified cell lysate onto the nickel column, FT = 500 mM imidazole elution prior to elution of the PchB protein, P = PchB protein elution with 500 mM imidazole. (E) Table showing the monomer molecular weights of the proteins, calculated using ProtParam.

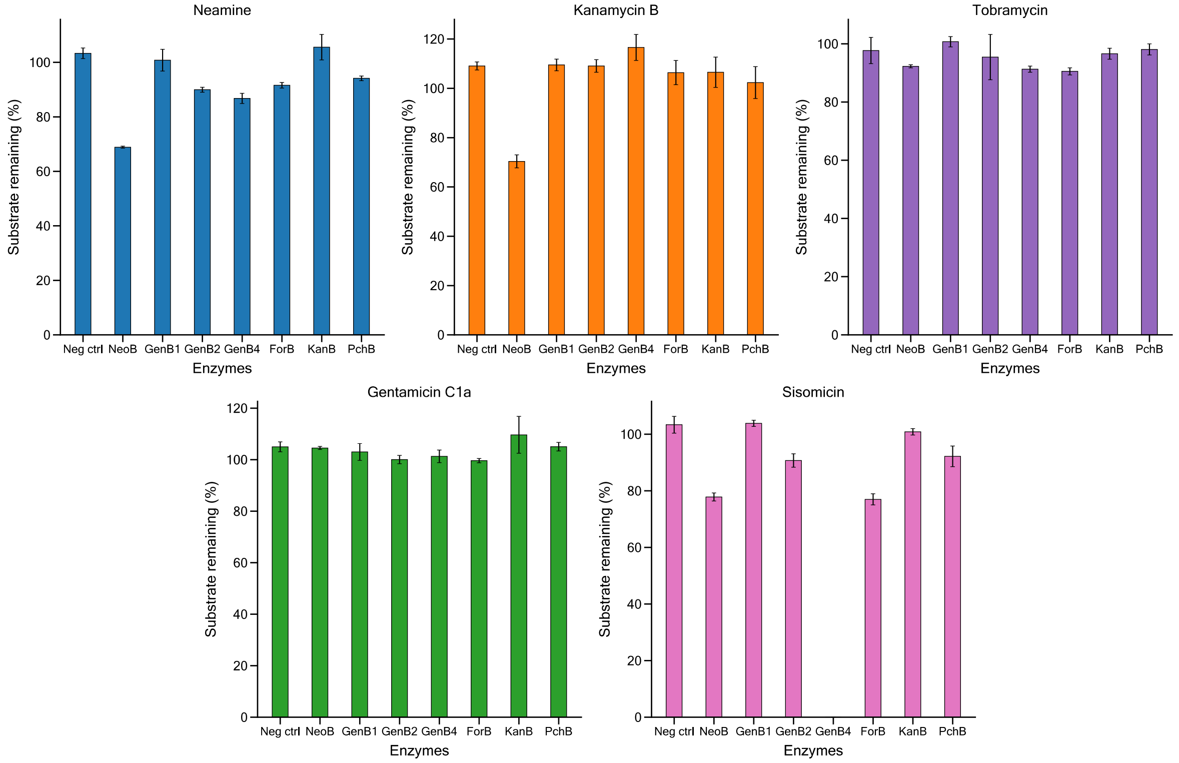

Figure S3: Analysis of substrate depletion for the deamination screening of aminoglycosides by C-6' TAms. All enzymes and substrates shown were tested at least in duplicate and error bars indicate standard deviations (<± 8%).

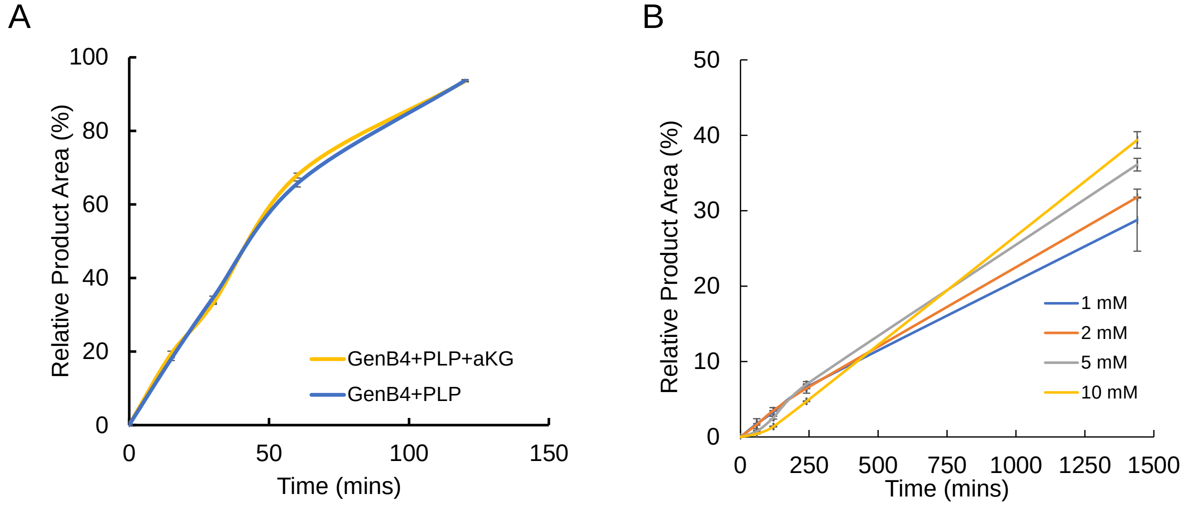

Figure S4: GenB4 reaction optimisation (A) Comparison of the GenB4 reaction in the presence and absence of α-ketoglutarate as an amine acceptor. Enzyme concentration was 0.125 mg/mL, reactions were performed in triplicate and error bars indicate standard deviations (<± 2%). (B) Graph of side product formation from the GenB4 assay at different **1f** concentrations, Reactions were performed in duplicate, means are shown and error bars are standard deviations (<± 4%).

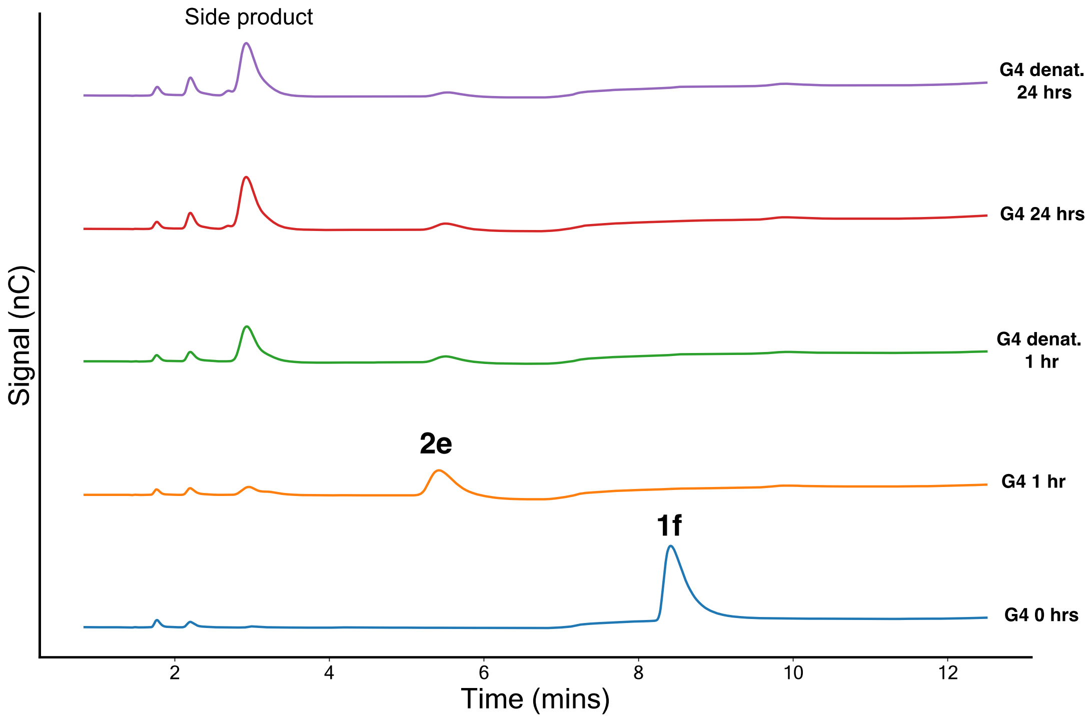

Figure S5: HPAE-IPAD analysis of side product formation in GenB4 reaction with sisomicin. HPAE-IPAD chromatograms are displayed which show the **1f** peak at t_0 is completely converted to **2e** after 1 hr. After 24 hrs, **2e** is completely converted to the side product. Alternatively, incubation for 10 minutes to denature the GenB4 enzyme also leads to side product formation. G4 = GenB4, G4 denat. = GenB4 enzyme which is denatured by heat treatment.

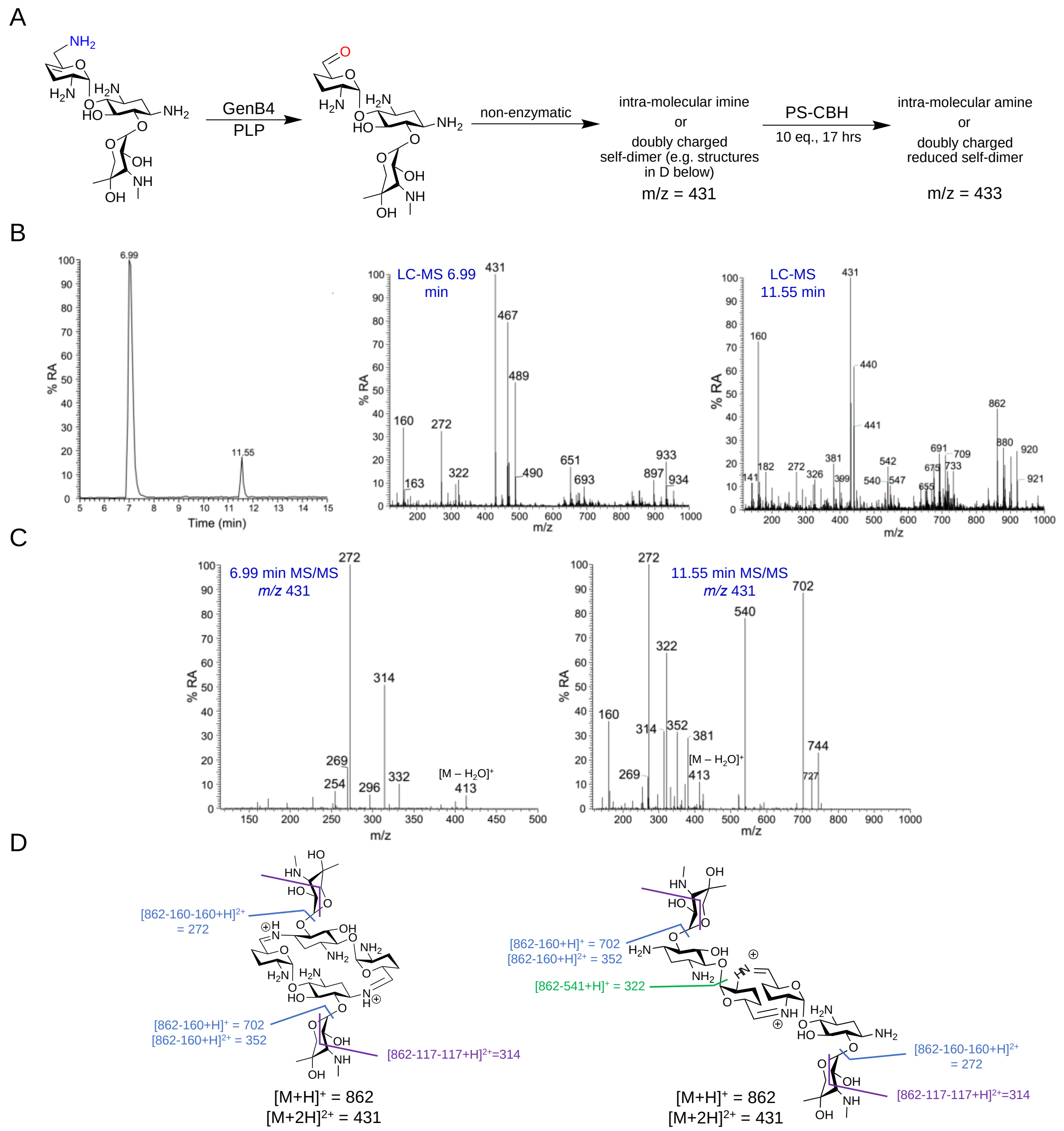

Figure S6: LC-ESI+-MS and LC-ESI+-MS/MS analysis of the side product formed. (A) Scheme showing the reaction analysed. The GenB4 reaction was performed and the enzyme was then incubated for 30 minutes at 95 °C to promote side product formation. This was then reduced in the presence of PS-CBH. (B) LC-ESI+-MS chromatograms of the side products present at 6.99 and 11.55 mins. (C) LC-MS/MS chromatograms of the side products present at 6.99 and 11.55 mins. (D) Possible structures of the doubly charged self-dimer products, suggested mass fragmentations are also shown.

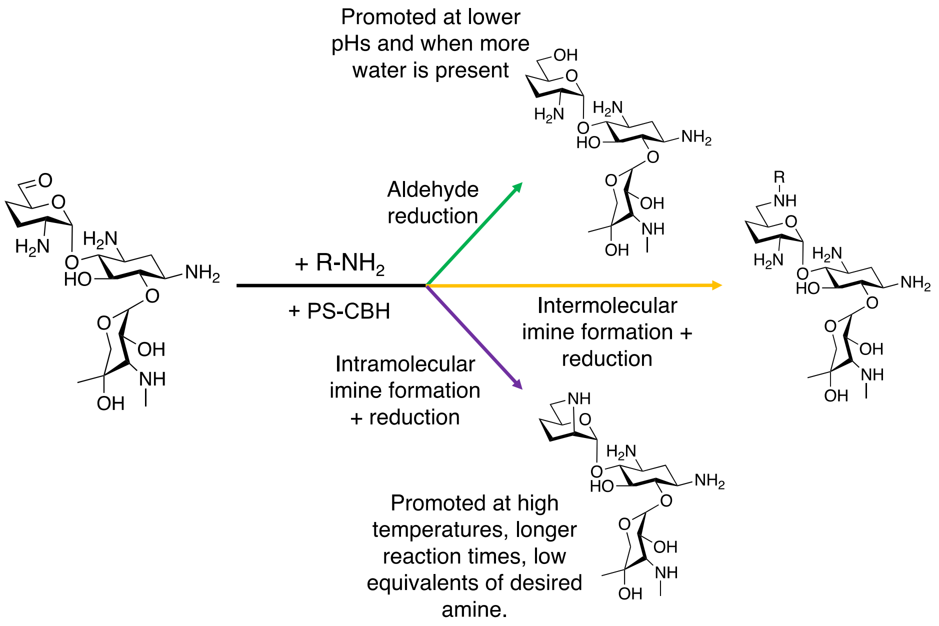

Figure S7: Diagram of the possible reactions that can occur in the reductive amination reaction. Also shown are the conditions which favour the formation of by-products.

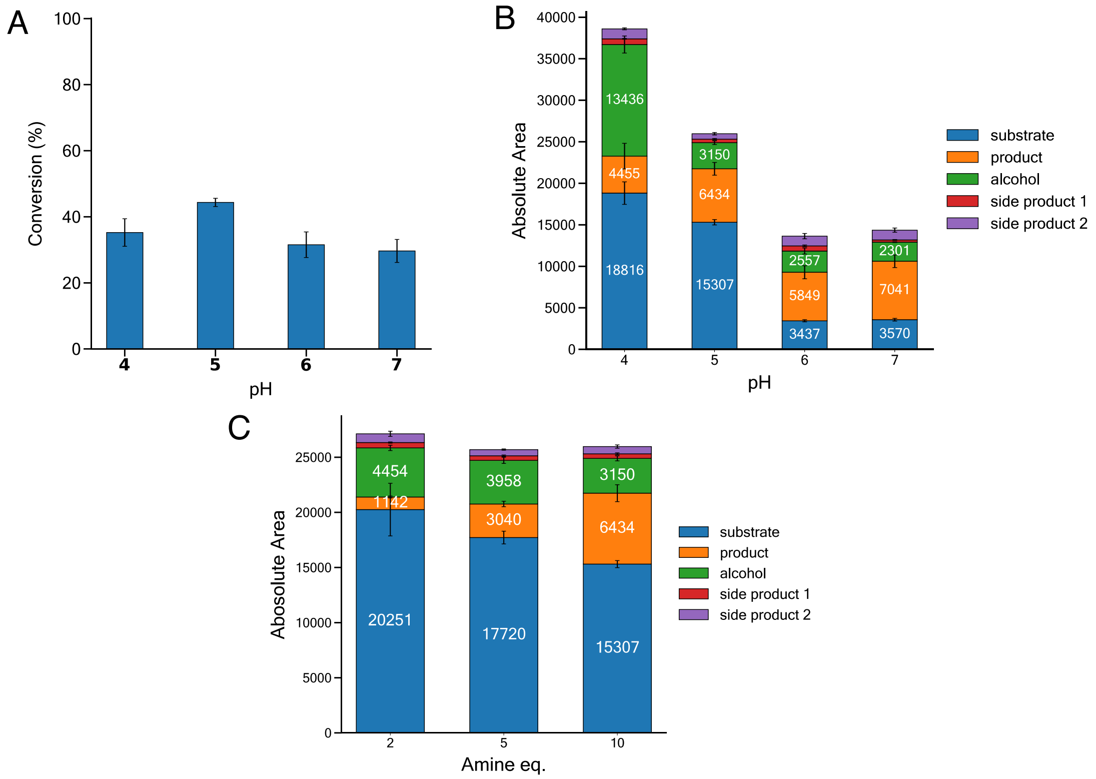

Figure S8: Optimisation of the reductive amination reaction in terms of reaction pH and amine equivalents. (A) Bar plot of the pH optimisation of the reductive amination reaction with **3f**. (%) yield was determined by calibration with **4f** as an external product standard. Bars are the means of triplicate experiments and errors bars represent the standard deviation (< ± 4%). (B) Bar plot of the absolute peak areas for the reaction pH optimisation. Relative peak areas were obtained from the selective ion monitoring chromatograms of the geminal diol of **2e** (substrate, *m/z* 467), 6'-phenethylgentamicin

C1a (**4g**, product, *m/z* 554), 6'-hydroxygentamicin C1a (alcohol, *m/z* 451), intramolecular amine side products (side product 1 and 2, *m/z* 433). Absolute areas are the average of triplicate experiments and error bars represent the standard deviation. (C) Bar plot of amine equivalents optimisation. Reactions were performed at pH 5.0. Areas were obtained as for (C), absolute areas are the average of triplicate experiments, the error is the standard deviation. In both B and C, the bar values for the intramolecular imines were not shown to aid clarity.

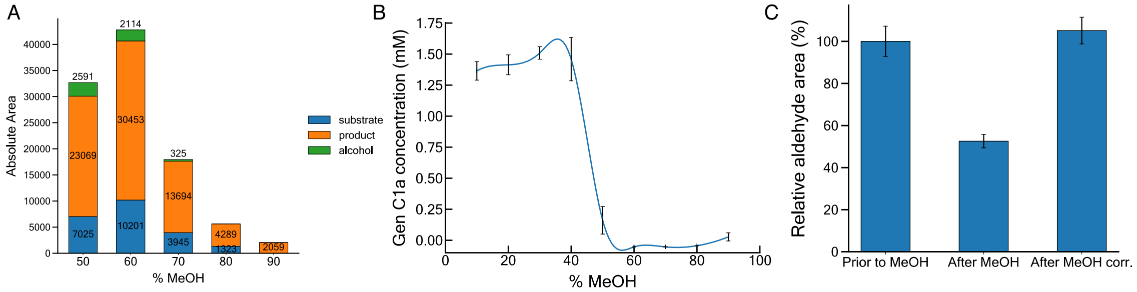

Figure S9: Optimisation of the reductive amination reaction in terms of % methanol added and **2e** solubility. (A) Bar plot of the optimisation of % methanol in the reaction. Absolute peak areas were obtained from the selective ion monitoring chromatograms of the geminal diol of **2e** (substrate, *m/z* 467), **4g** (product, *m/z* 554) and 6'-hydroxygentamicin C1a (alcohol, *m/z* 451). Intramolecular amine areas are not shown but were present at 4 - 9% relative peak area. Absolute area values are shown in black. (B) Plot of gentamicin C1a (**1e**) soluble concentration vs. % methanol concentration in aqueous mixtures. Integrated areas were obtained from HPAE-IPAD analysis of samples which were converted to concentration by calibration with an external standard of authentic gentamicin C1a. Solubility tests were performed in triplicate and the error bars show the standard deviation (< ± 0.2 mM). (C) Bar plot of **2e** solubility before and after the addition of an equal volume of methanol (50% final methanol content in the mixture). Integrated areas were obtained from HPAE-IPAD analysis and then normalised to the area of **2e** prior to the addition of methanol. Bars are the means of triplicate experiments and error bars represent the standard deviation (< ± 7%).

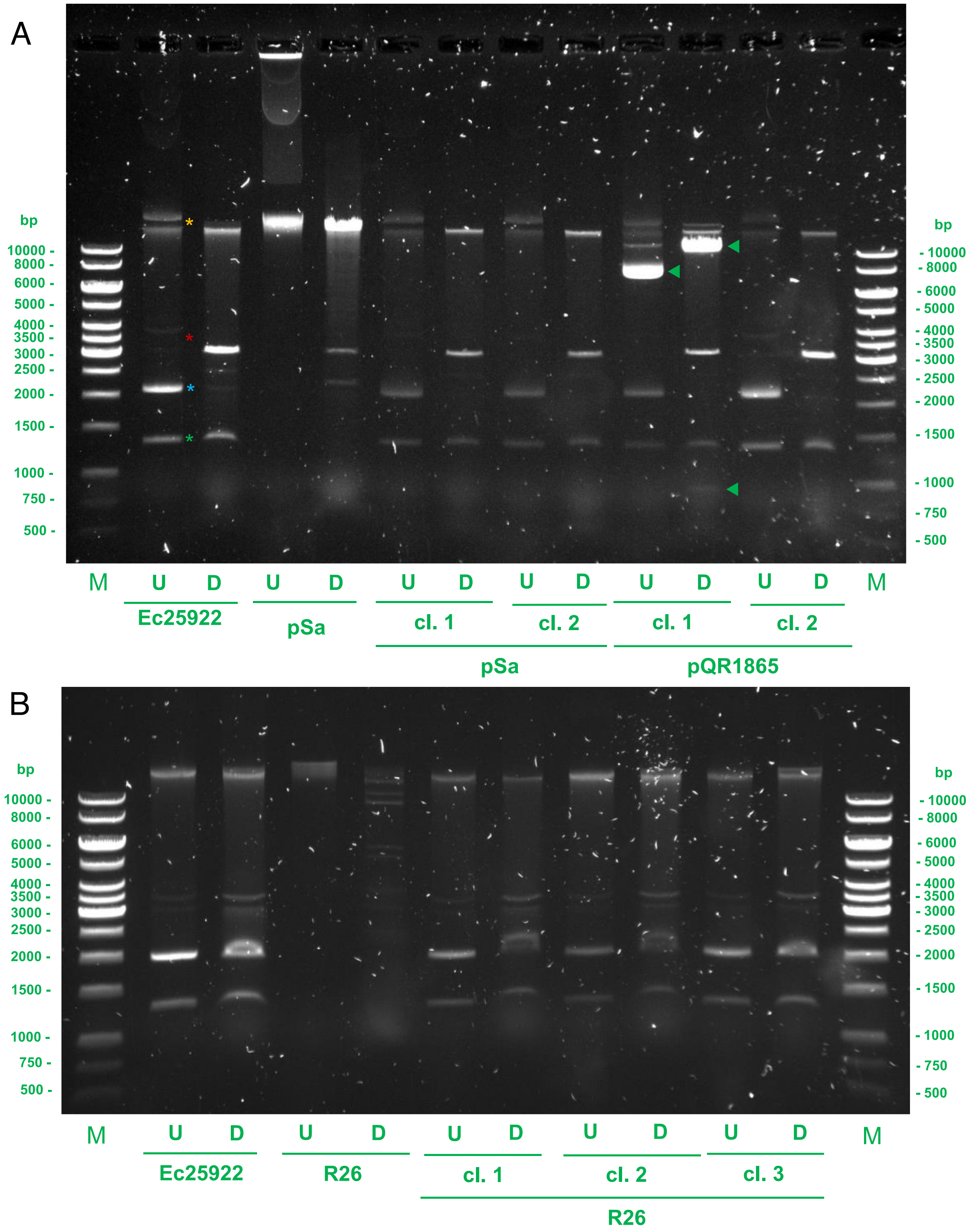

Figure S10: Agarose gel electrophoresis analysis of the restriction enzyme digests of purified plasmids from *E. coli* strains transformed with the aminoglycoside resistance plasmids. (A) Agarose gel electrophoresis analysis of pSa and pQR1865 transformed *E. coli*. Mini-prepped plasmids were digested with *Hind*III for 1 hr, the enzyme was heat-inactivated and then samples were analysed. U = undigested plasmid, D = *Hind*III digested plasmids. Ec25922 = plasmids mini-prepped from the *E. coli* ATCC 25922 host prior to transformation. pSa = pSa plasmid prior to transformation. pSa cl. 1 and cl. 2 = two individual clones of *E. coli* ATCC 25922 transformed with pSa. pQR1865 cl 1. and cl. 2 = two individual clones of *E. coli* ATCC 25922 transformed with pQR1865.

Green arrows indicate bands unique to pQR1865 in the undigested and digested samples. Coloured asterisks denote potential unique plasmids in *E. coli* ATCC 25922. (B) DNA agarose gel electrophoresis analysis of R26 transformed *E. coli*. Mini-prepped plasmids were digested with *Not*I for 1 hr, the enzyme was heat-inactivated and then samples were analysed. U = undigested plasmid, D = *Not*I digested plasmids. Ec25922 = same as for (A), R26 = R26 plasmid prior to transformation. R26 cl. 1, cl. 2 and cl. 3 = three individual clones of *E. coli* ATCC 25922 transformed with R26.

A

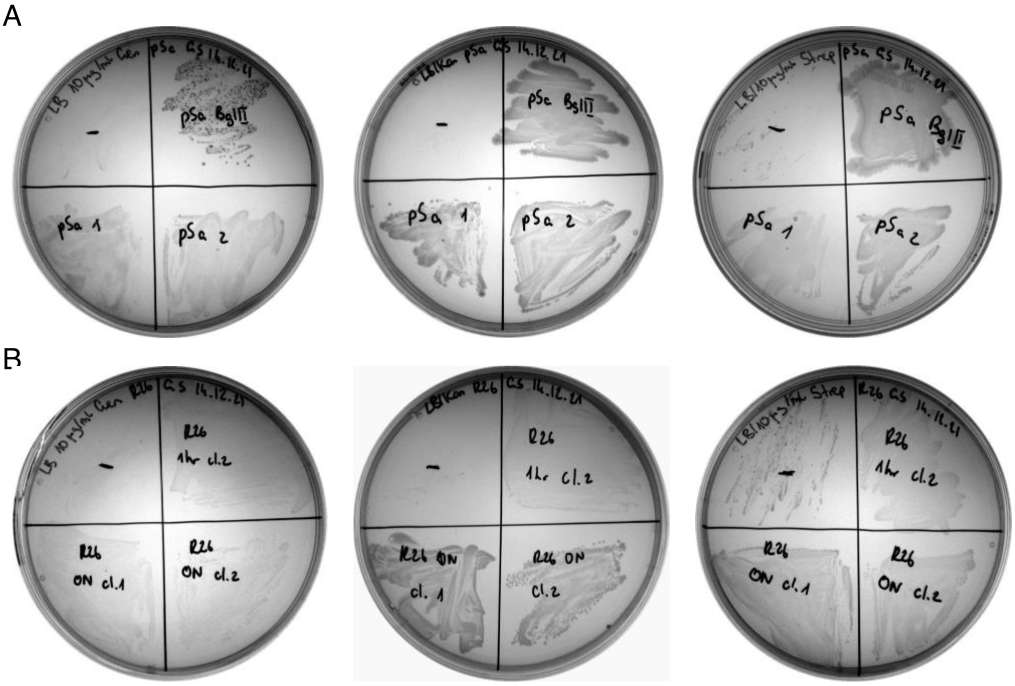

Figure S11: Analysis of the antibiotic resistance phenotype of the pQR1865, pSa and R26 transformed *E. coli* strains. (A) Antibiotic screening of pQR1865 and pSa clones. From left to right - 10 µg/mL gentamicin C1a, 50 µg/mL kanamycin, 10 µg/mL streptomycin. "-" = *E. coli* ATCC 25992 host strain, pSa *Bgl*II = pQR1865. (B) Same as for (A) only for the R26 clones. Mild resistance of the host strain to streptomycin is evident, which is increased when the strain is cloned with the resistance plasmids. All plates contain re-streaks of individual colonies grown on the selection plates after transformation. "1 hr" and "ON" refers the length of the recovery period at 37 °C post transformation, which was either 1 hr or overnight.

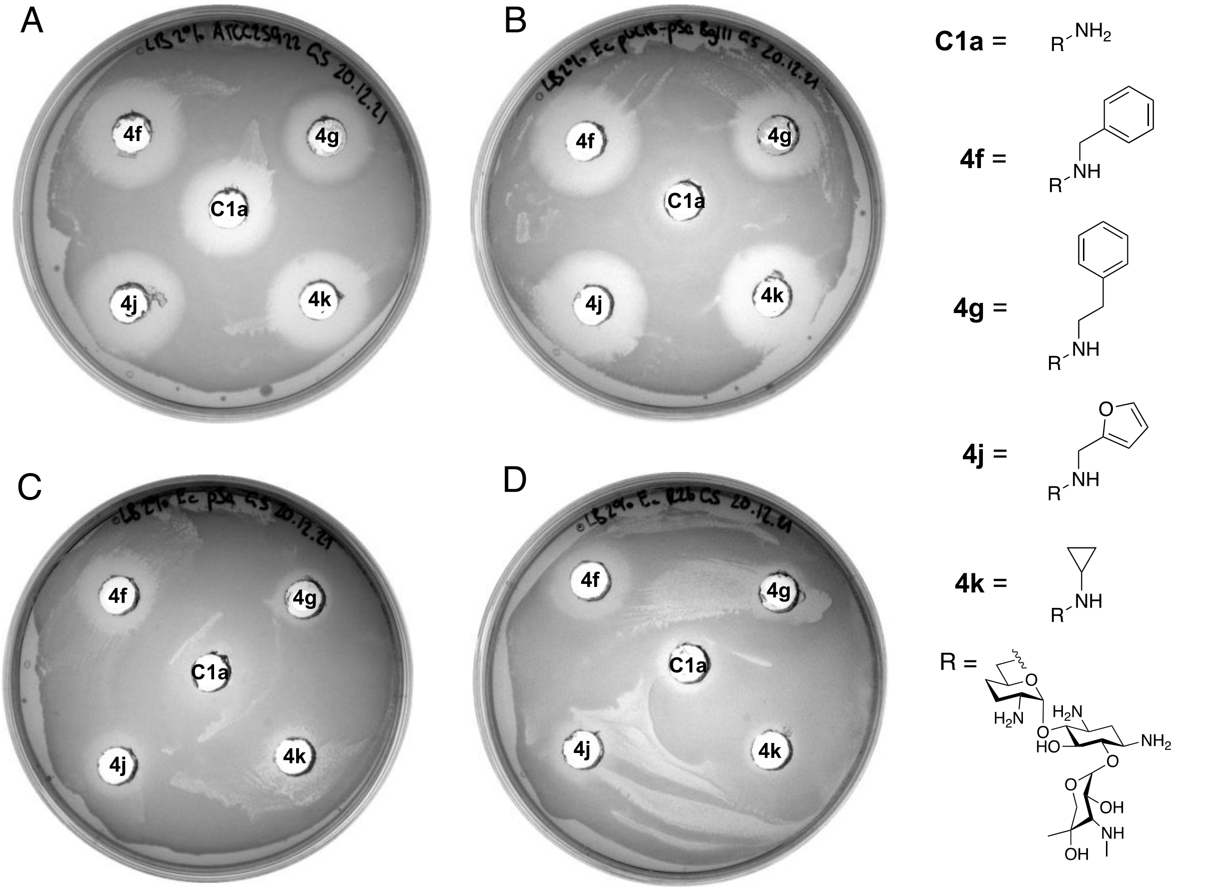

Figure S12: Solid media screening of antimicrobial 6'-gentamicin C1a derivatives against the resistant *E. coli strains*. (A) *E. coli* ATCC 25922 host strain. Compounds were diluted to a concentration of 0.1 mg/mL and 100 µL was added to each well. Plates were incubated overnight at 37 °C prior to imaging. (B) *E. coli* ATCC 25922 + pQR1865, (C) *E. coli* ATCC 25922 + pSa, (D) *E. coli* ATCC 25922 + R26.

5. LC-MS analysis of 6’-oxoaminoglycosides

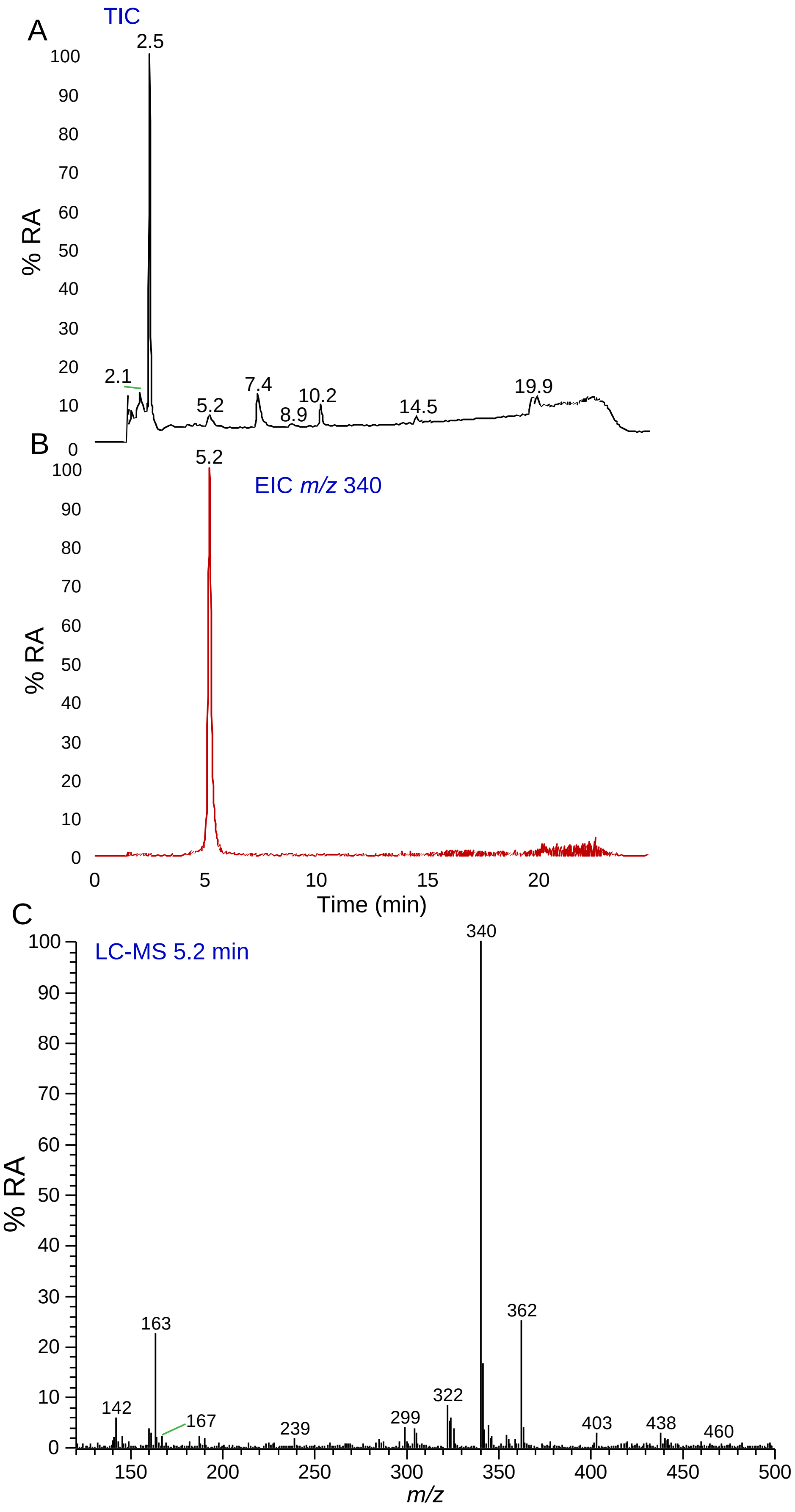

D

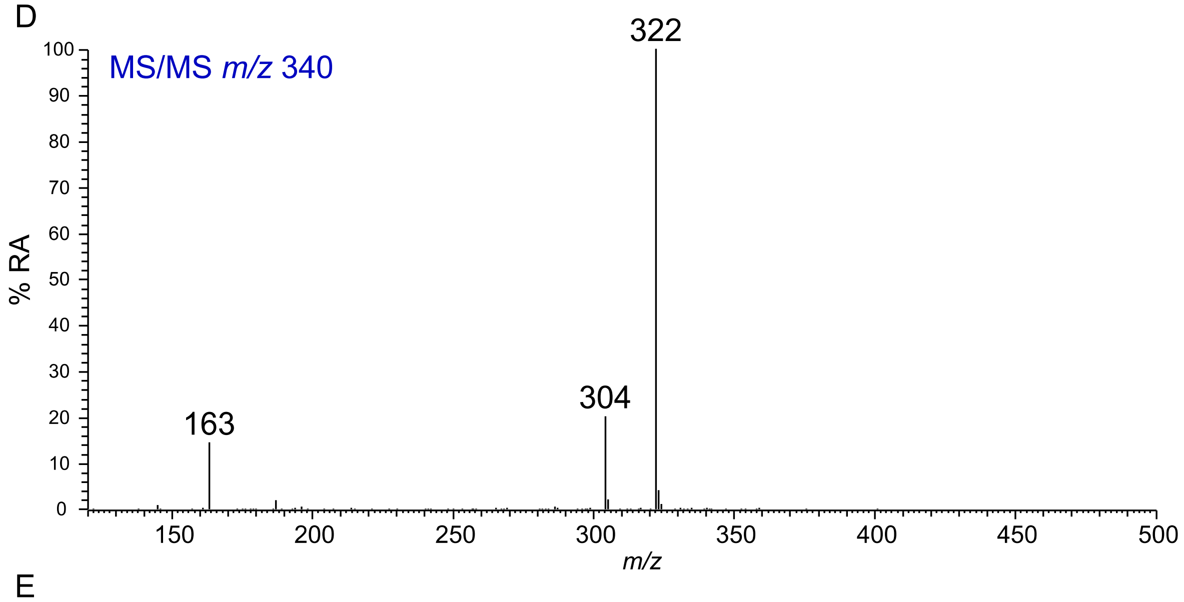

E

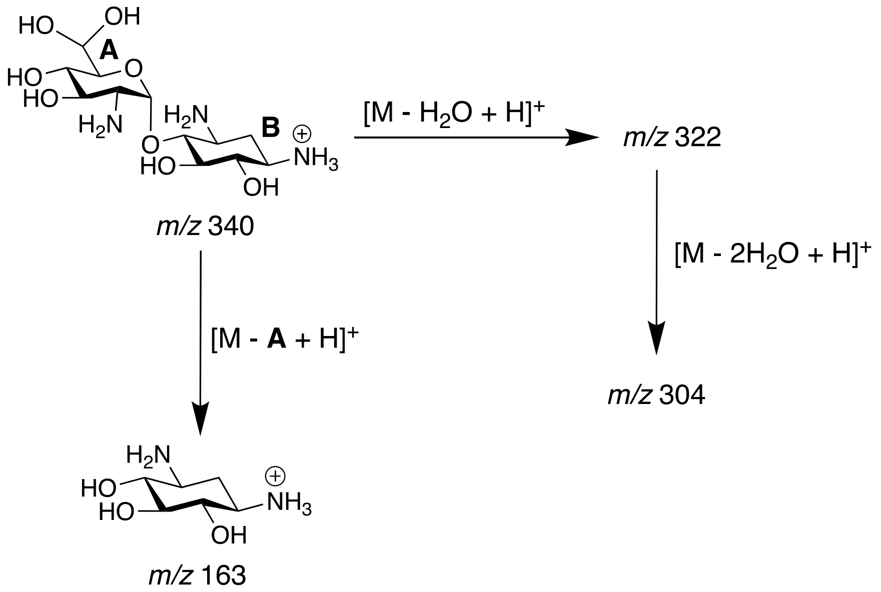

Figure S13: LC-MS and LC-MS/MS analysis of 6’-oxoparomamine obtained from the deamination reaction of neamine with NeoB after 24 hrs. (A) Total ion chromatogram (TIC) of the reaction, (B) Extracted ion chromatogram (EIC) for the mass of the geminal diol of 6’-oxokanamycin B (*m/z* 340), (C) LC-MS analysis of the peak at 5.2 min. (D) LC-MS/MS analysis of the mass at *m/z* 340, (E) Expected fragmentation pattern of the geminal diol of 6’-oxoparomamine.

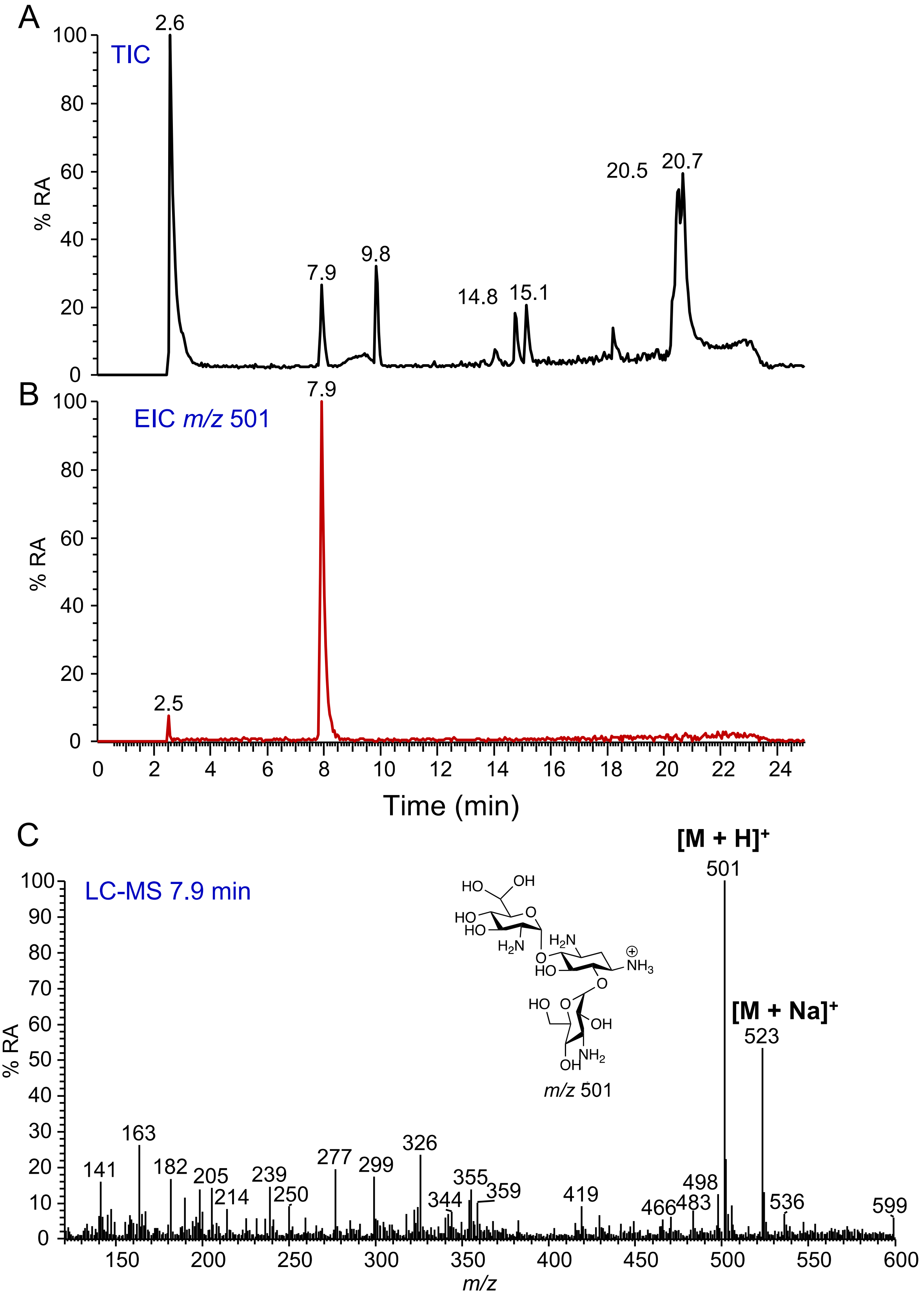
A
TIC
% RA
2.6
7.9
9.8
14.8
15.1
20.5
20.7
B
EIC m/z 501
7.9
2.5
Time (min)
C
LC-MS 7.9 min
[M + H]+
501
[M + Na]+
523
m/z 501
% RA
141
163
182
205
214
239
250
277
299
326
344
355
359
419
466
483
498
536
599
m/z

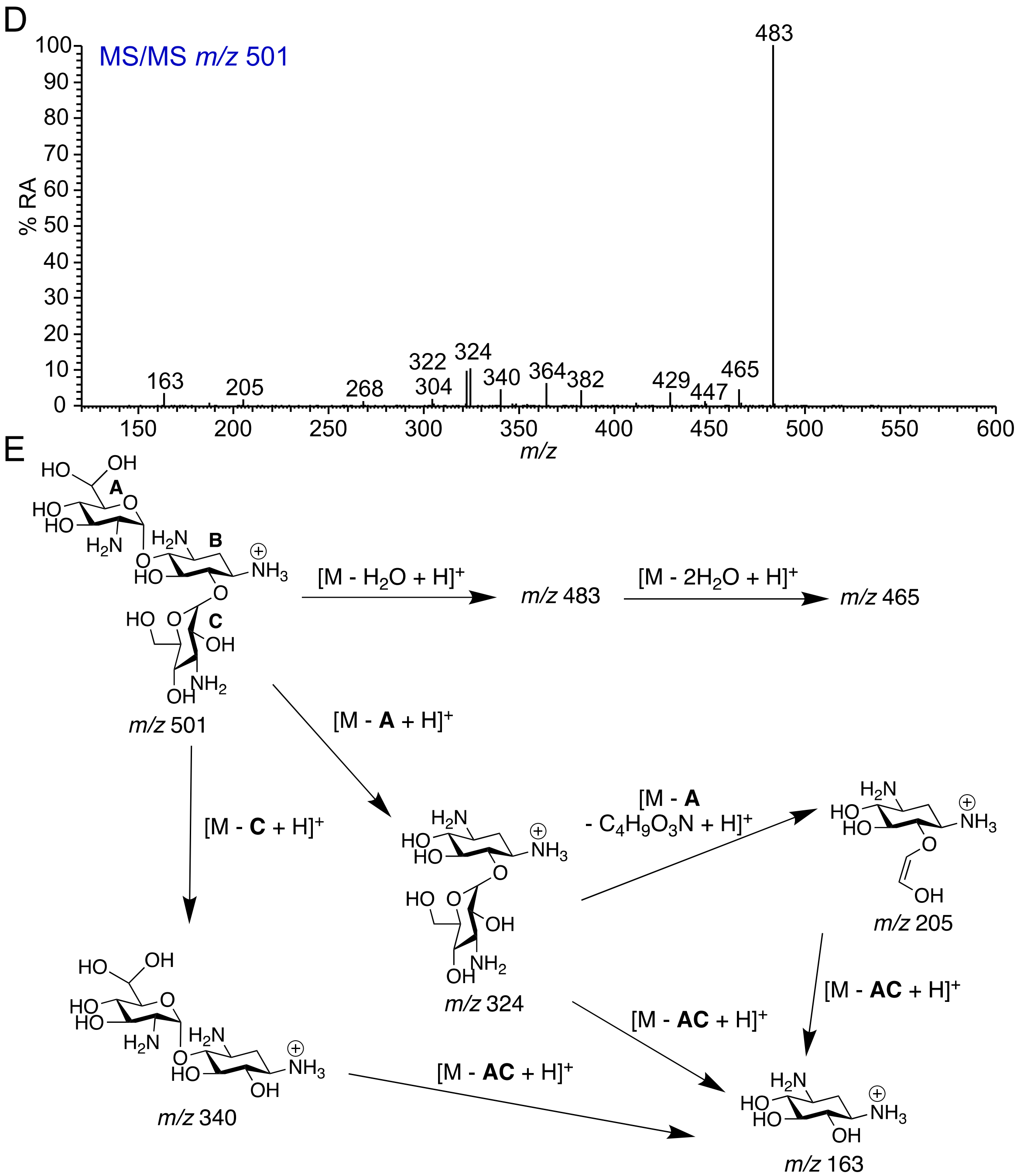

Figure S14: LC-MS and LC-MS/MS analysis of 6'-oxokanamycin B obtained from the deamination reaction of kanamycin B with NeoB after 24 hrs. (A) TIC of the reaction, (B) EIC for the mass of the geminal diol of 6'-oxokanamycin B (*m/z* 501), (C) LC-MS analysis of the peak at 7.9 min. (D) LC-MS/MS analysis of the mass at *m/z* 501, (E) Expected fragmentation pattern of the geminal diol of 6'-oxokanamycin B.

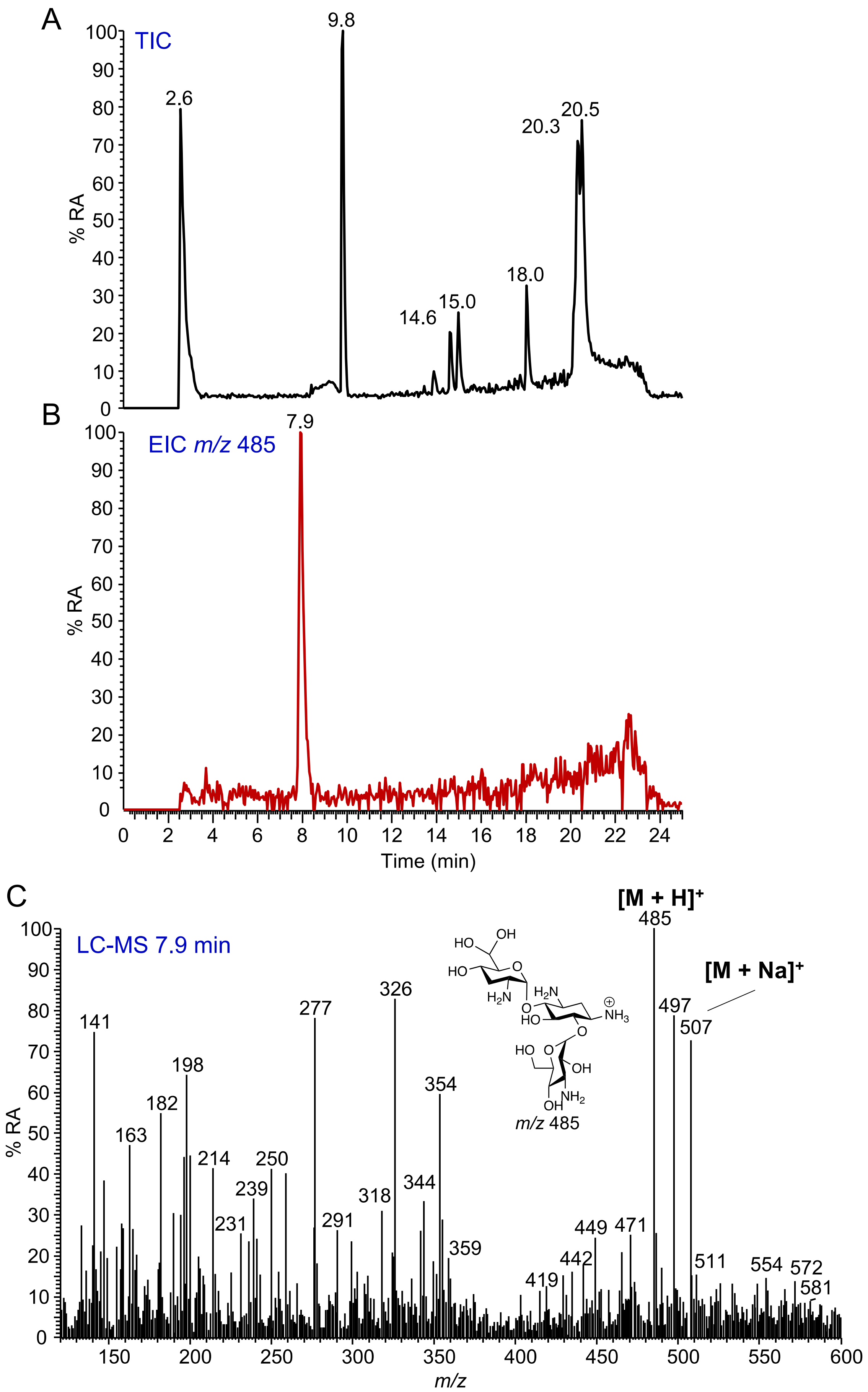
A
TIC
% RA
2.6
9.8
14.6
15.0
18.0
20.3
20.5
B
EIC m/z 485
7.9
Time (min)
C
LC-MS 7.9 min
[M + H]+
485
[M + Na]+
497
507
141
163
182
198
214
231
239
250
277
291
318
326
344
354
359
419
442
449
471
511
554
572
581
m/z 485
m/z

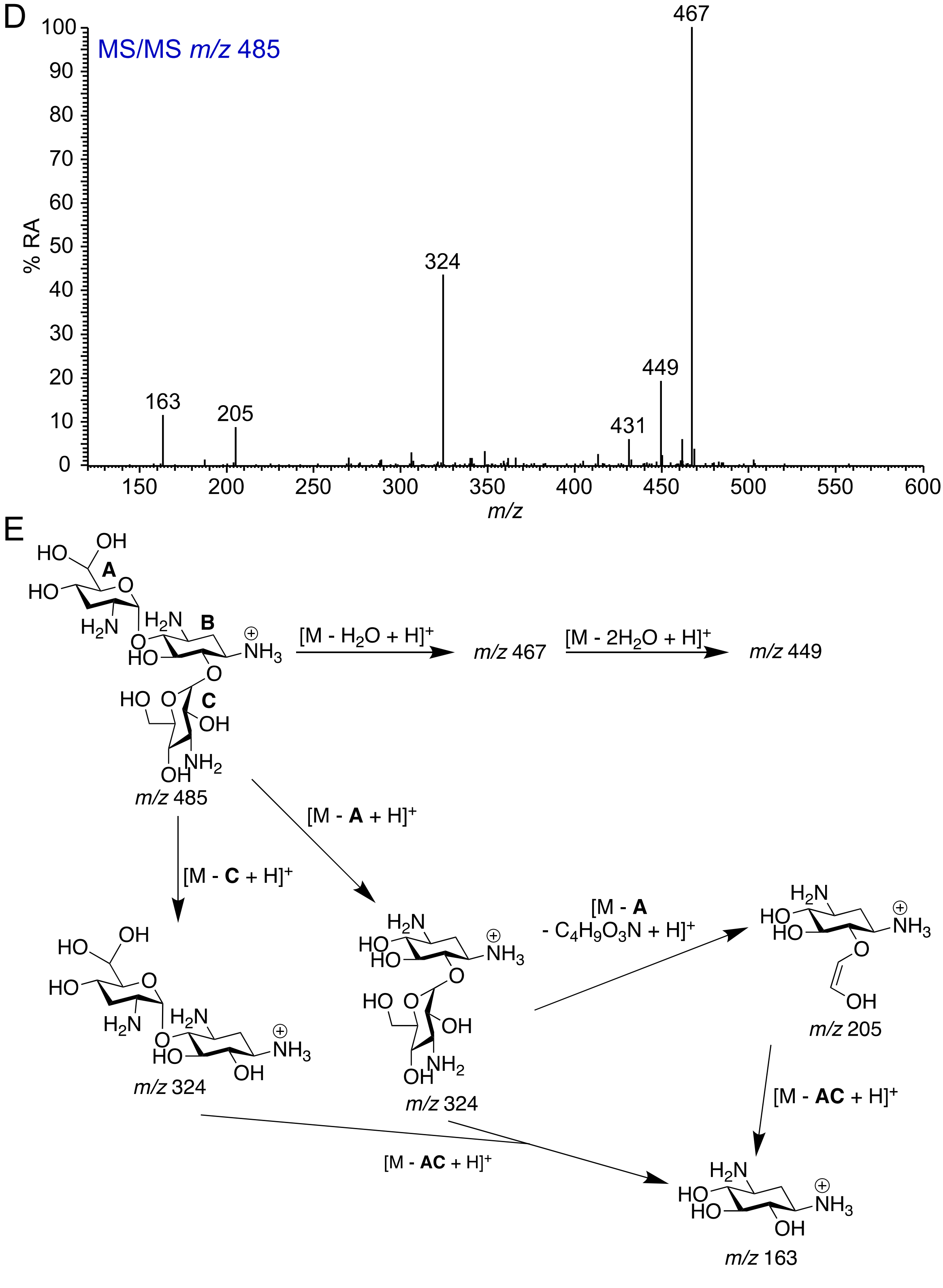

Figure S15: LC-MS and LC-MS/MS analysis of 6'-oxotobramycin obtained from the deamination reaction of tobramycin with GenB2 after 24 hrs. (A) TIC of the reaction, (B) EIC for the mass of the geminal diol of 6'-oxotobramycin (*m/z* 485), (C) LC-MS analysis of the peak at 7.9 min. (D) LC-MS/MS analysis of the mass at *m/z* 485, (E) Expected fragmentation pattern of the geminal diol of 6'-oxotobramycin.

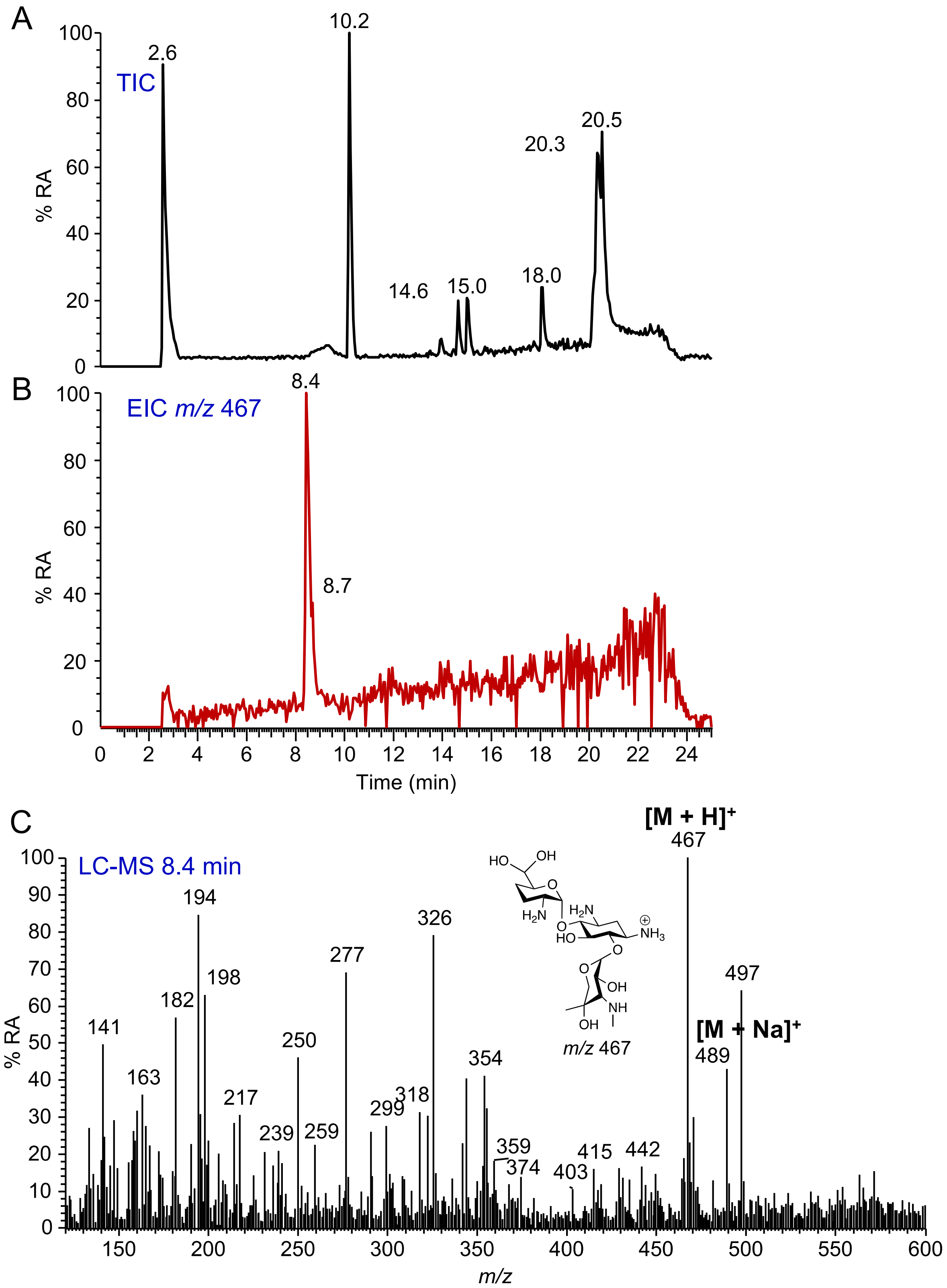
A
TIC
2.6
10.2
14.6
15.0
18.0
20.3
20.5
% RA
B
EIC m/z 467
8.4
8.7
Time (min)
C
LC-MS 8.4 min
[M + H]+
467
[M + Na]+
489
497
141
163
182
194
198
217
239
250
259
277
299
318
326
354
359
374
403
415
442
m/z 467
m/z

D

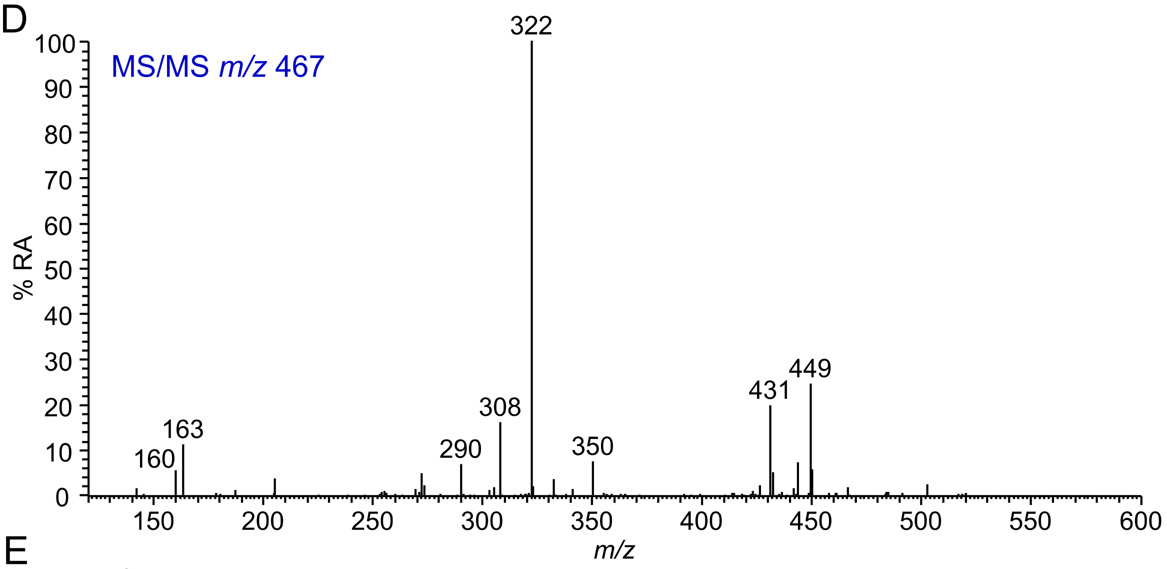

E

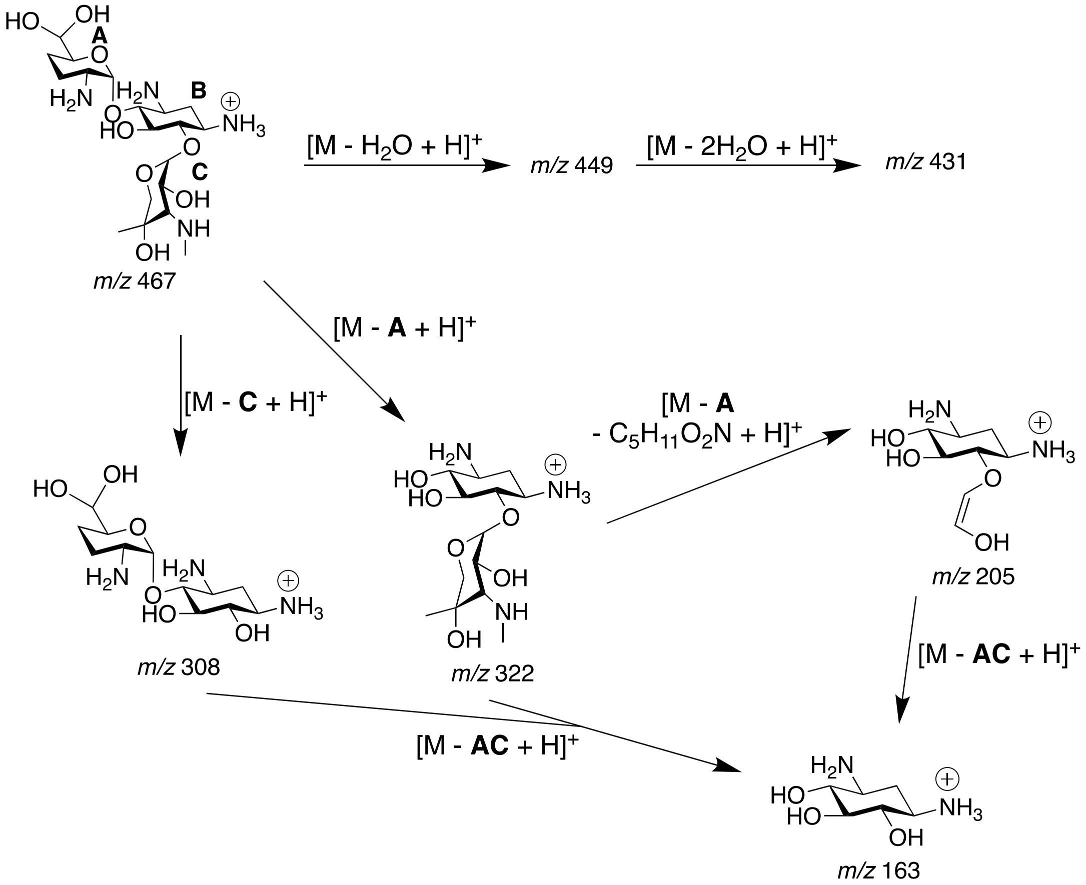

Figure S16: LC-MS and LC-MS/MS analysis of 6'-oxogentamicin C1a obtained from the deamination reaction of gentamicin C1a with GenB2 after 24 hrs. (A) TIC of the reaction, (B) EIC for the mass of the geminal diol of 6'-oxogentamicin C1a (*m/z* 467), (C) LC-MS analysis of the peak at 8.4 min. (D) LC-MS/MS analysis of the mass at *m/z* 467, (E) Expected fragmentation pattern of the geminal diol of 6'-oxogentamicin C1a.

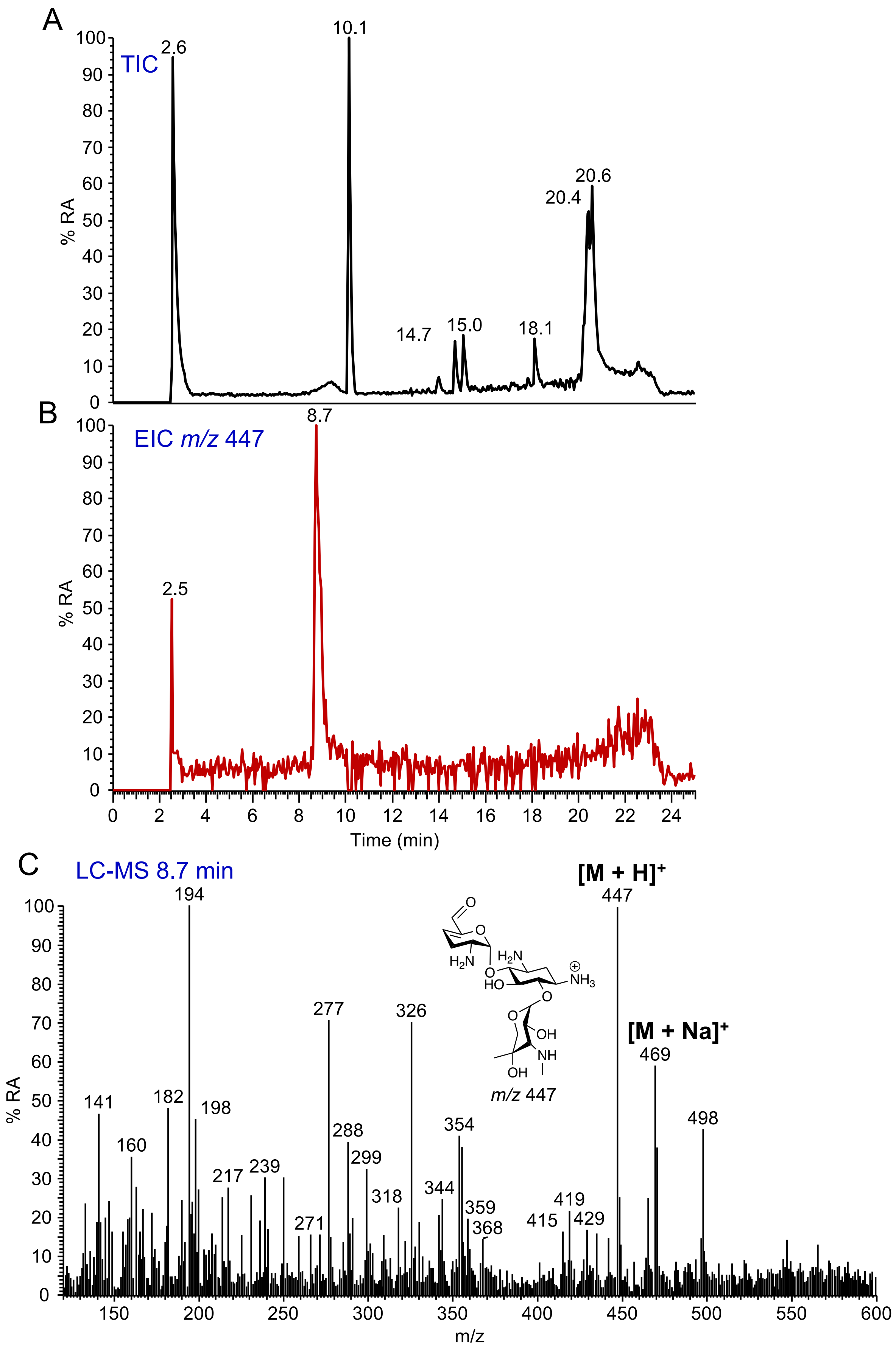
A
TIC
% RA
2.6
10.1
14.7
15.0
18.1
20.4
20.6
B
EIC m/z 447
% RA
2.5
8.7
Time (min)
C
LC-MS 8.7 min
% RA
m/z
[M + H]+
447
[M + Na]+
469
194
141
182
198
160
277
326
288
299
354
217
239
271
318
344
359
368
415
419
429
498
m/z 447

D

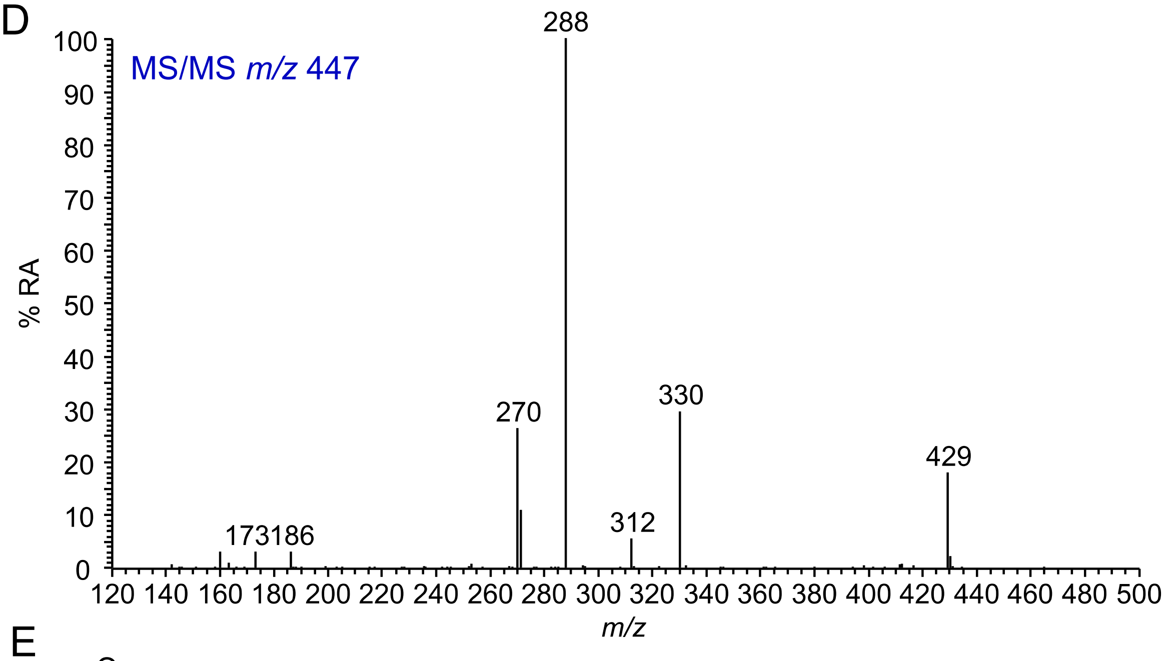

E

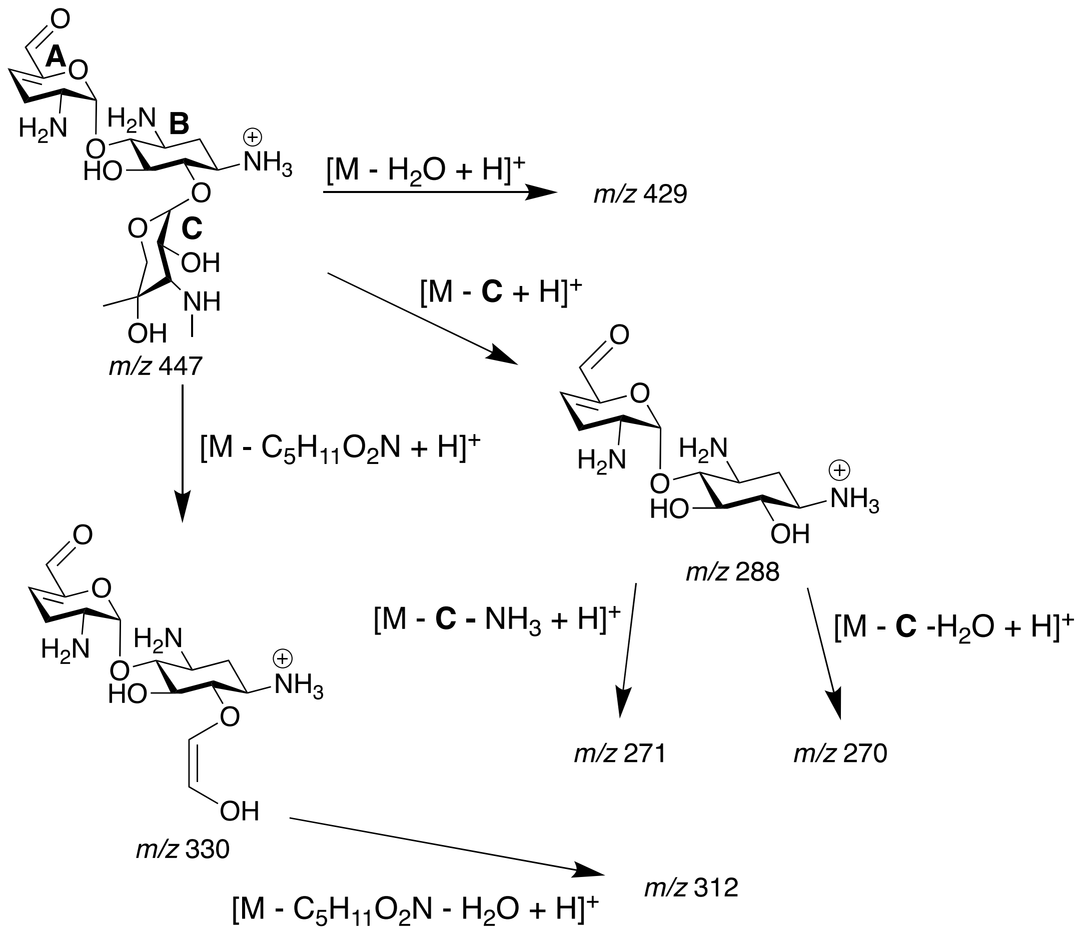

Figure S17: LC-MS and LC-MS/MS analysis of 6'-oxosisomicin obtained from the deamination reaction of sisomicin with NeoB after 24 hrs. (A) TIC of the reaction, (B) EIC for the mass of the 6'-oxosisomicin (*m/z* 447), (C) LC-MS analysis of the peak at 8.7 min. (D) LC-MS/MS analysis of the mass at *m/z* 447, (E) Expected fragmentation pattern of the 6'-oxosisomicin.

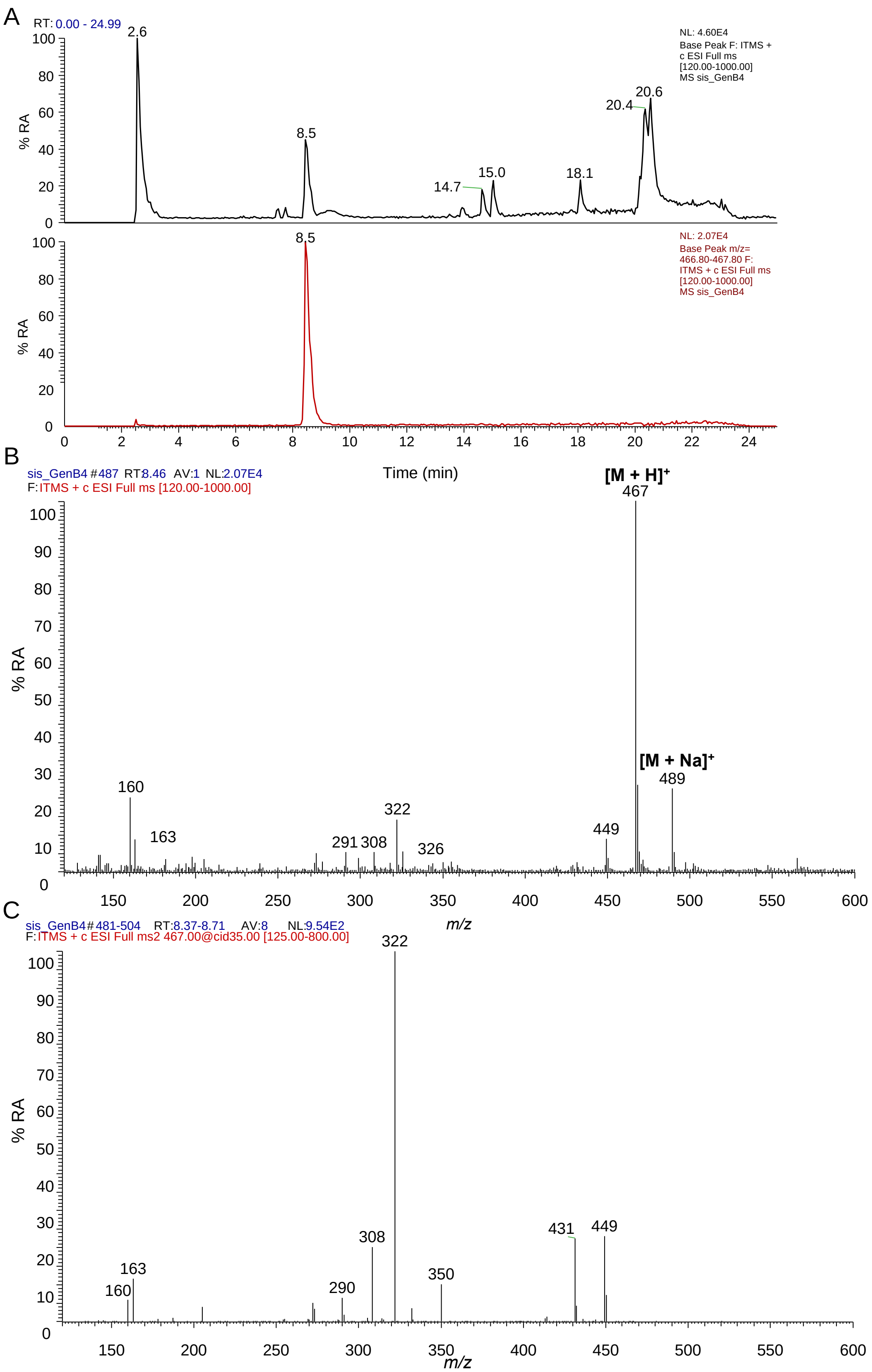

Figure S18: LC-MS analysis of 6'-oxogentamicin C1a obtained from the deamination reaction of sisomicin with GenB4 after 24 hrs. (A) TIC and EIC for the mass of the 6'-oxogentamicin C1a (*m/z* 467), (B) LC-MS analysis of the peak at 8.5 min, (C) LC-MS/MS analysis of the mass at *m/z* 467.

6. NMR and HRMS Spectral Data

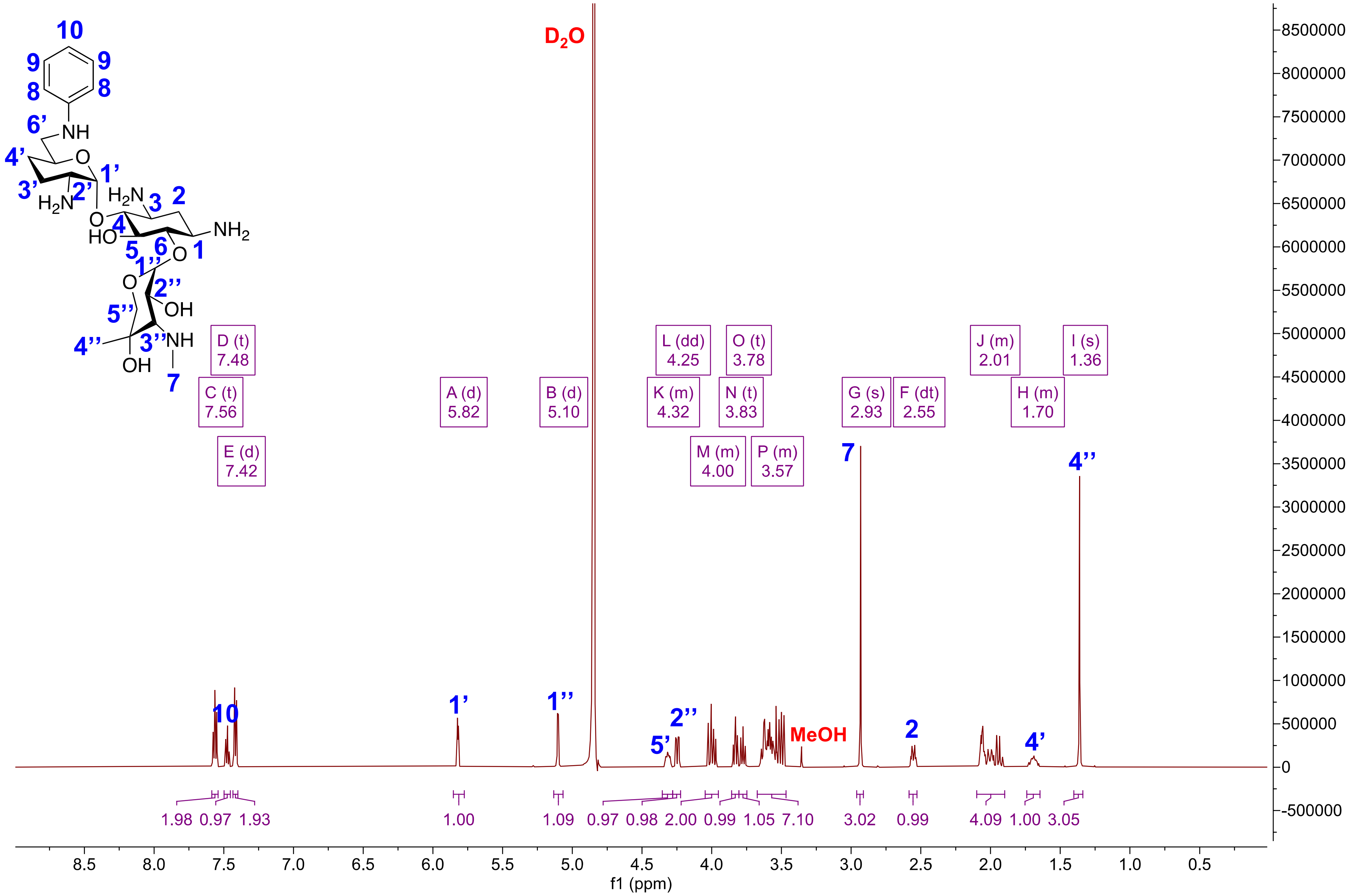

Figure S19: ^{1}H NMR spectroscopic analysis of 6'-*N*-(phenyl)-gentamicin C1a (**4a**).

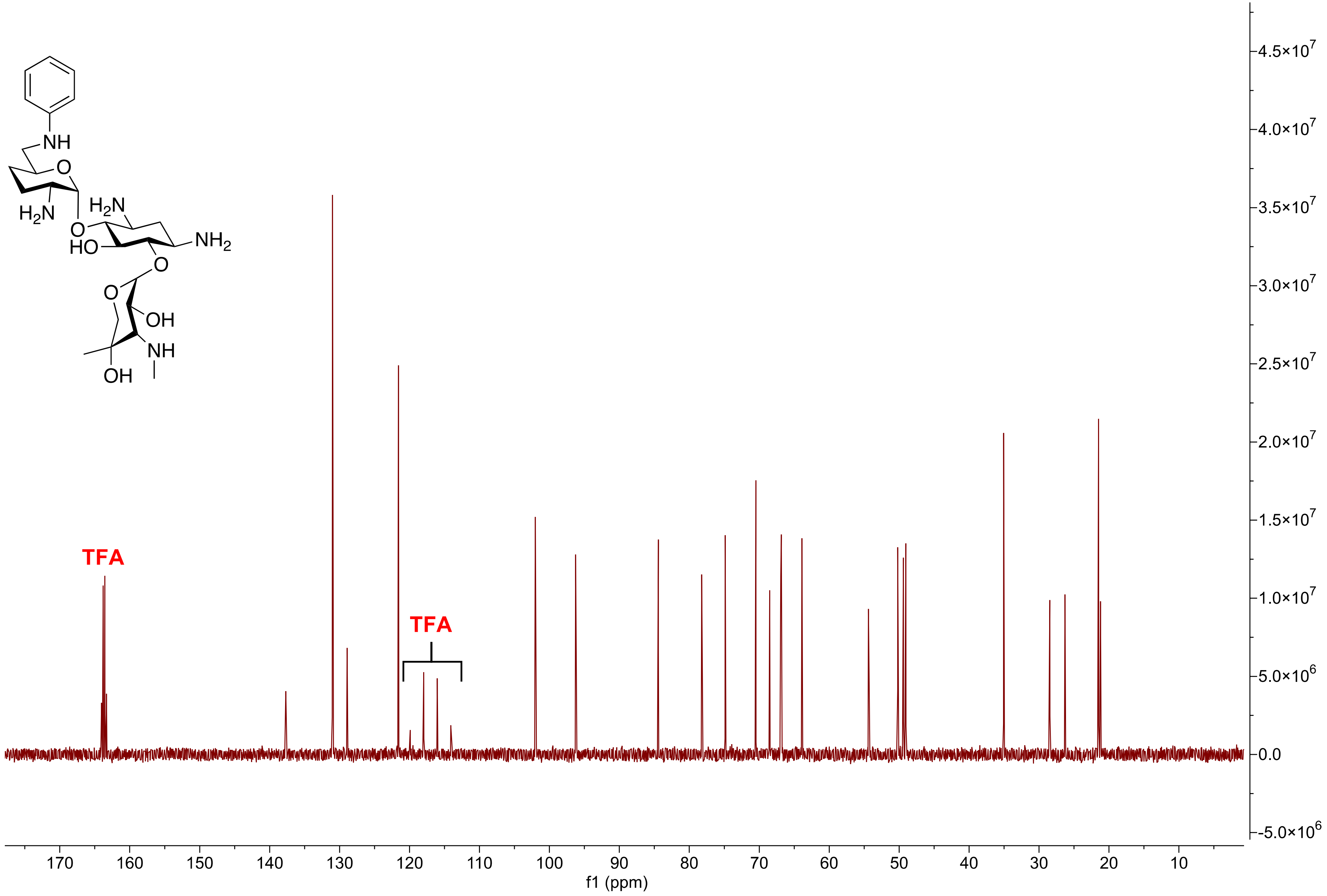

Figure S20: ^{13}C NMR spectroscopic analysis of 6’-*N*-(phenyl)-gentamicin C1a (**4a**).

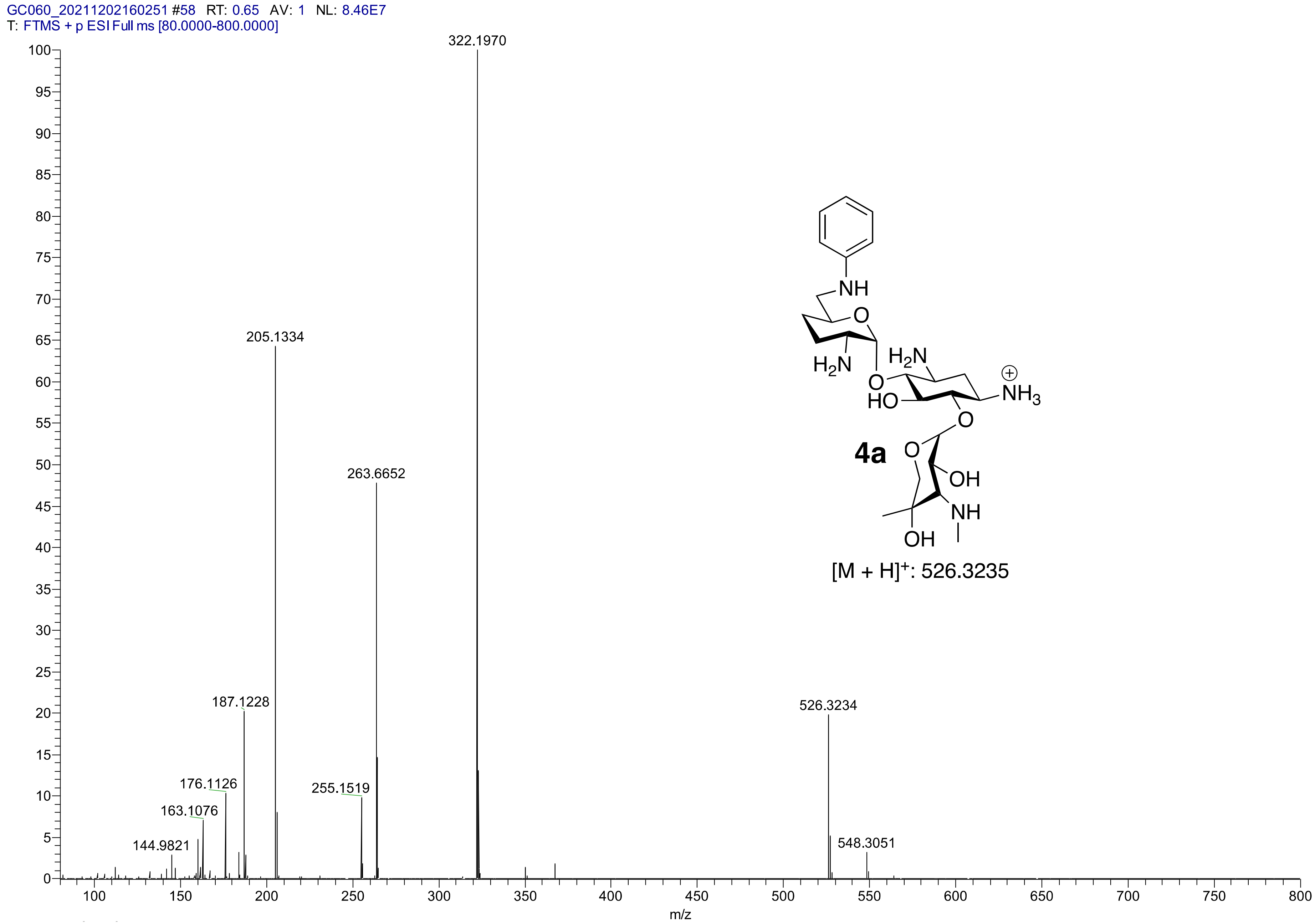

Figure S21: HRMS analysis of 6'-*N*-(phenyl)-gentamicin C1a (**4a**).

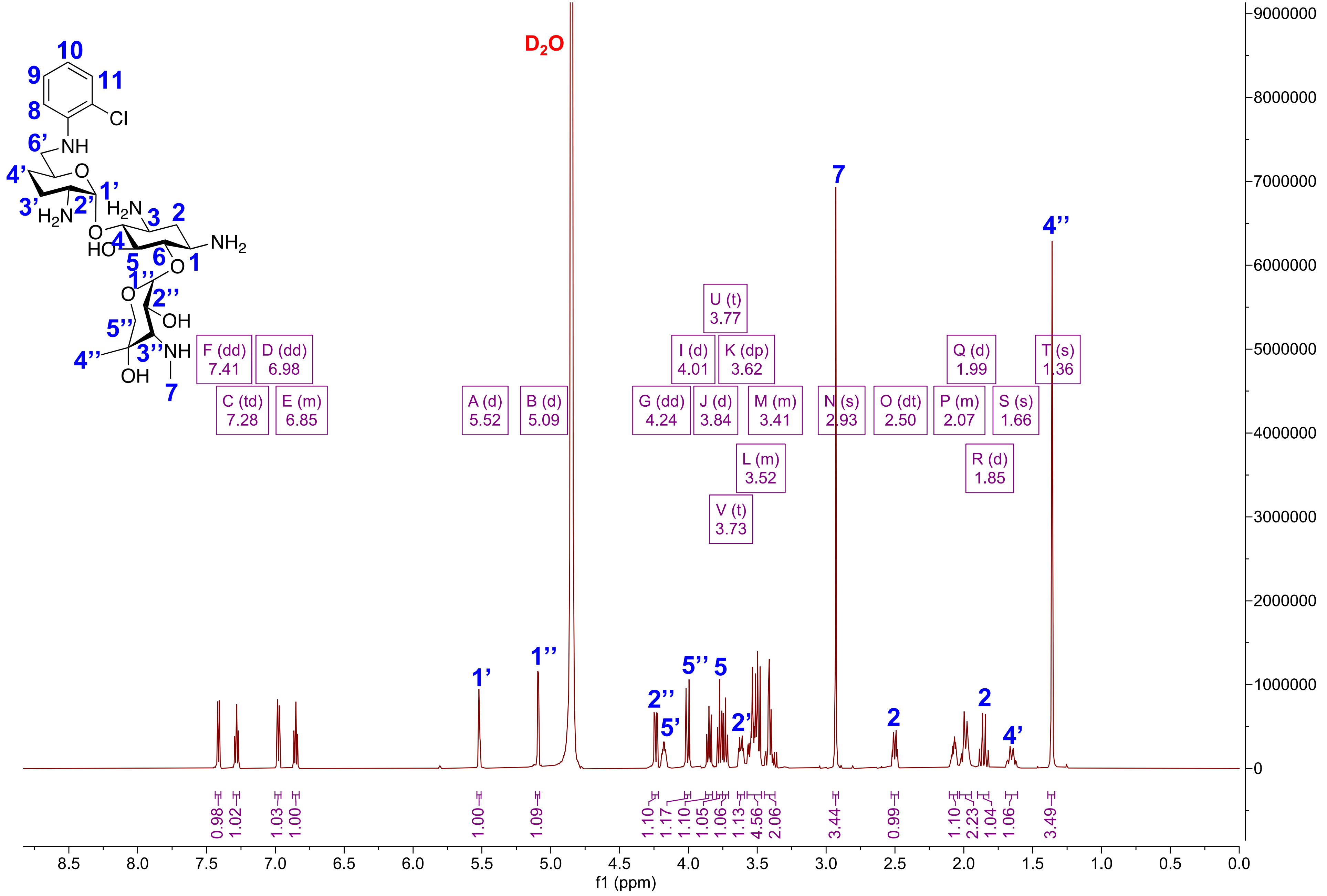

Figure S22: ^{1}H NMR spectroscopic analysis of 6'-*N*-(2-chlorophenyl)-gentamicin C1a (**4b**).

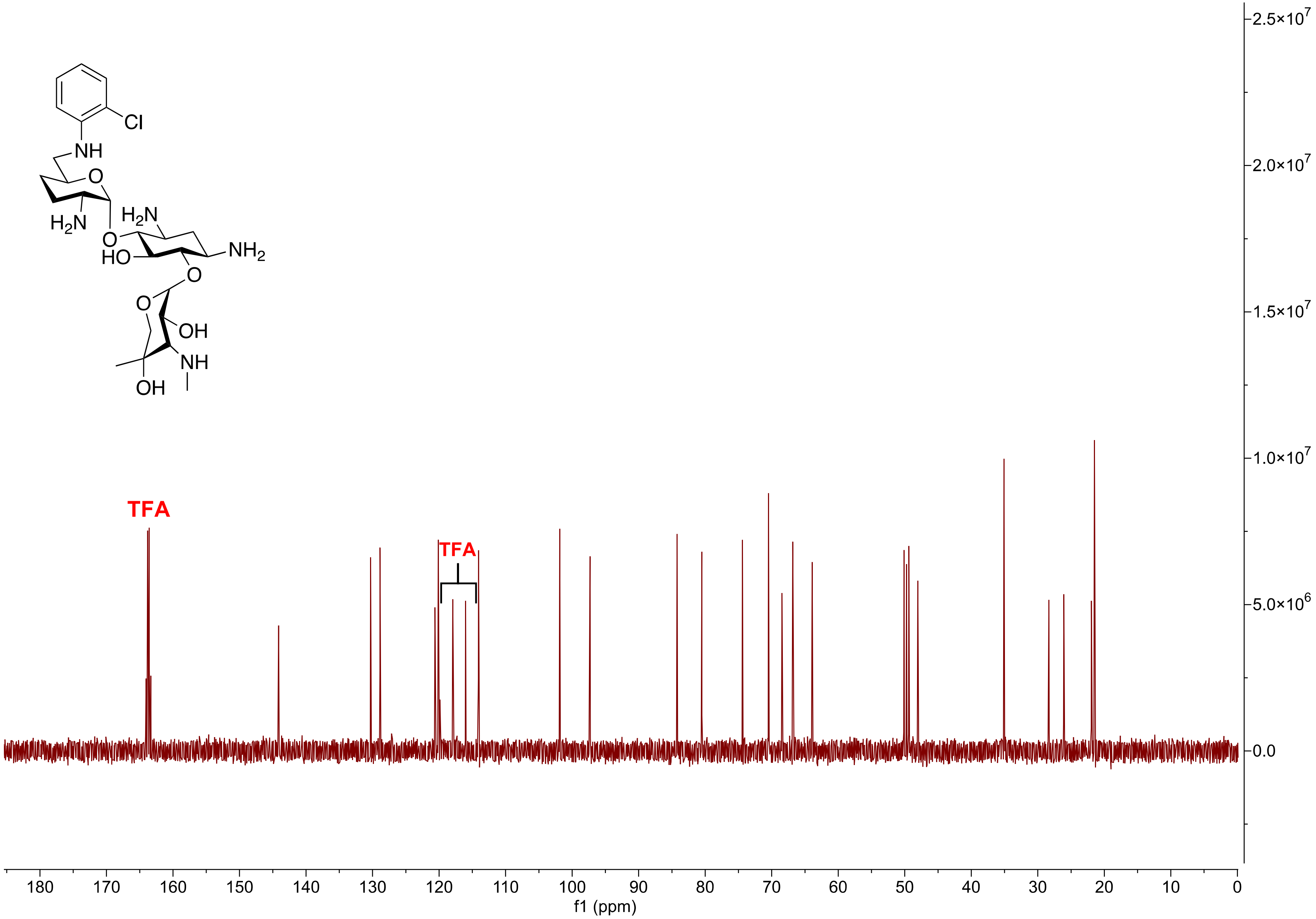

Figure S23: ^{13}C NMR spectroscopic analysis of 6'-*N*-(2-chlorophenyl)-gentamicin C1a (**4b**).

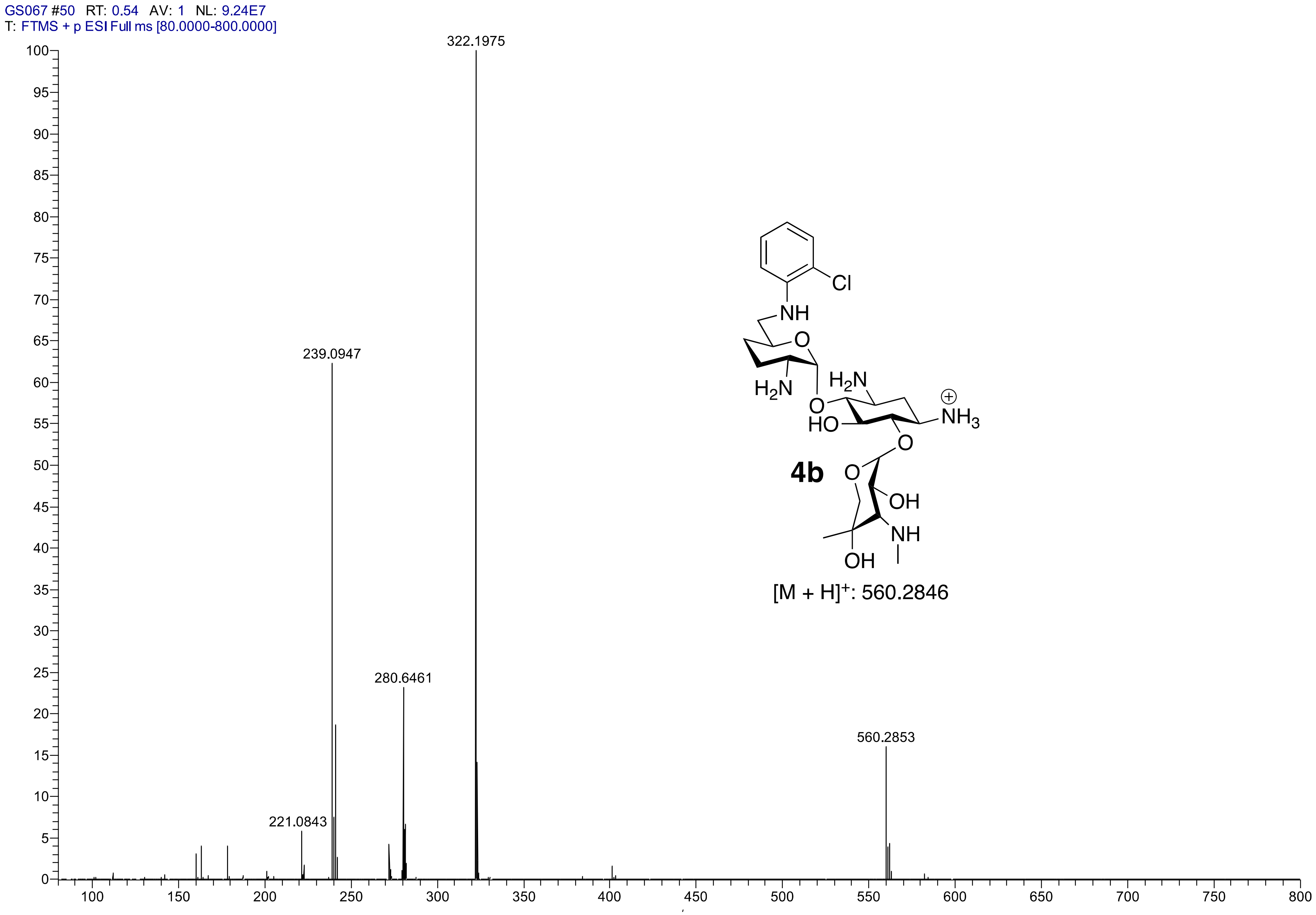

Figure S24: HRMS analysis of 6'-*N*-(2-chlorophenyl)-gentamicin C1a (**4b**).

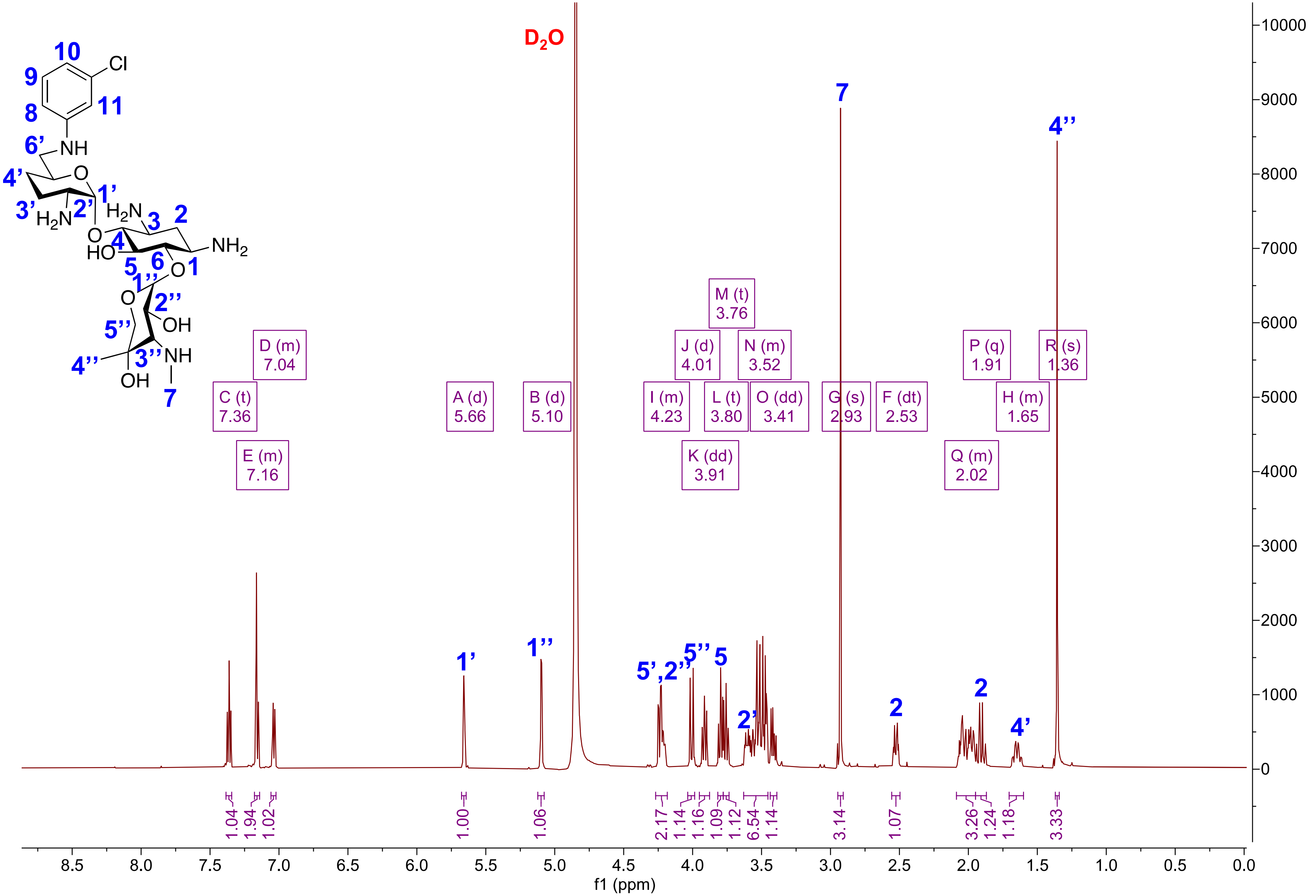

Figure S25: ^{1}H NMR spectroscopic analysis of 6'-*N*-(3-chlorophenyl)-gentamicin C1a (**4c**).

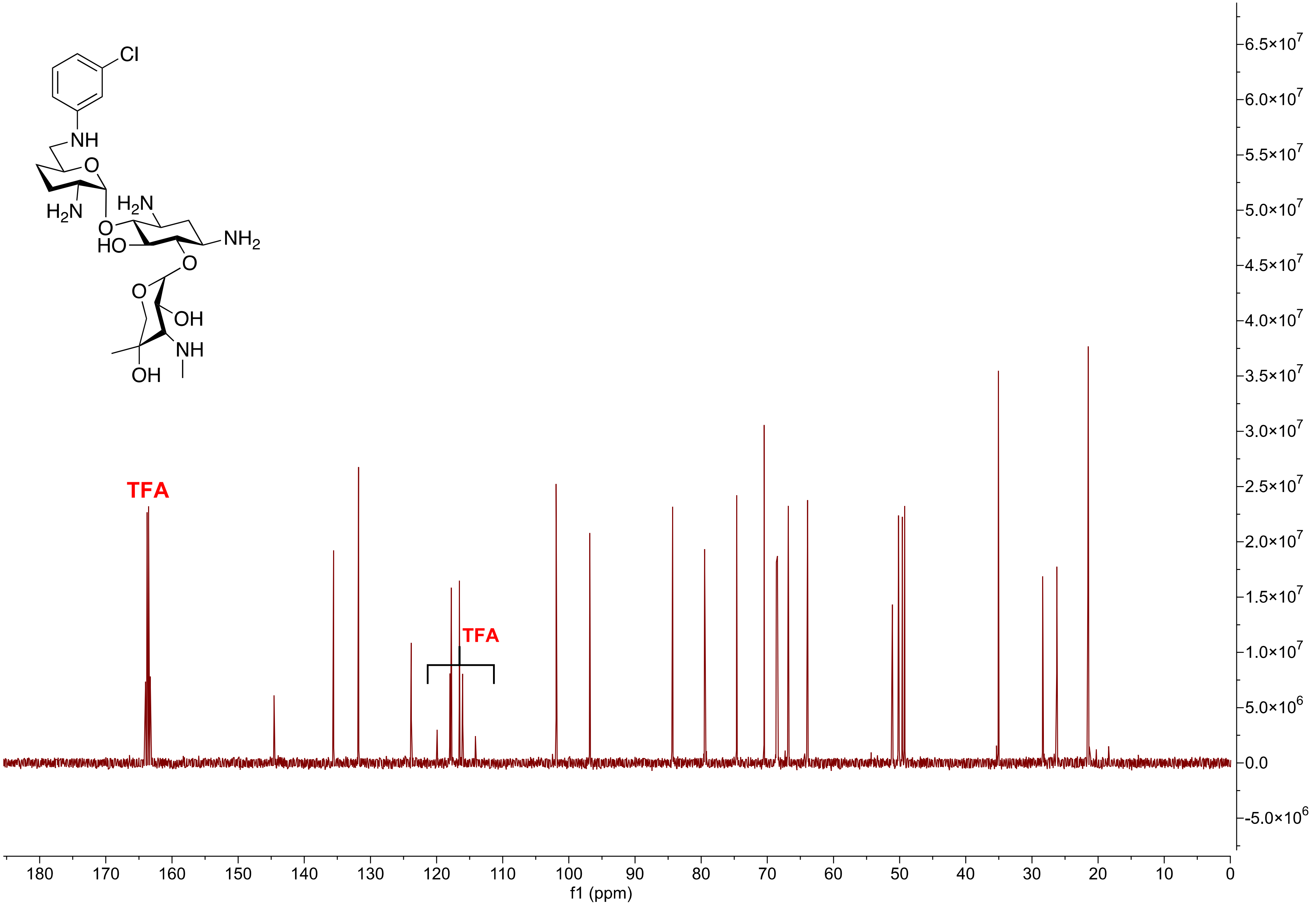

Figure S26: ^{13}C NMR spectroscopic analysis of 6'-*N*-(3-chlorophenyl)-gentamicin C1a (**4c**).

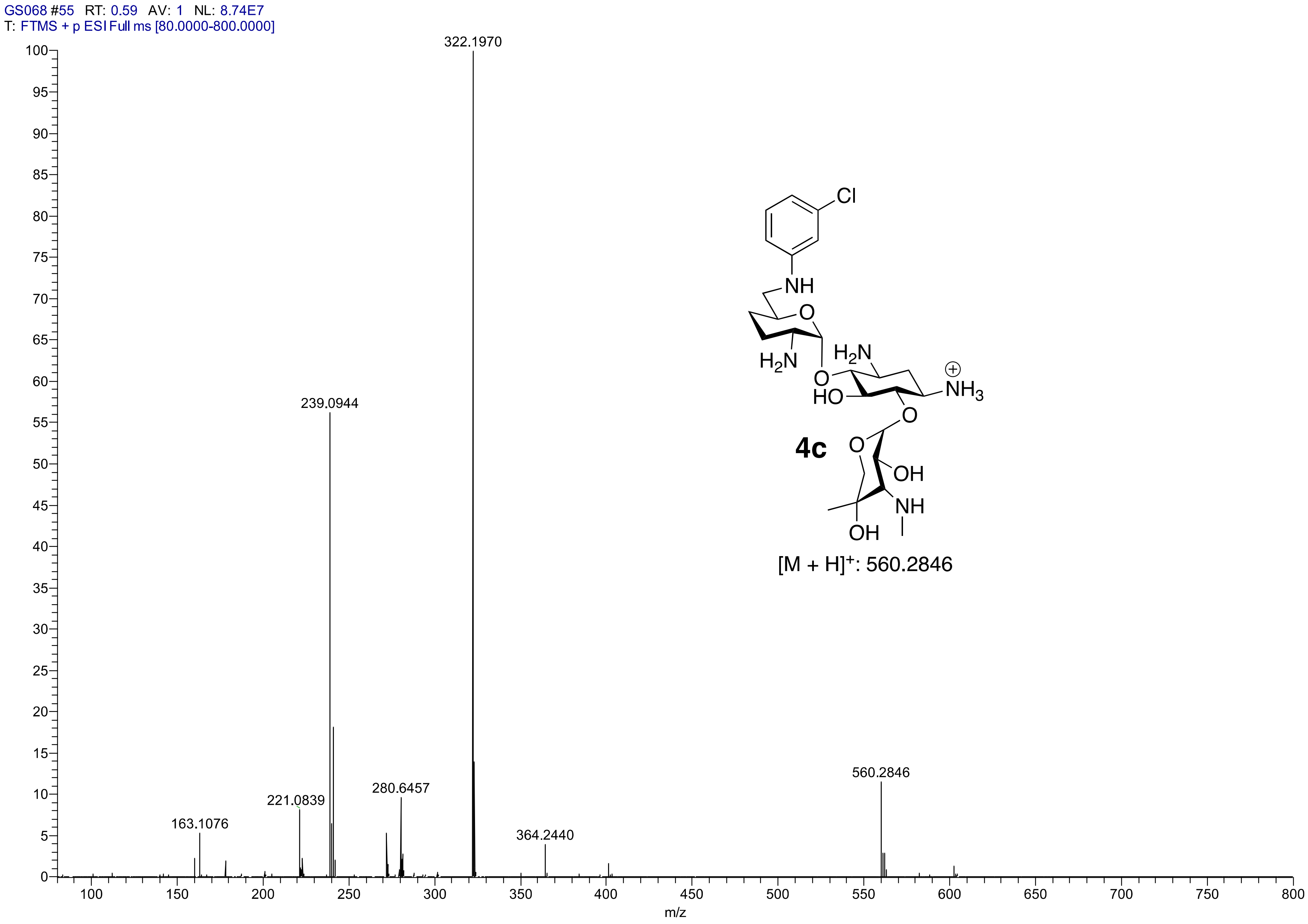

Figure S27: HRMS analysis of 6'-*N*-(3-chlorophenyl)-gentamicin C1a (**4c**).

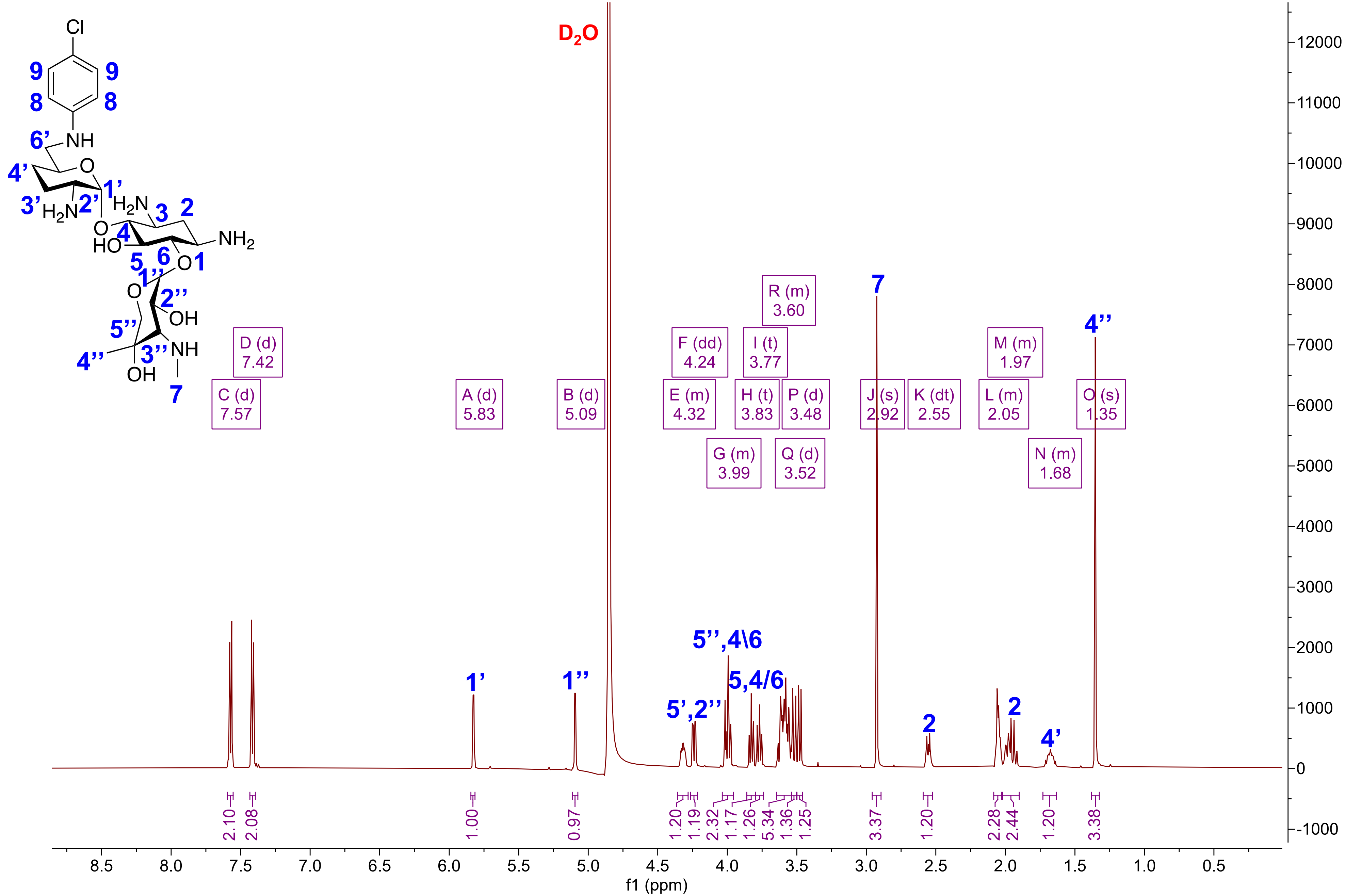

Figure S28: ^{1}H NMR spectroscopic analysis of 6’-*N*-(4-chlorophenyl)-gentamicin C1a (**4d**).

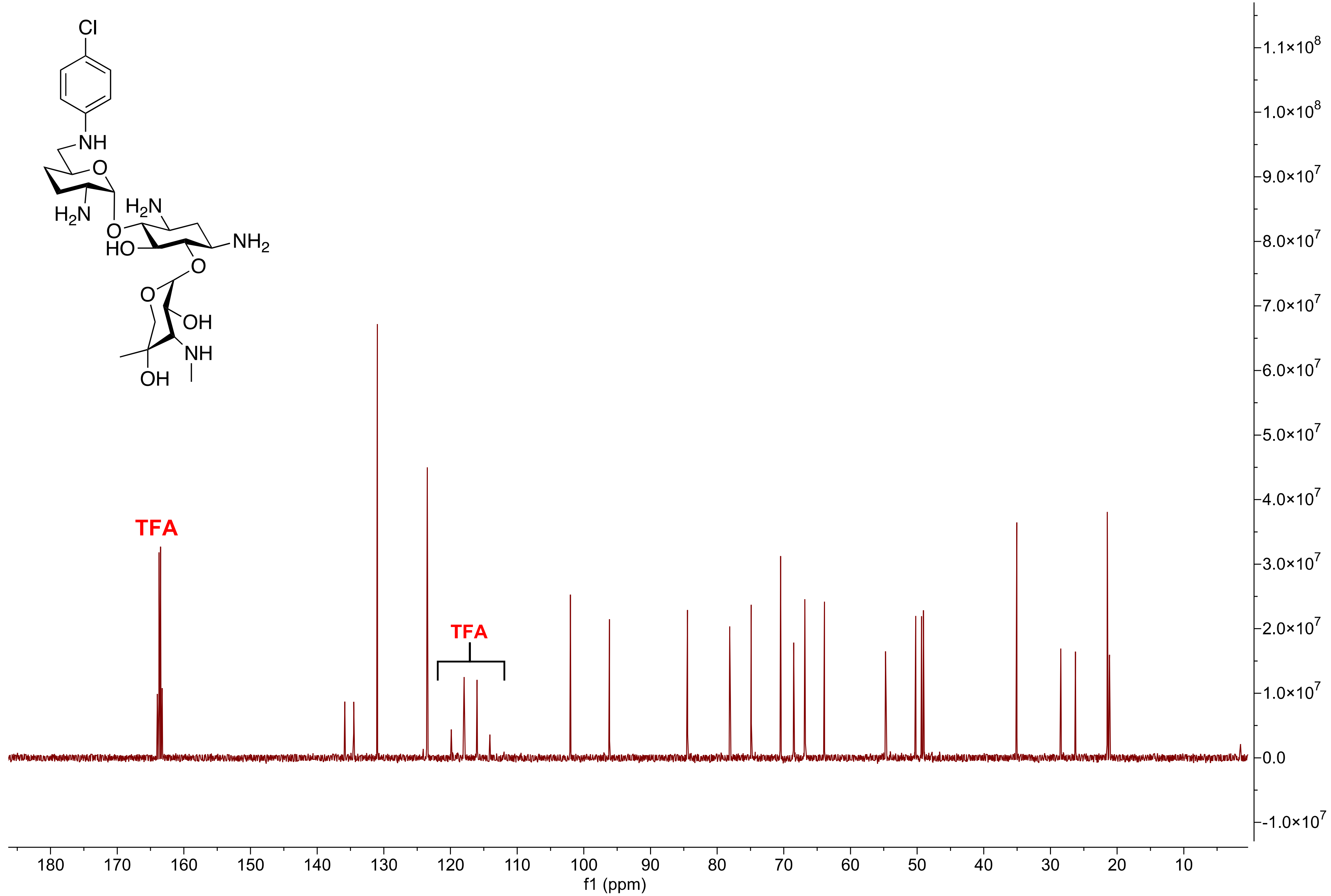

Figure S29: ^{13}C NMR spectroscopic analysis of 6'-*N*-(4-chlorophenyl)-gentamicin C1a (**4d**).

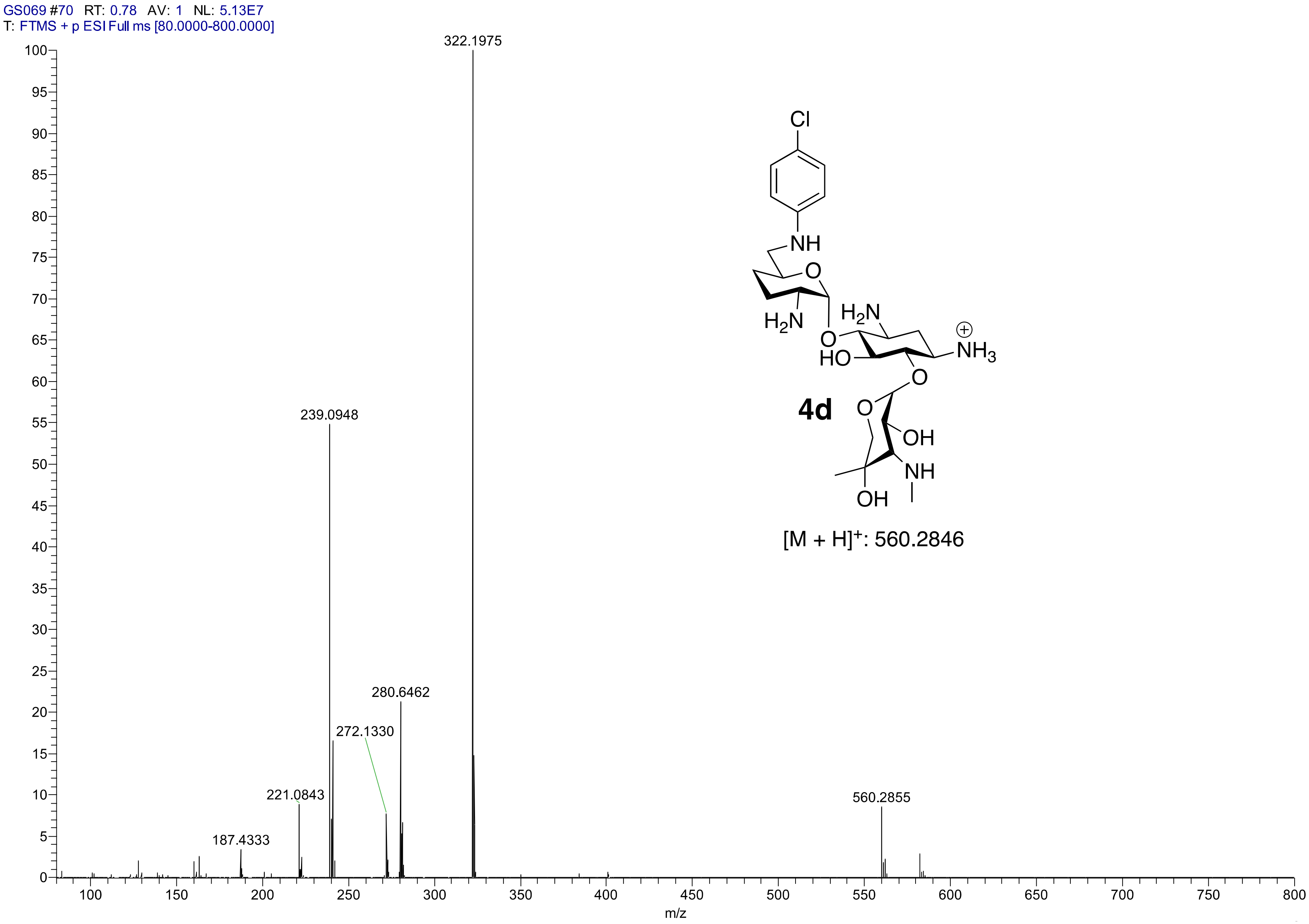

Figure S30: HRMS analysis of 6'-*N*-(4-chlorophenyl)-gentamicin C1a (**4d**).

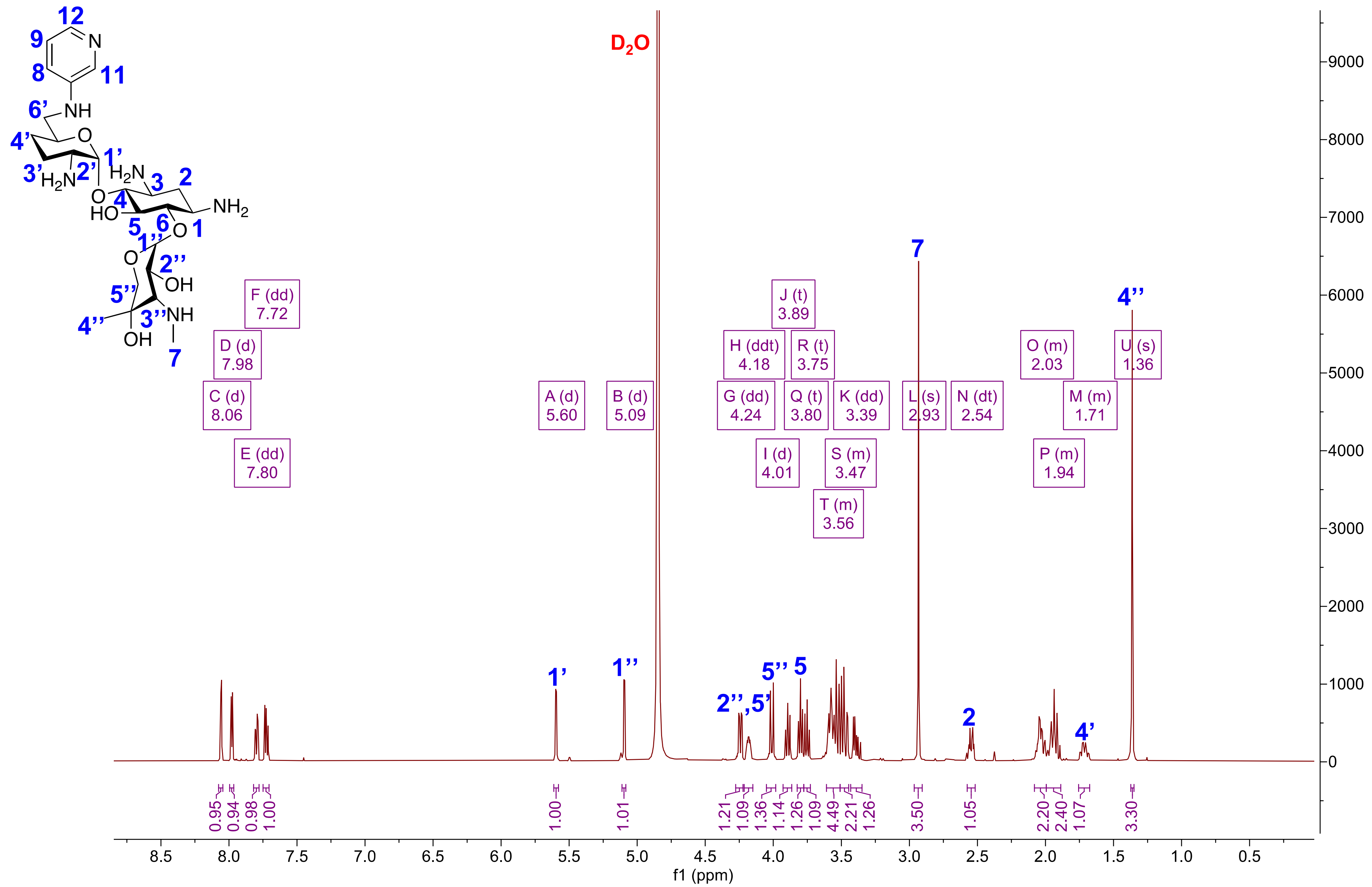

Figure S31: ^{1}H NMR spectroscopic analysis of 6'-*N*-(Pyridin-3-ylamino)-gentamicin C1a (**4e**).

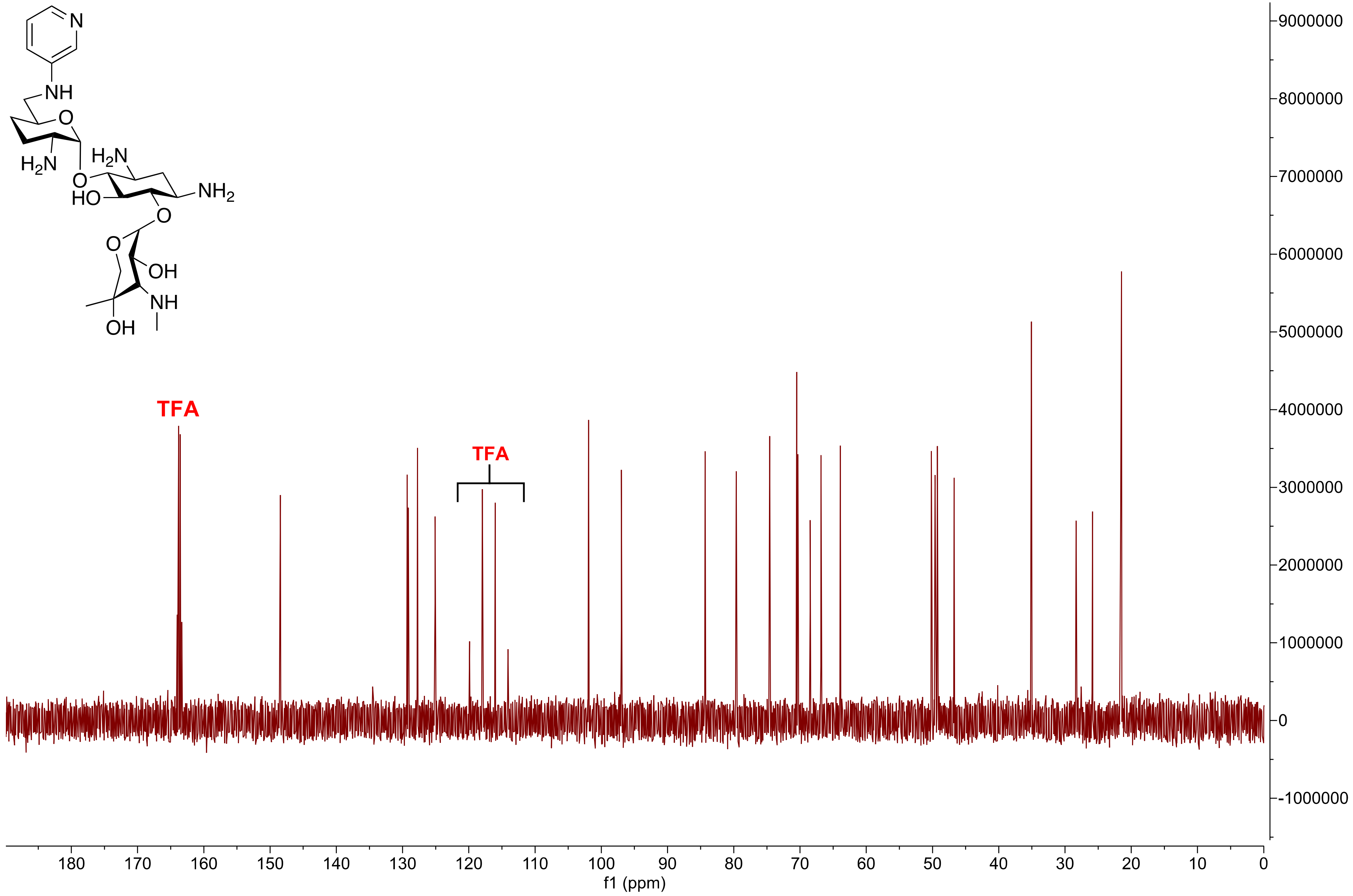

Figure S32: ^{13}C NMR spectroscopic analysis of 6’-*N*-(Pyridin-3-ylamino)-gentamicin C1a (**4e**).

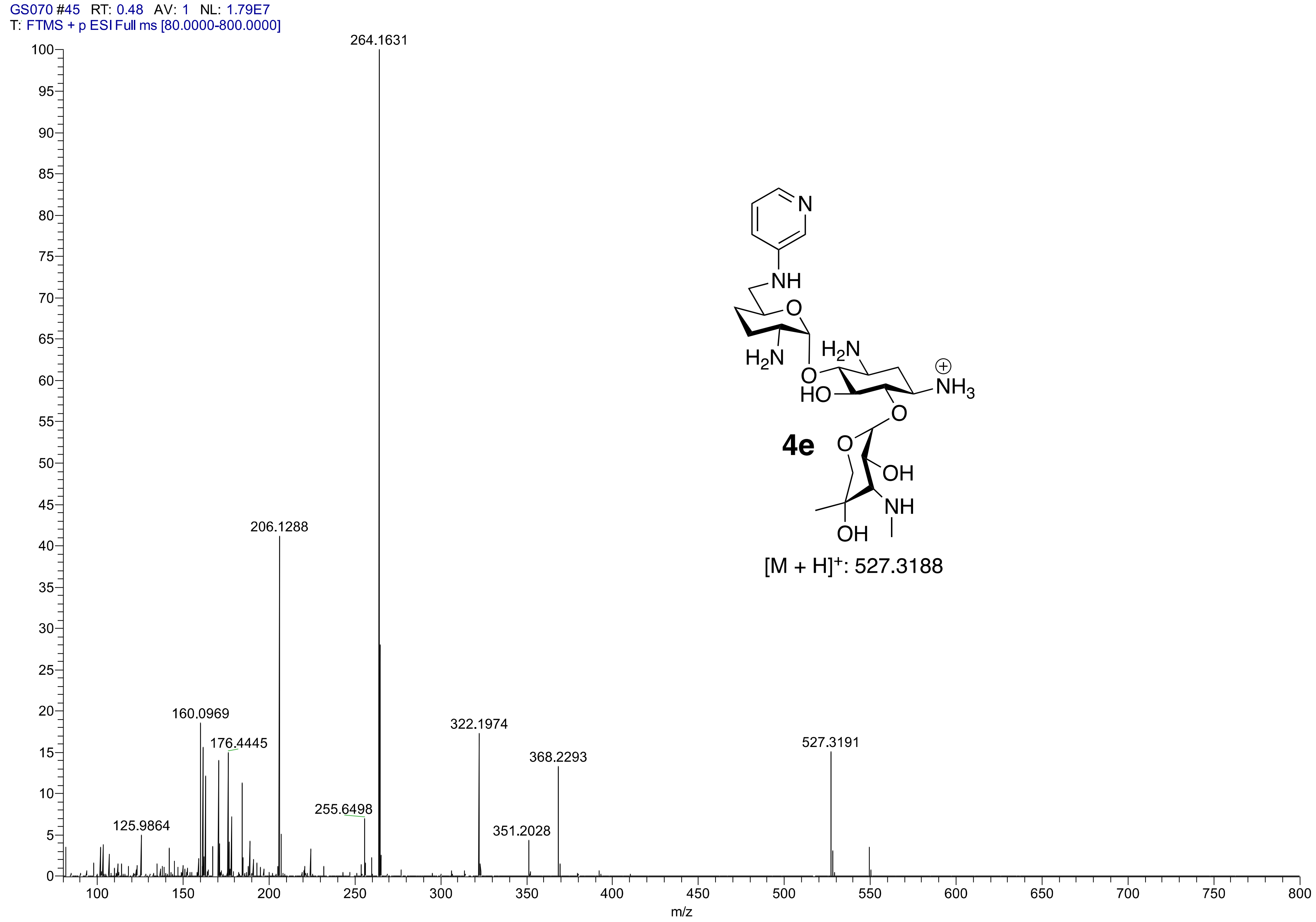

Figure S33: HRMS analysis of 6'-*N*-(Pyridin-3-ylamino)-gentamicin C1a (**4e**).

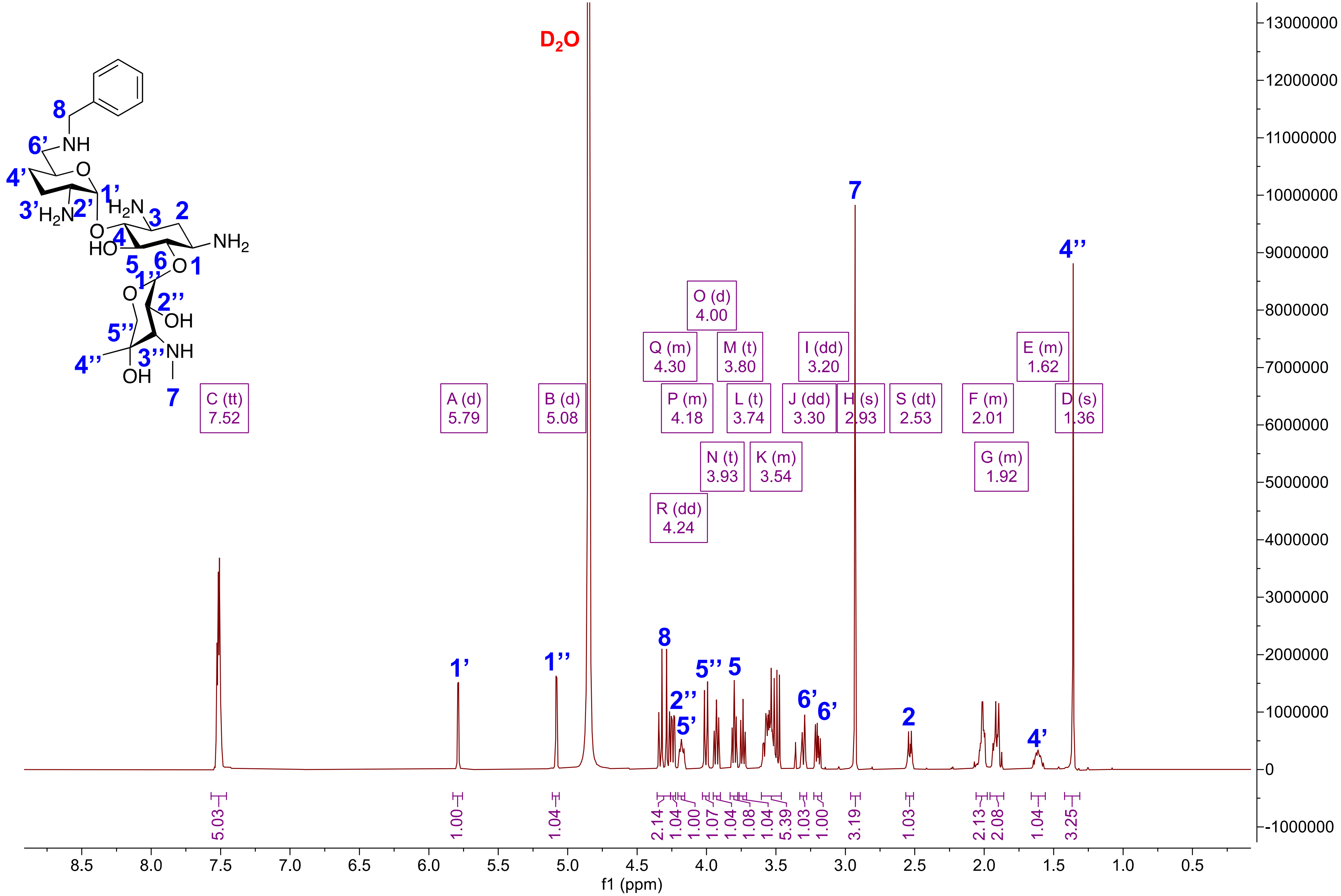

Figure S34: ^{1}H NMR spectroscopic analysis of 6'-*N*-(Benzyl)-gentamicin C1a (**4f**).

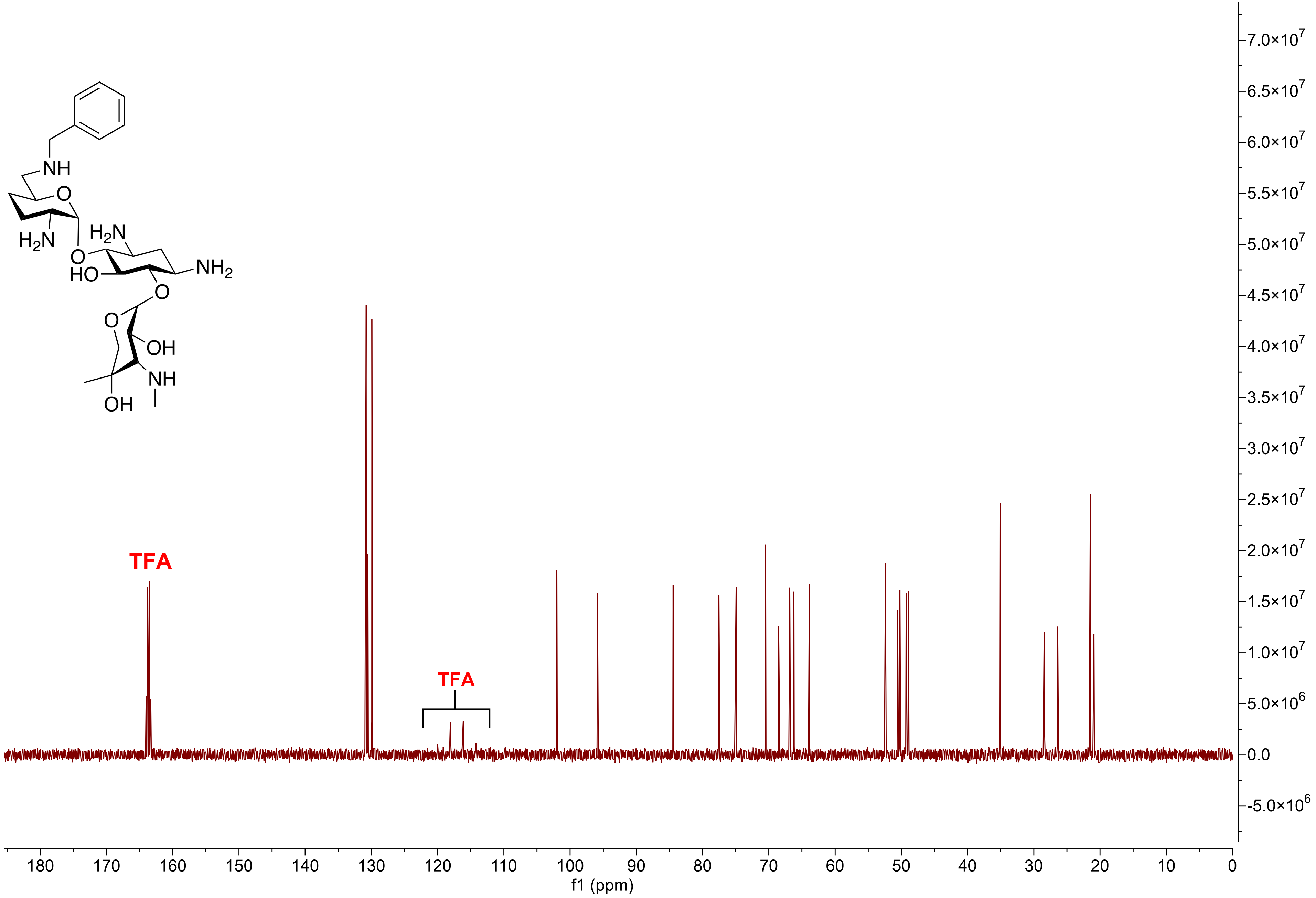

Figure S35: ^{13}C NMR spectroscopic analysis of 6’-*N*-(Benzyl)-gentamicin C1a (**4f**).

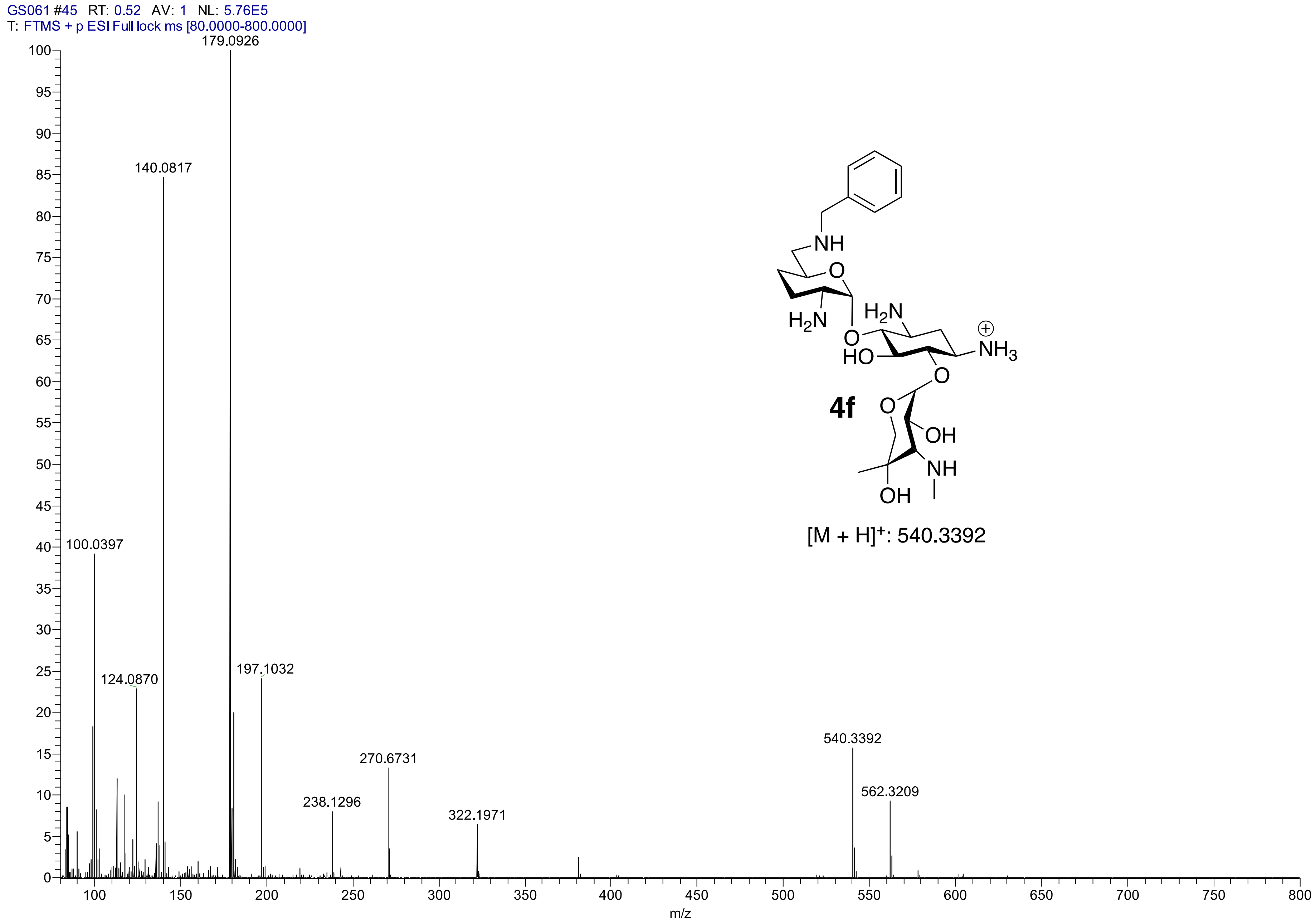

Figure S36: HRMS analysis of 6'-*N*-(Benzyl)-gentamicin C1a (**4f**).

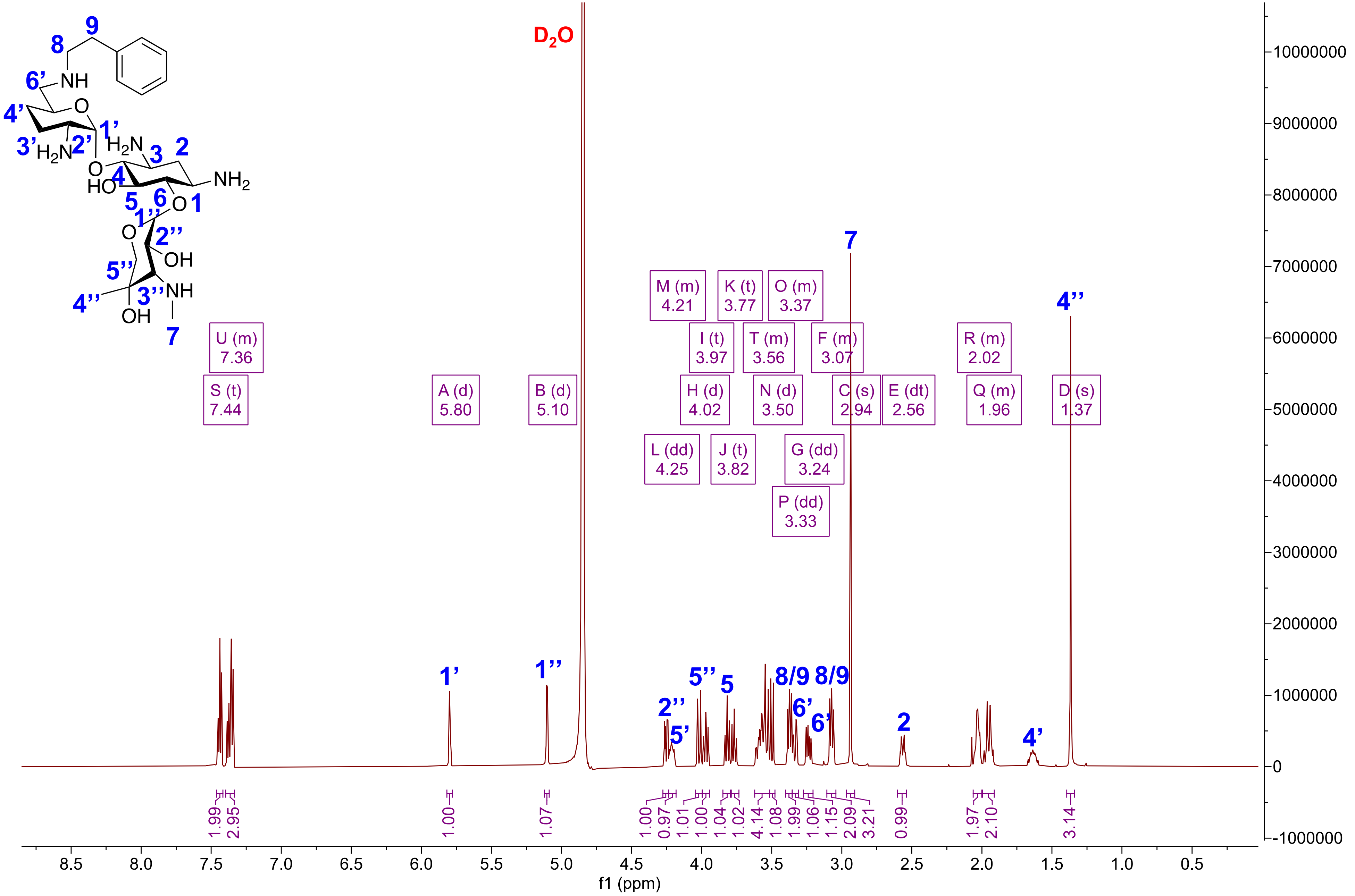

Figure S37: ^{1}H NMR spectroscopic analysis of 6'-*N*-(Phenethyl)-gentamicin C1a (**4g**).

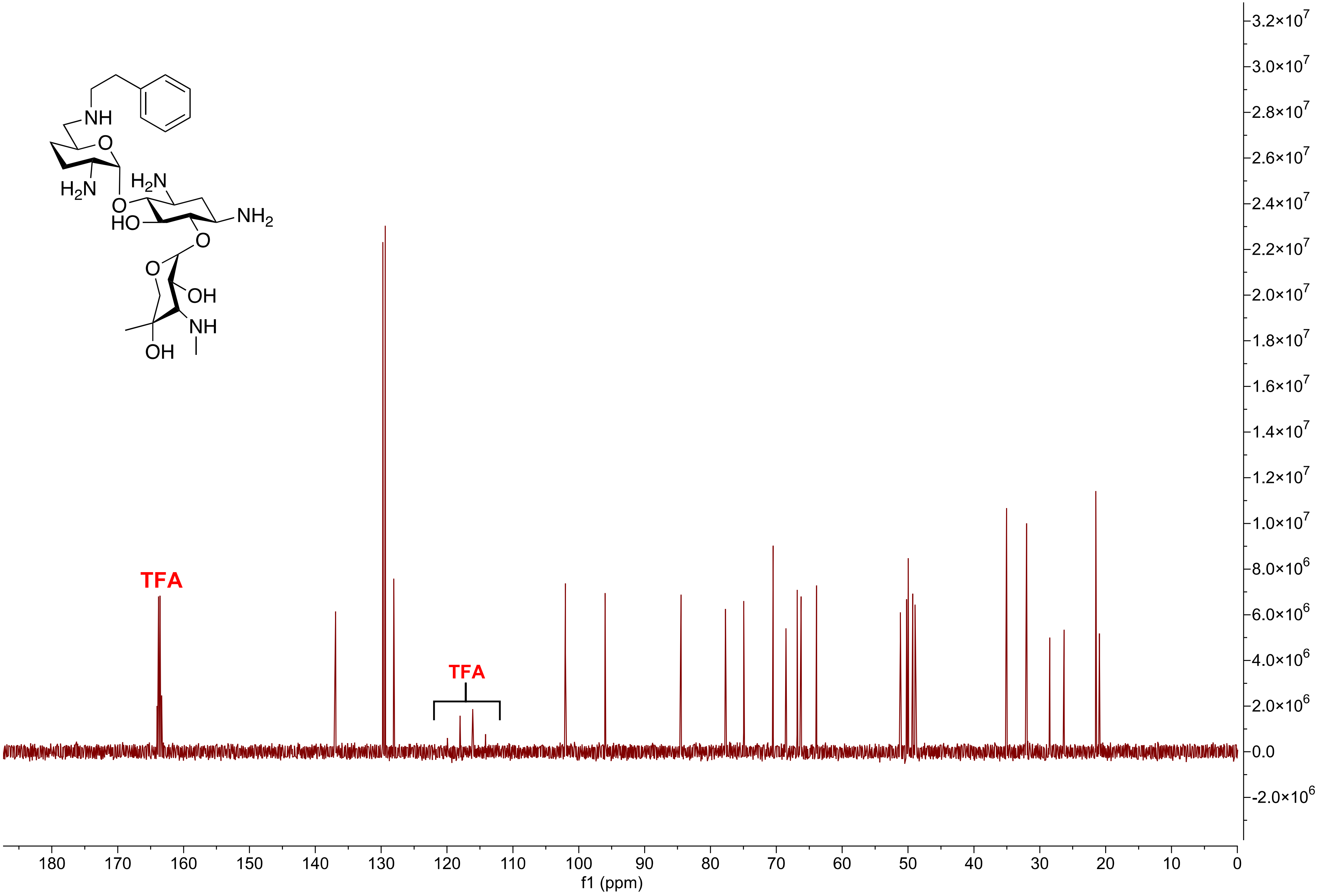

Figure S38: ^{13}C NMR spectroscopic analysis of 6'-*N*-(Phenethyl)-gentamicin C1a (**4g**).

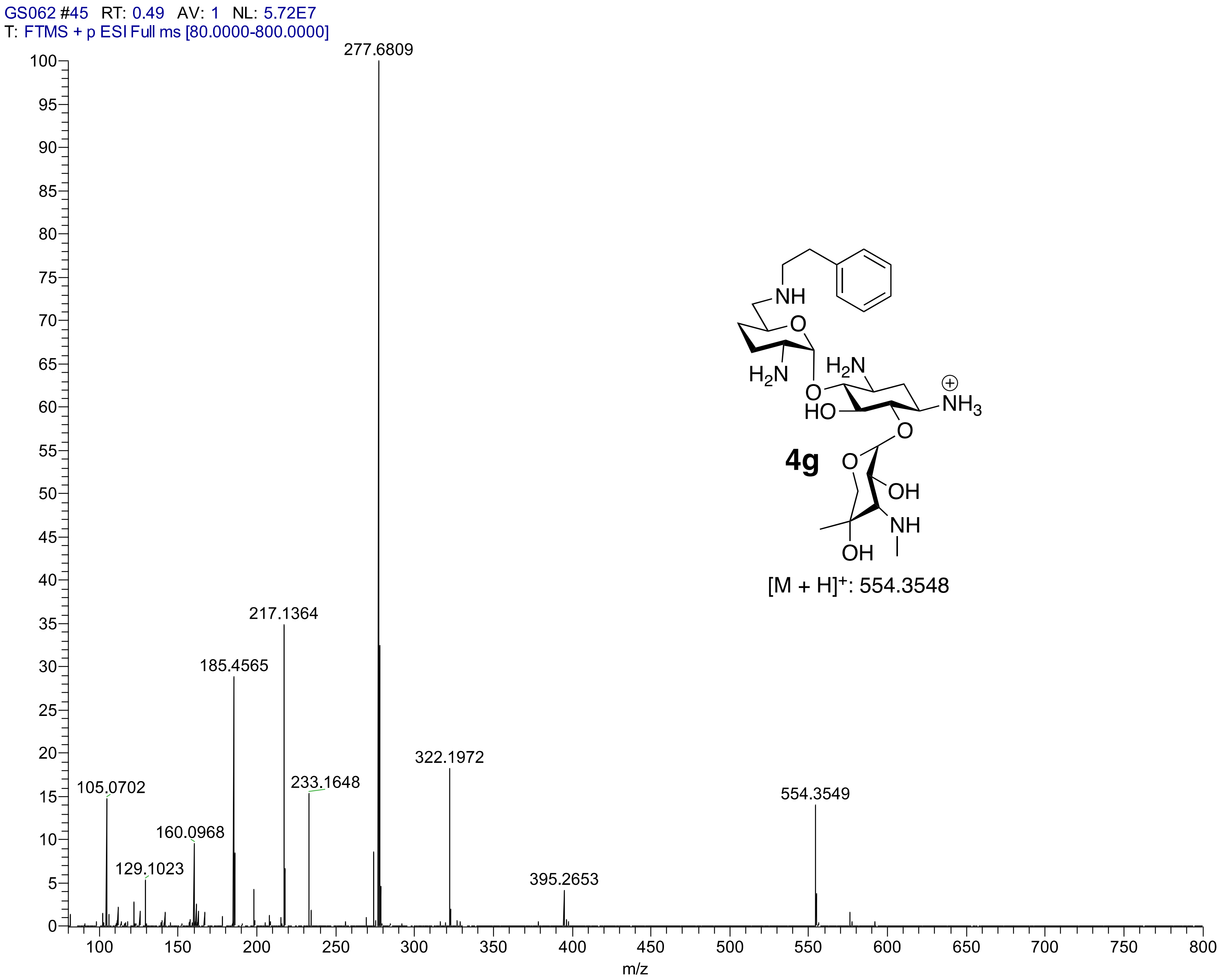

Figure S39: ^{13}C NMR spectroscopic analysis of 6'-*N*-(Phenethyl)-gentamicin C1a (**4g**).

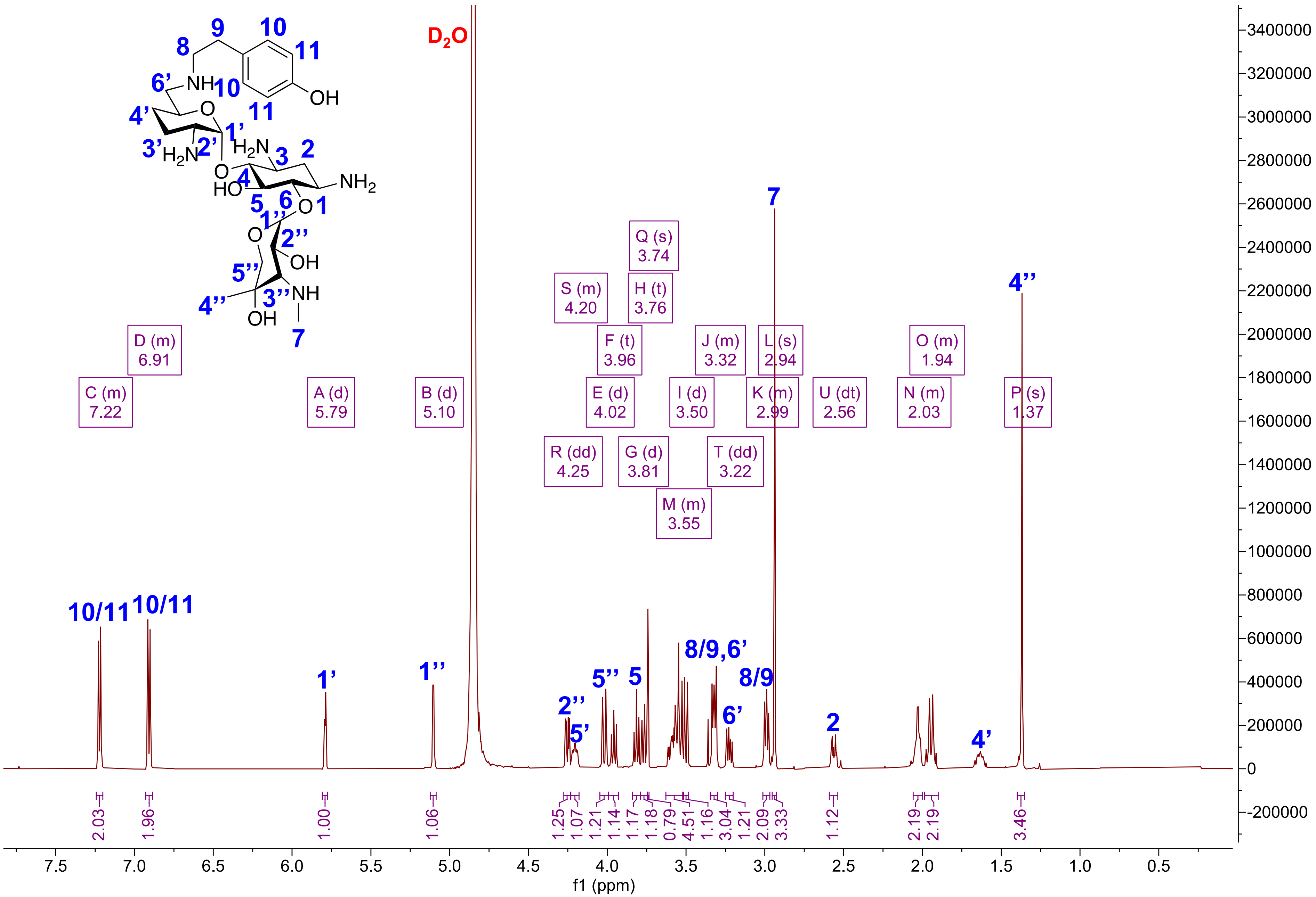

Figure S40: ^{1}H NMR spectroscopic analysis of 6'-*N*-(4-Hydroxyphenethyl)-gentamicin C1a (**4h**).

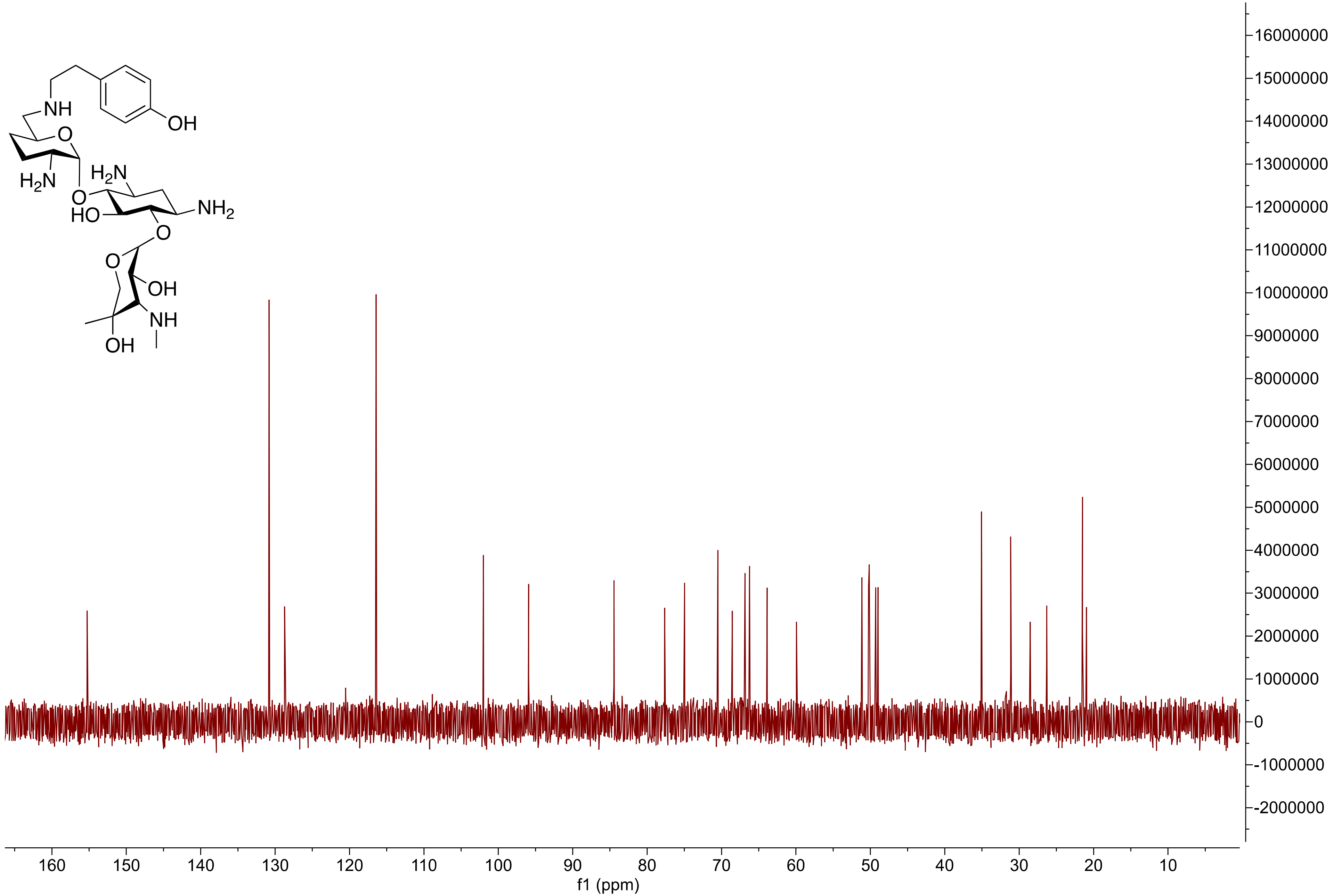

Figure S41: ^{13}C NMR spectroscopic analysis of 6'-*N*-(4-Hydroxyphenethyl)-gentamicin C1a (**4h**).

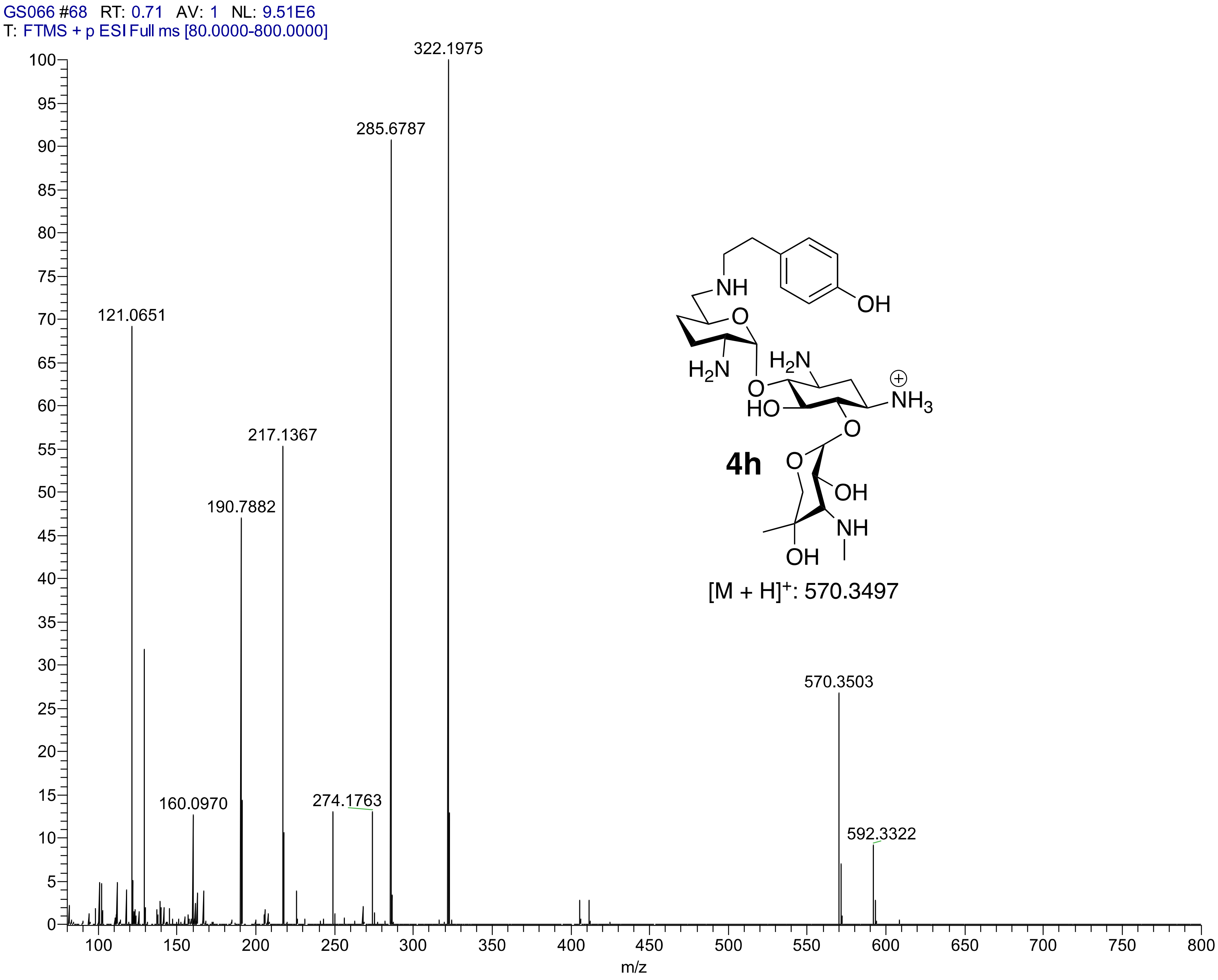

Figure S42: HRMS analysis of 6’-*N*-(4-Hydroxyphenethyl)-gentamicin C1a (**4h**).

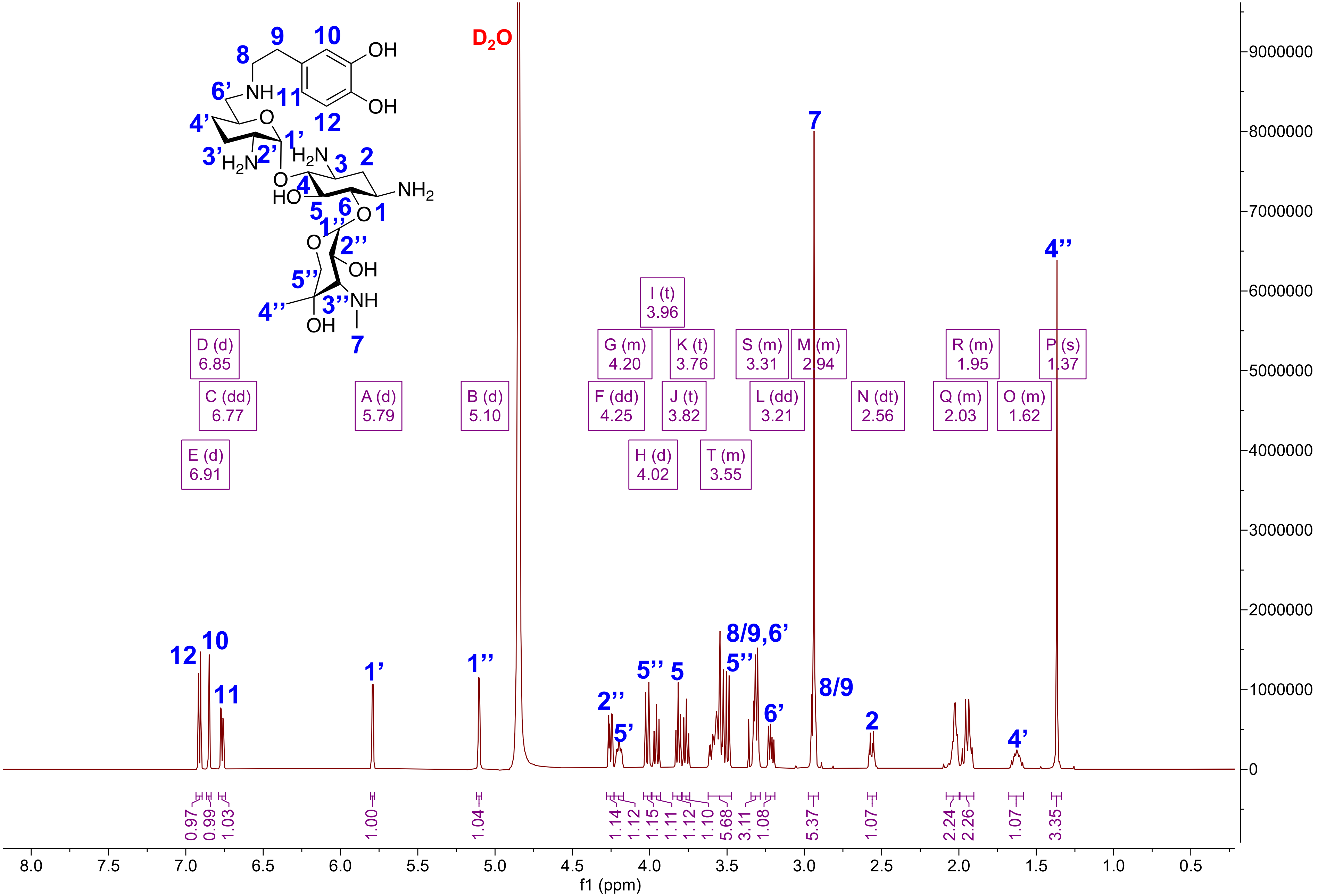

Figure S43: ^{1}H NMR spectroscopic analysis of 6'-*N*-(3,4-Dihydroxyphenethyl)-gentamicin C1a (**4i**).

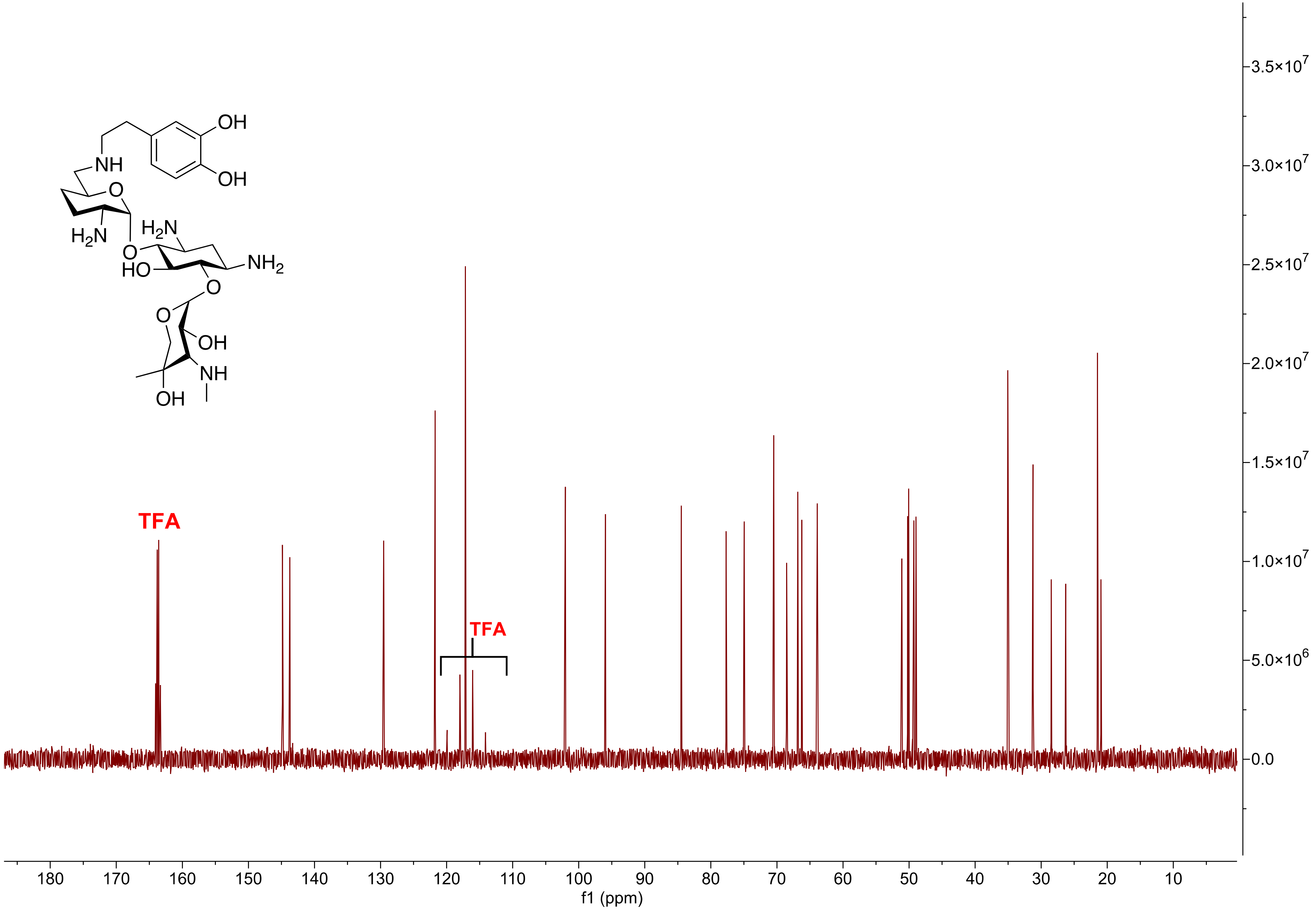

Figure S44: ^{13}C NMR spectroscopic analysis of 6’-*N*-(3,4-Dihydroxyphenethyl)-gentamicin C1a (**4i**).

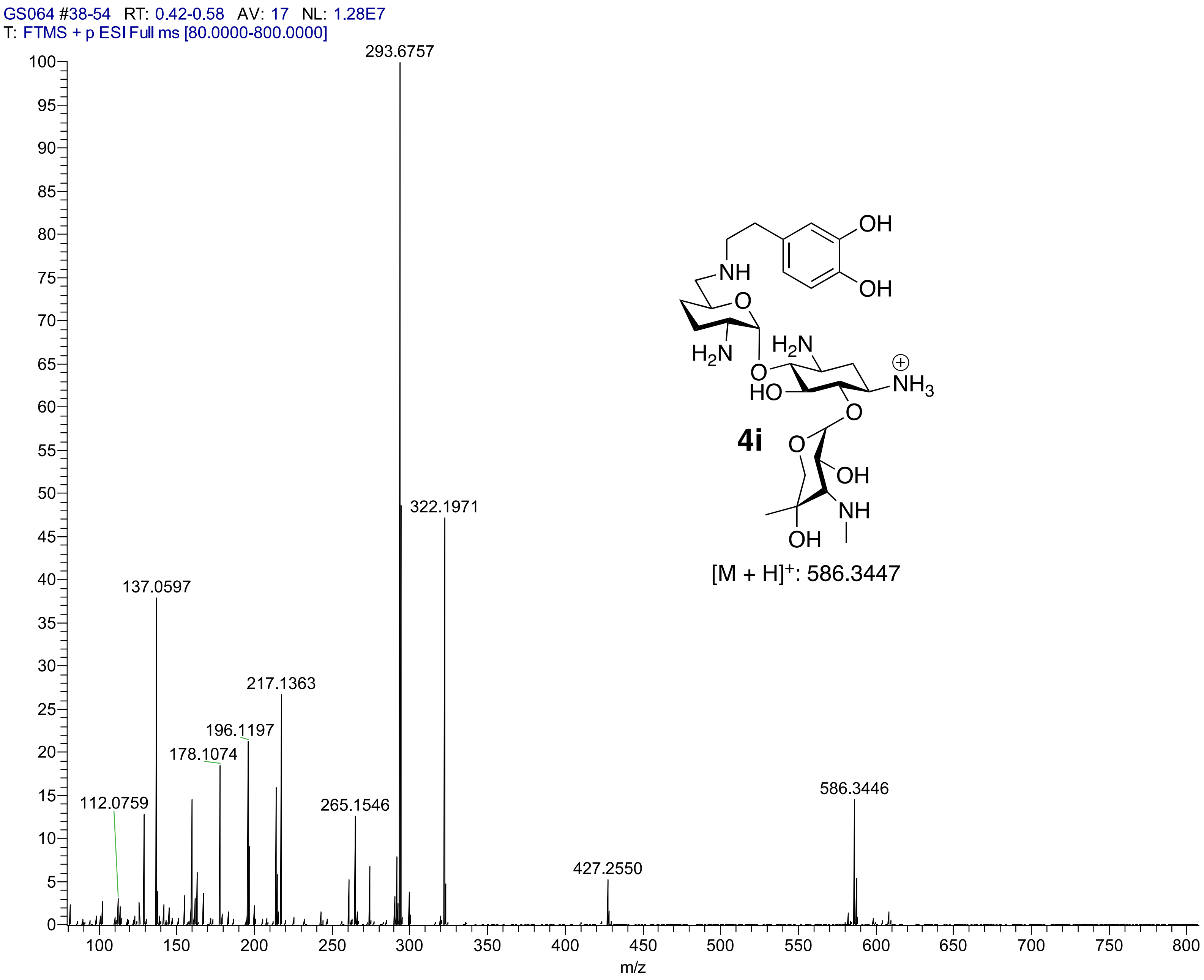

Figure S45: HRMS analysis of 6’-*N*-(3,4-Dihydroxyphenethyl)-gentamicin C1a (**4i**).

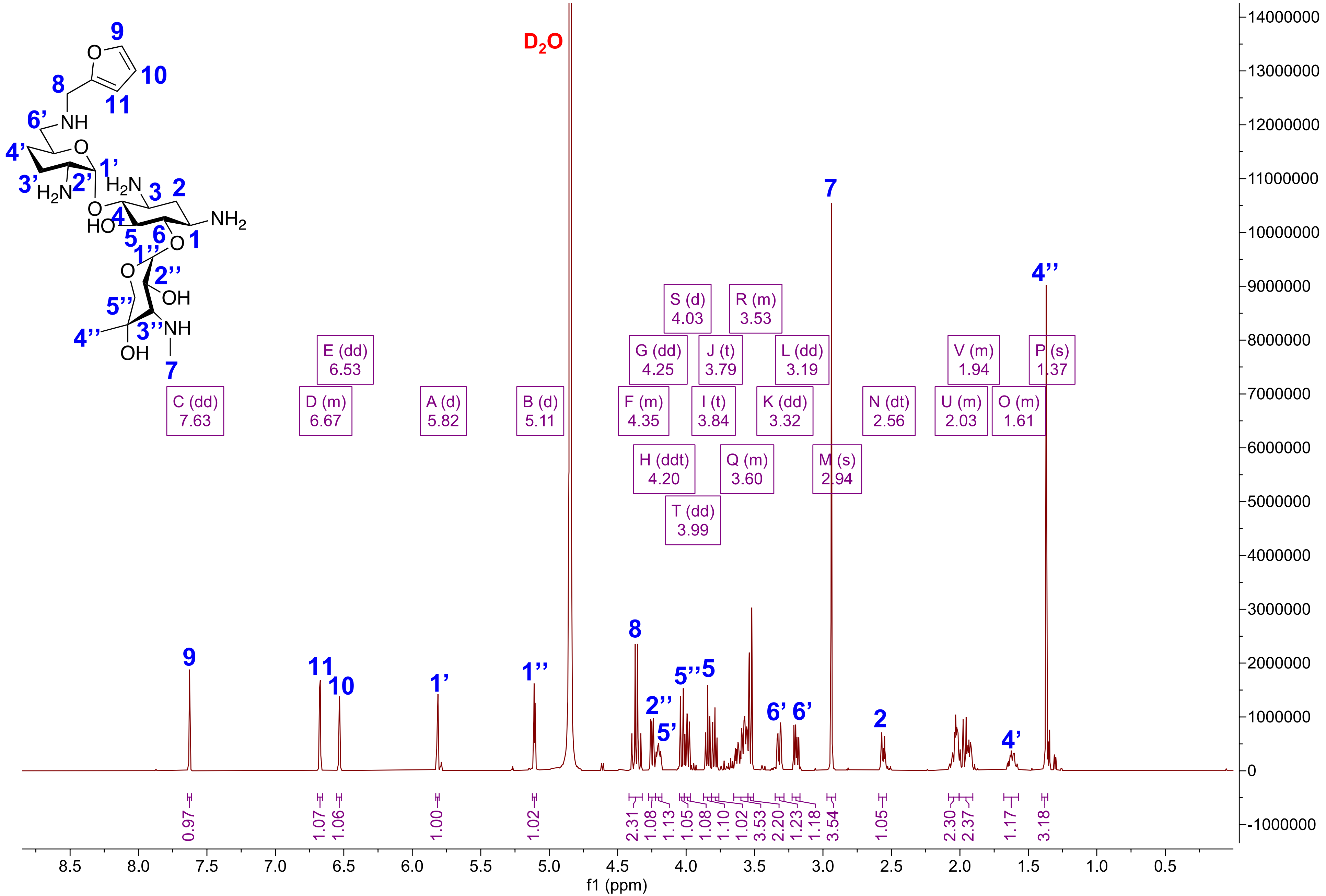

Figure S46: ^{1}H NMR spectroscopic analysis of 6’-*N*-(Furfuryl)-gentamicin C1a (**4j**).

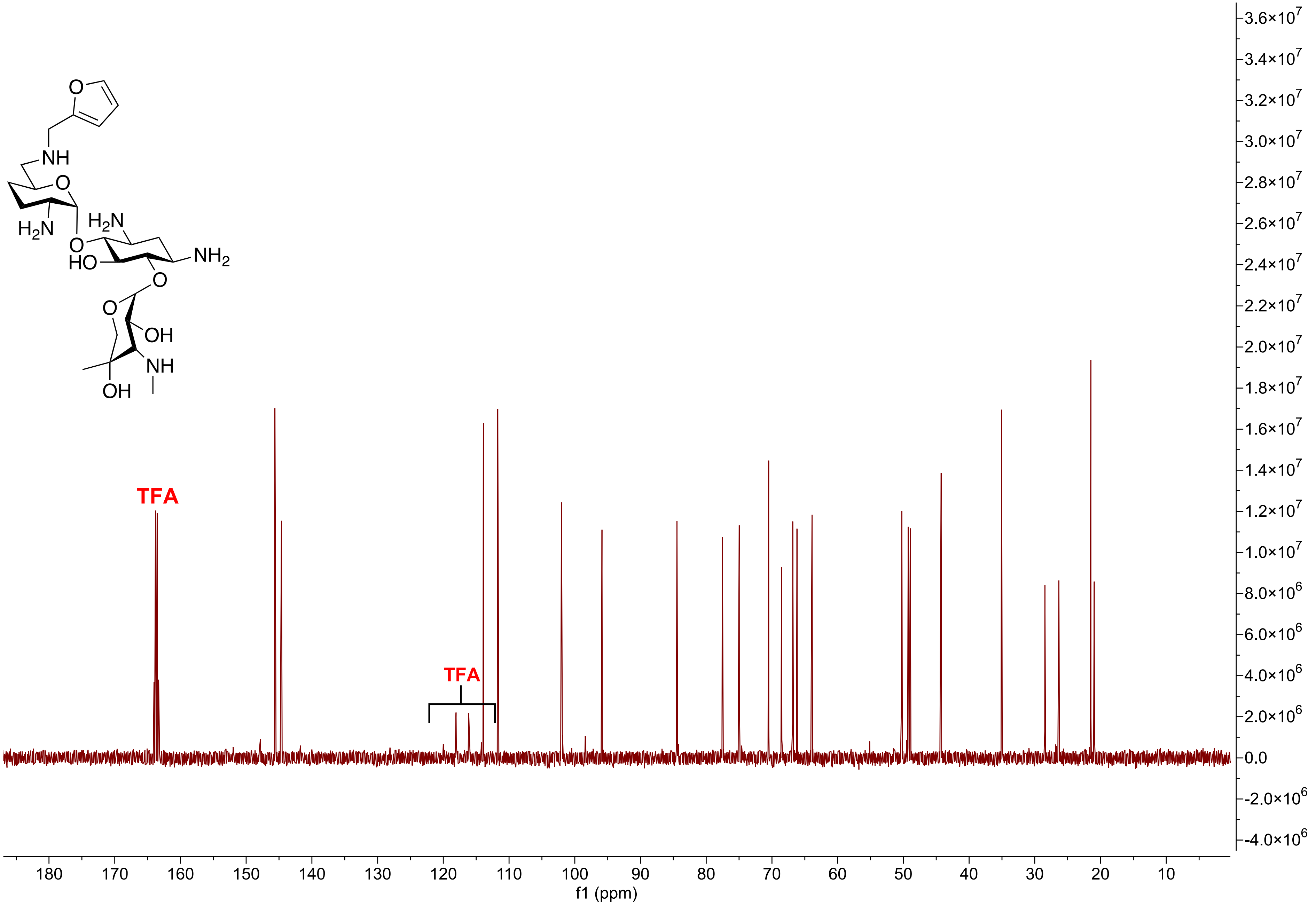

Figure S47: ^{13}C NMR spectroscopic analysis of 6'-*N*-(Furfuryl)-gentamicin C1a (**4j**).

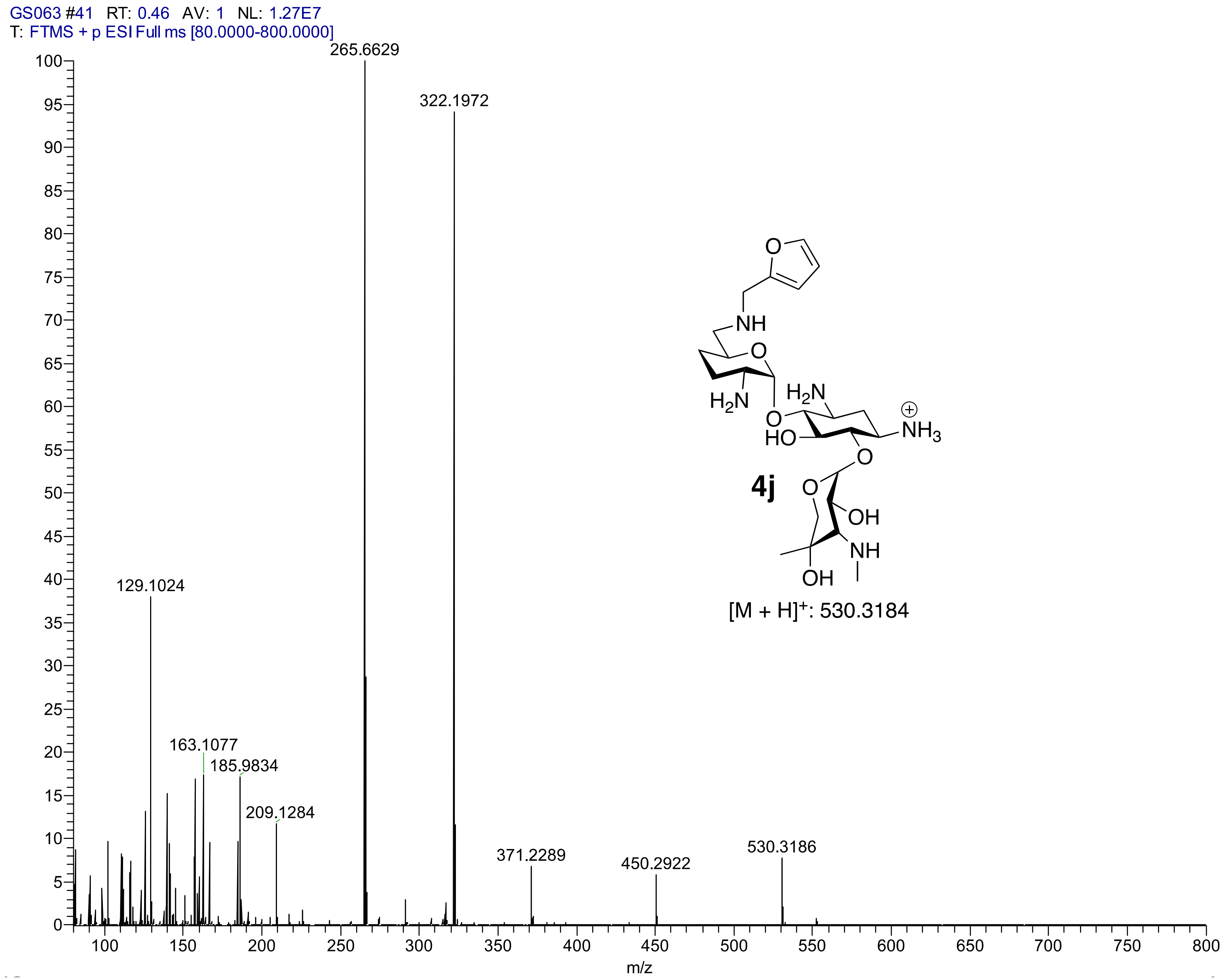

Figure S48: HRMS analysis of 6'-*N*-(Furfuryl)-gentamicin C1a (**4j**).

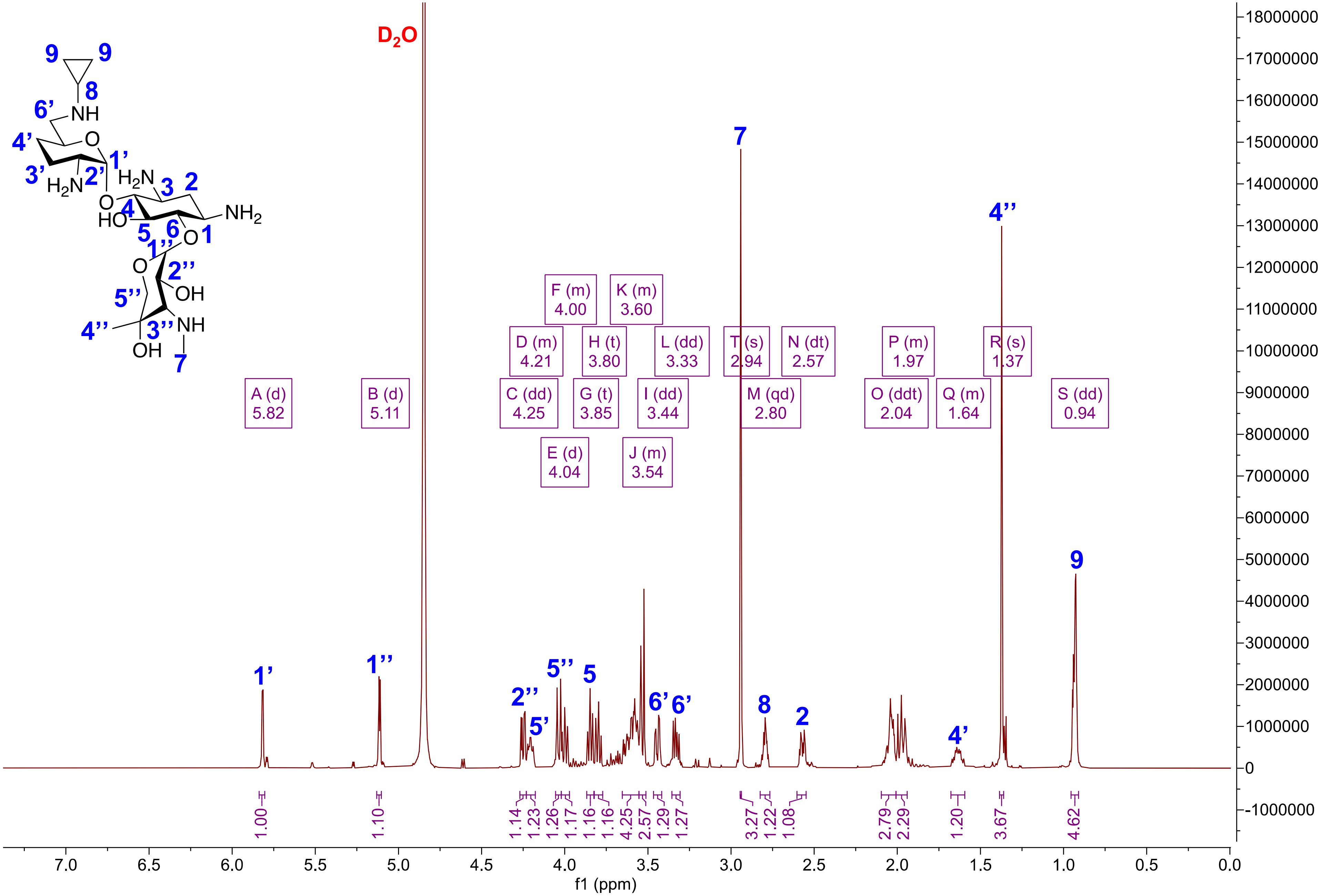

Figure S49: ^{1}H NMR spectroscopic analysis of 6'-*N*-(Cyclopropyl)-gentamicin C1a (**4k**).

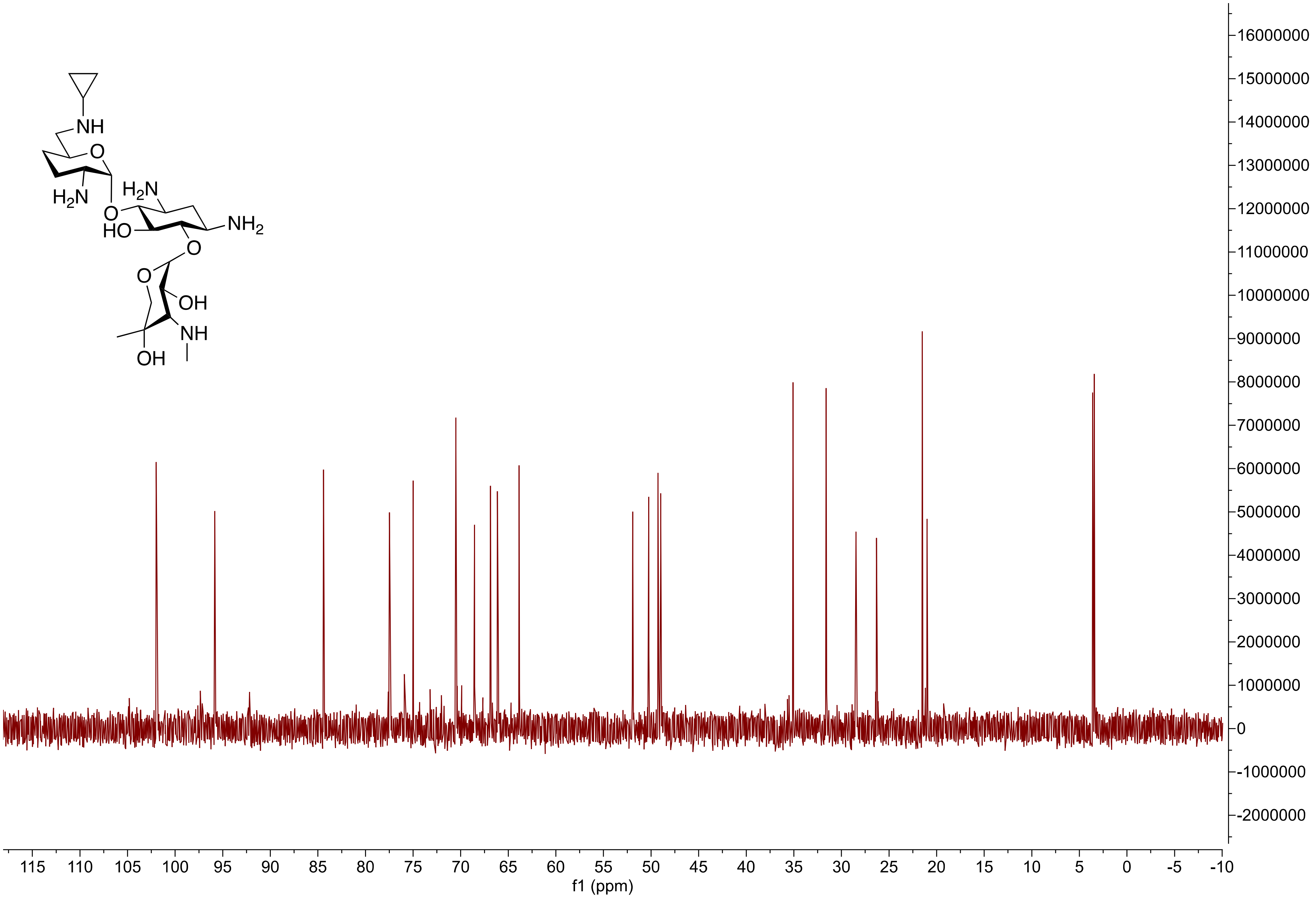

Figure S50: ^{13}C NMR spectroscopic analysis of 6'-*N*-(Cyclopropyl)-gentamicin C1a (**4k**).

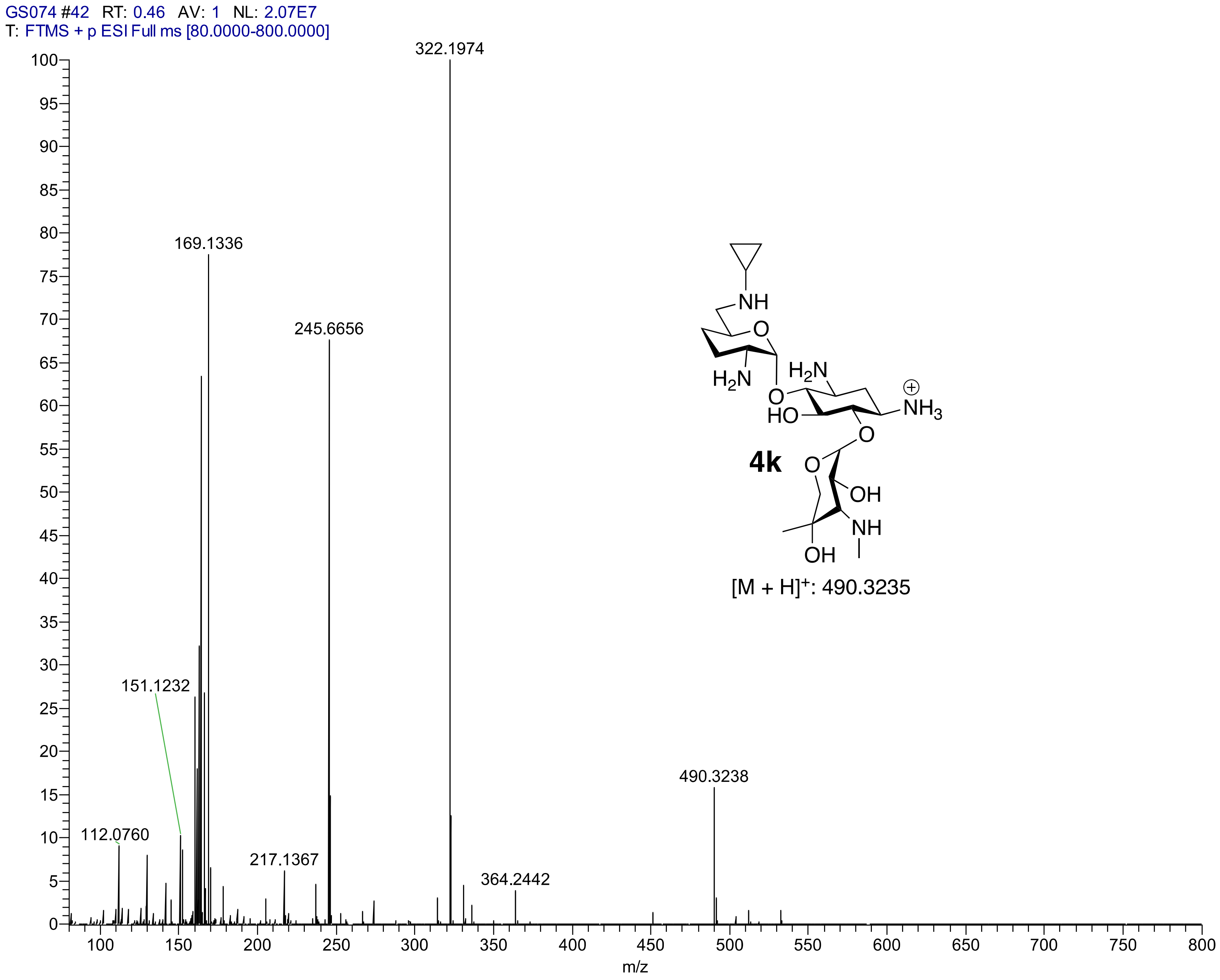

Figure S51: HRMS analysis of 6'-*N*-(Cyclopropyl)-gentamicin C1a (**4k**).

7. LC-MS analysis of reductive amination products

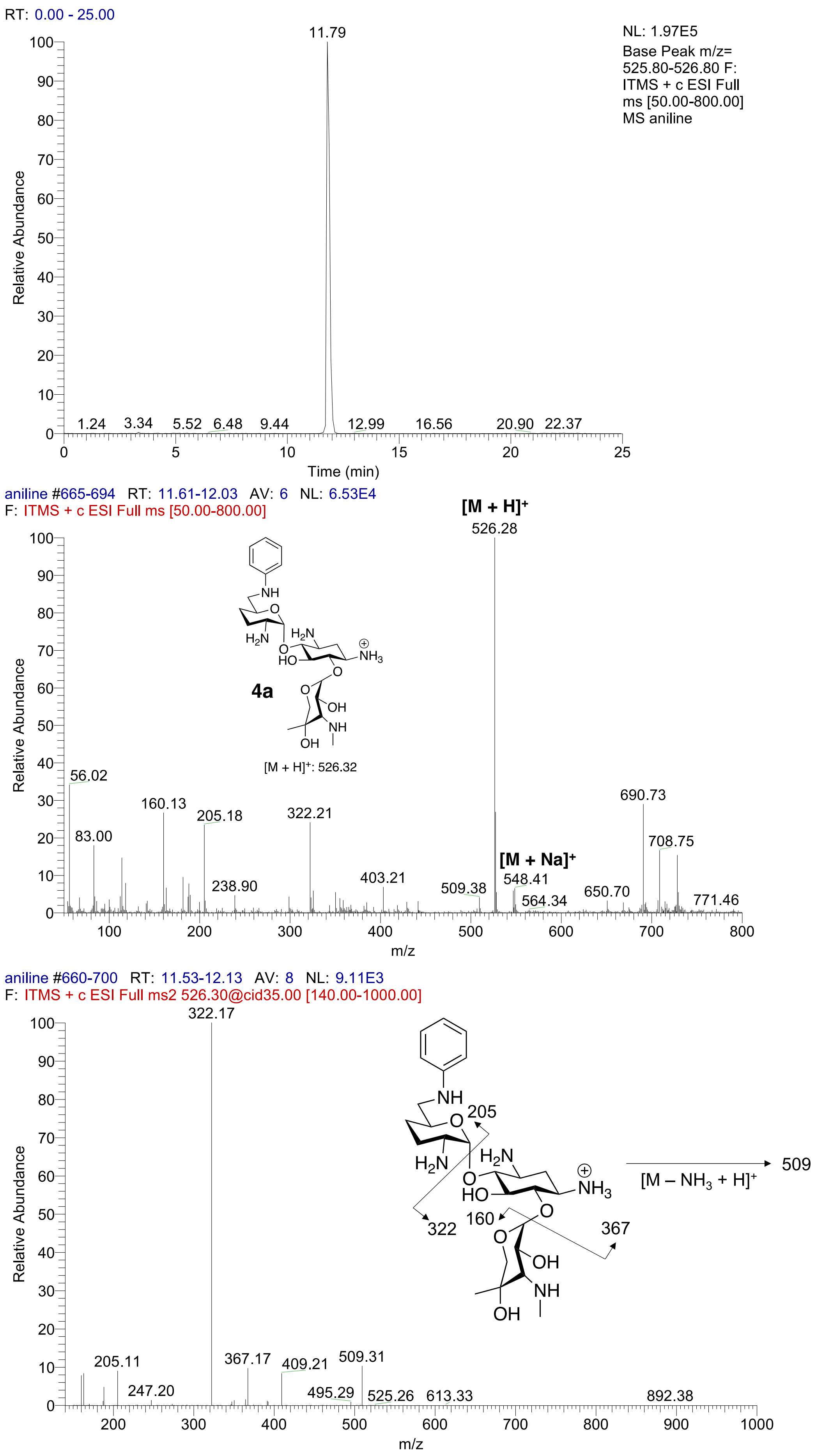

Figure S52: LC-MS analysis of 6'-*N*-(Phenyl)-gentamicin C1a (**4a**).

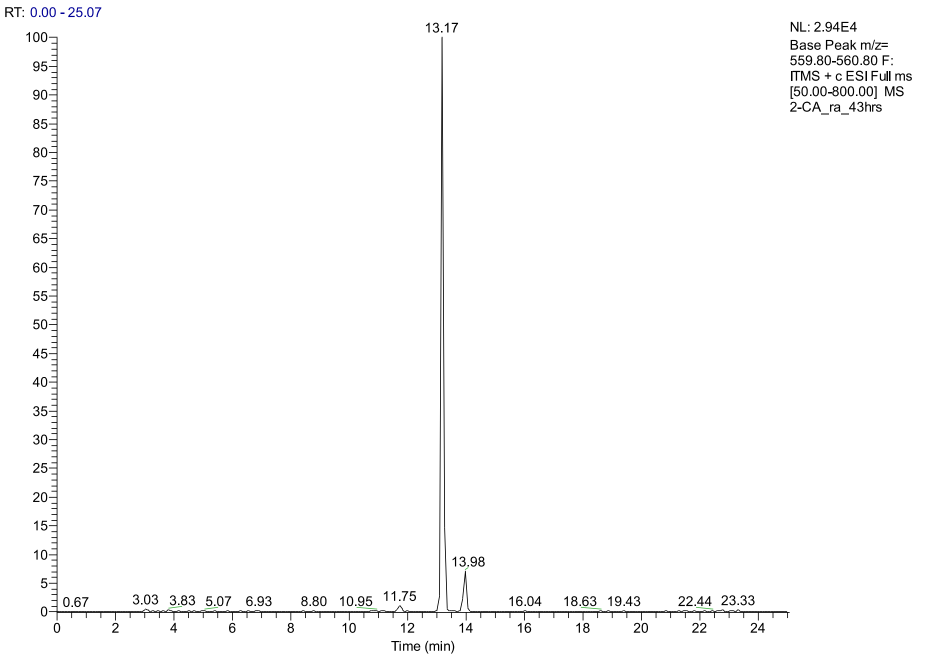

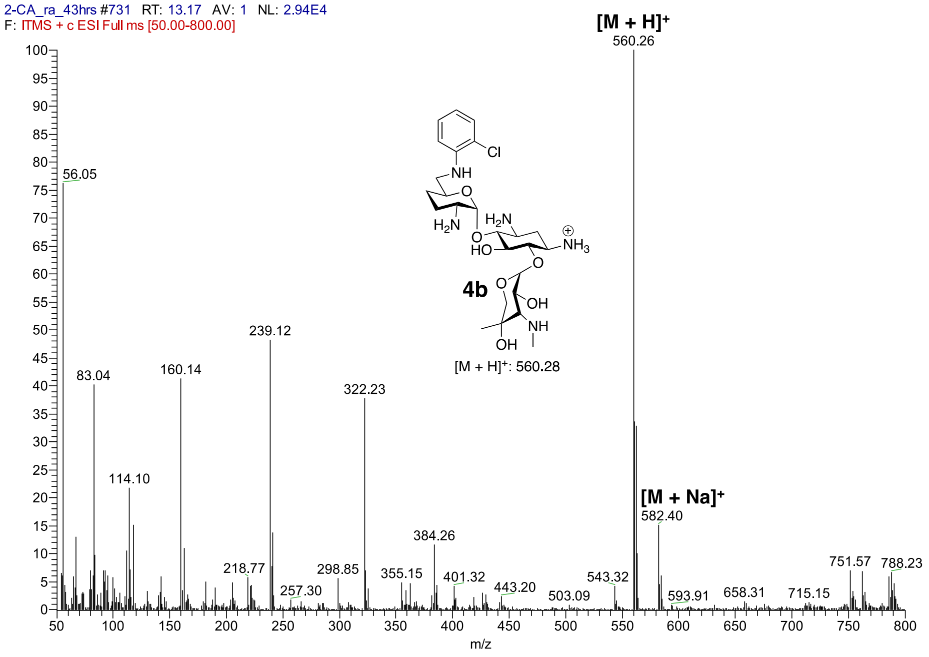

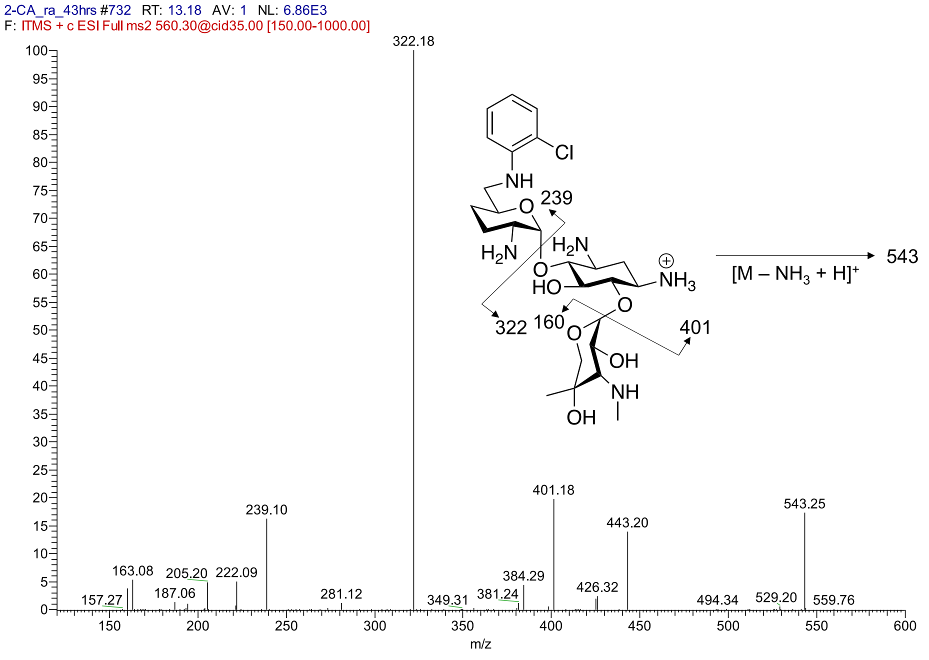

Figure S53: LC-MS analysis of 6'-*N*-(2-Chlorophenyl)-gentamicin C1a (**4b**).

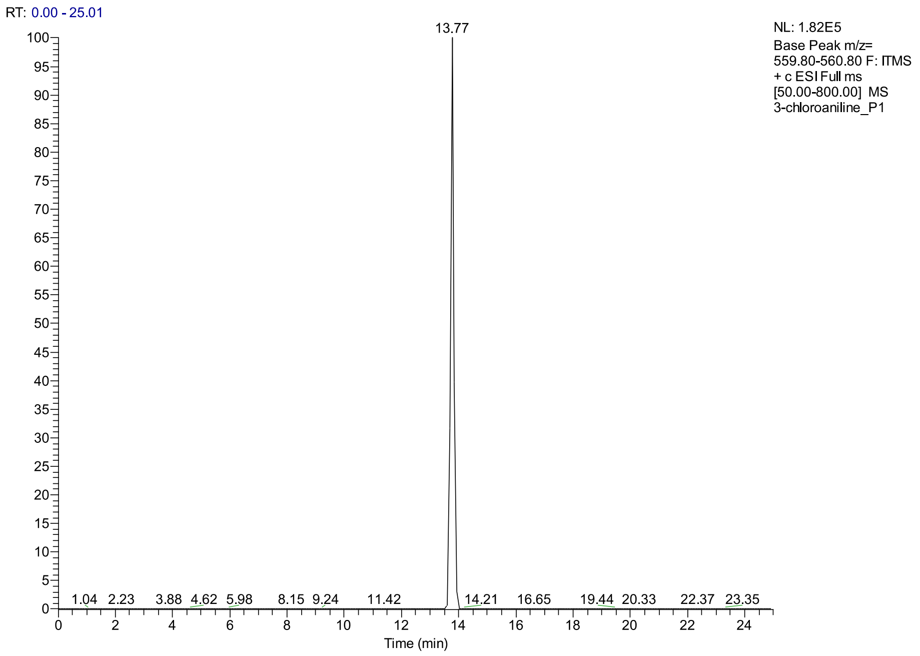

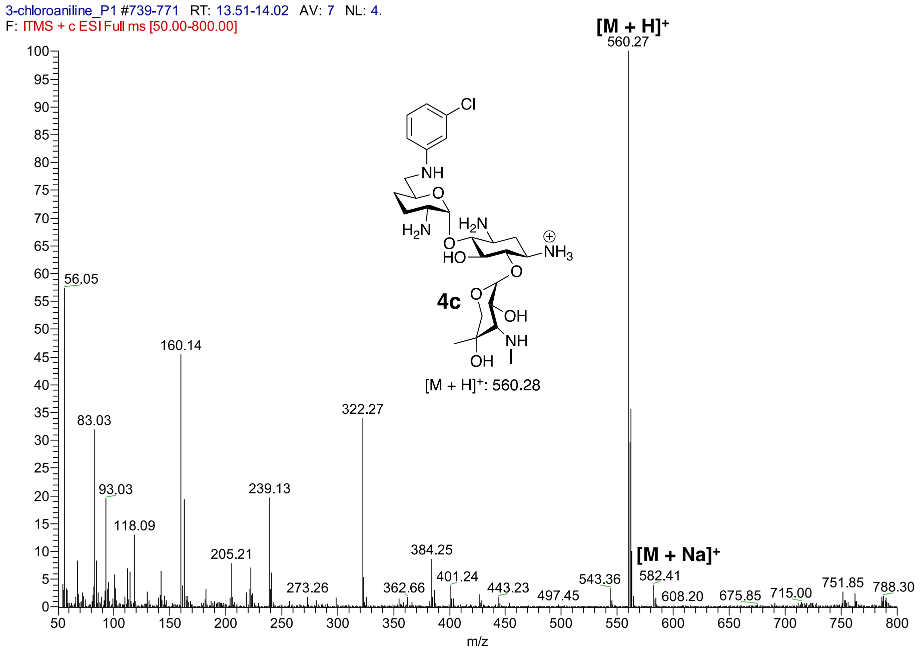

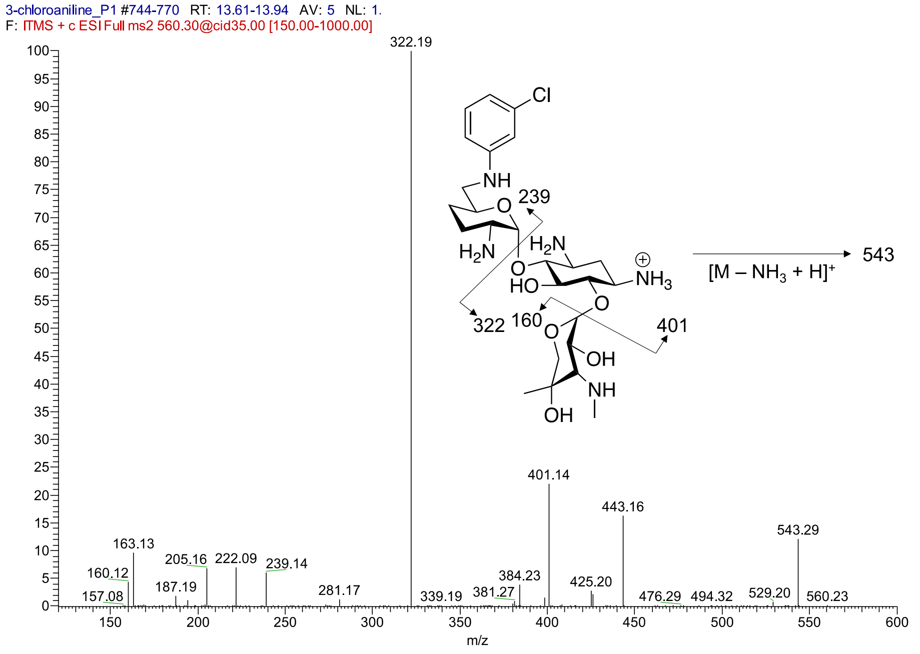

Figure S54: LC-MS analysis of 6’-*N*-(3-Chlorophenyl)-gentamicin C1a (**4c**).

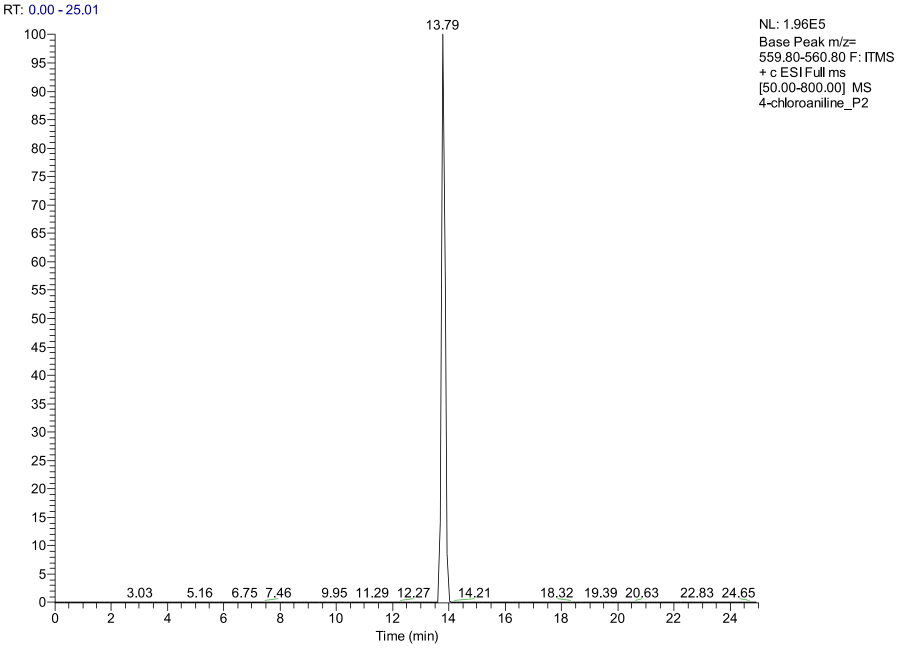

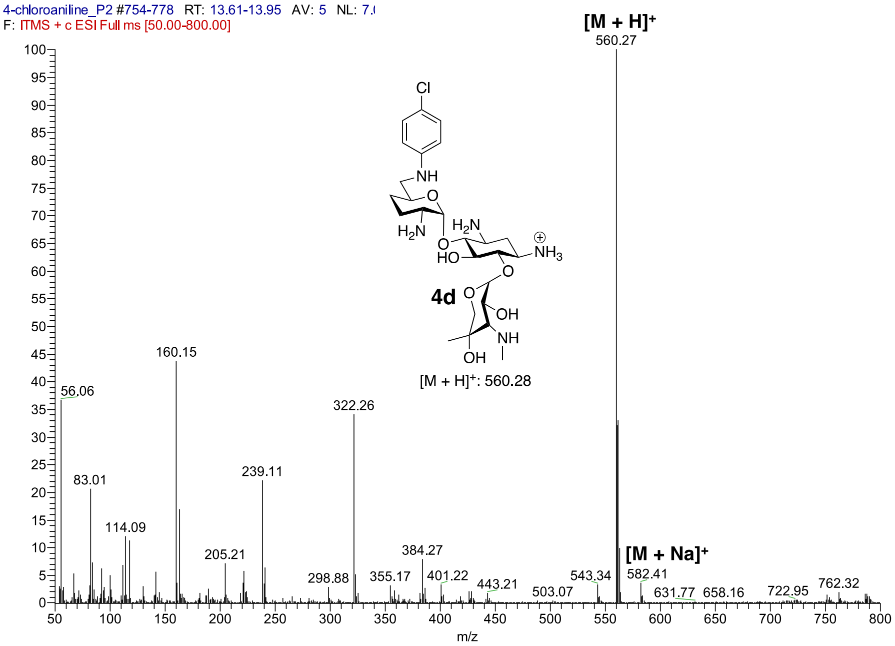

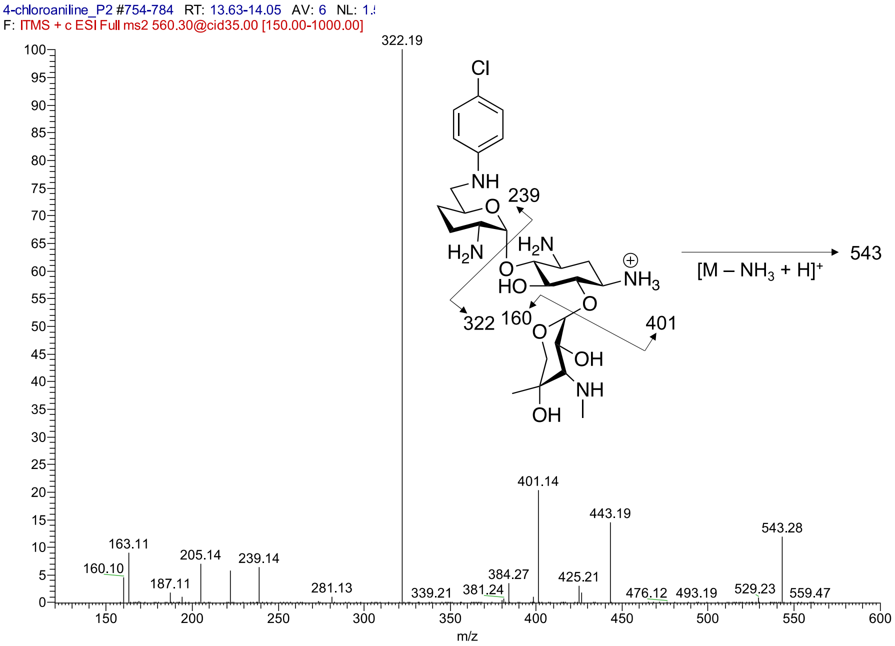

Figure S55: LC-MS analysis of 6’-*N*-(4-Chlorophenyl)-gentamicin C1a (**4d**).

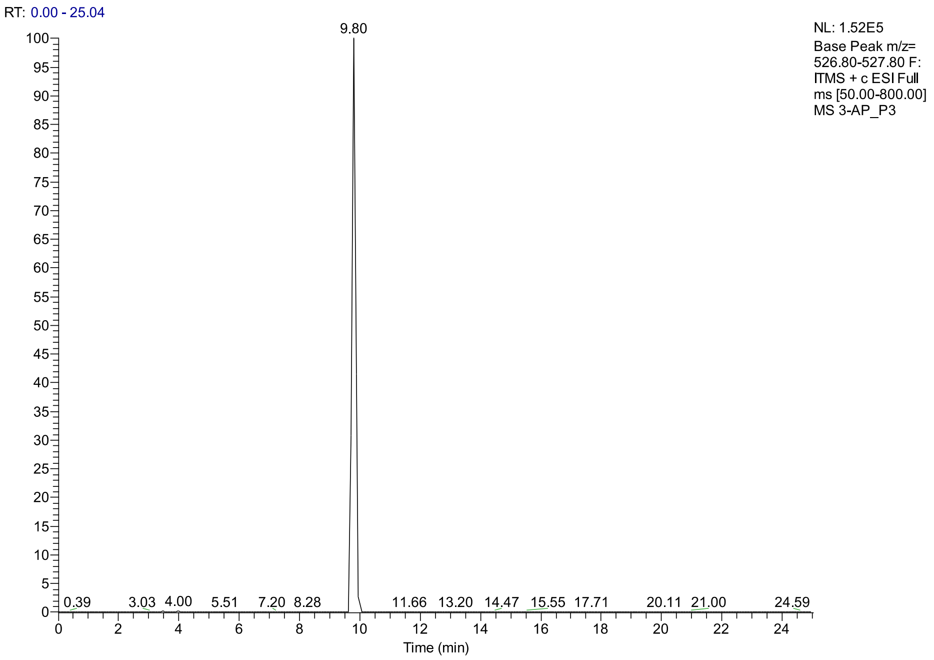

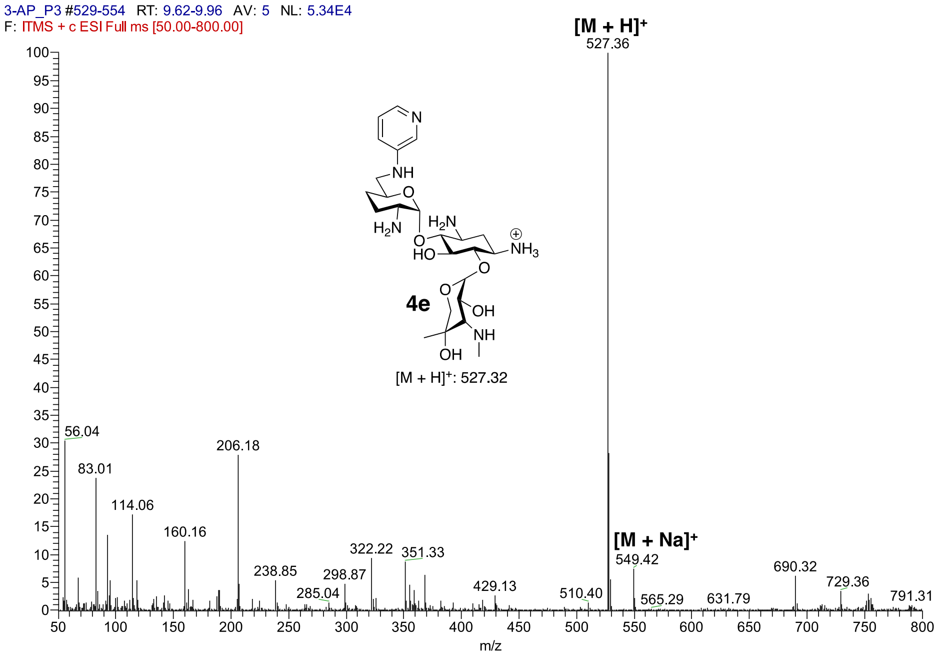

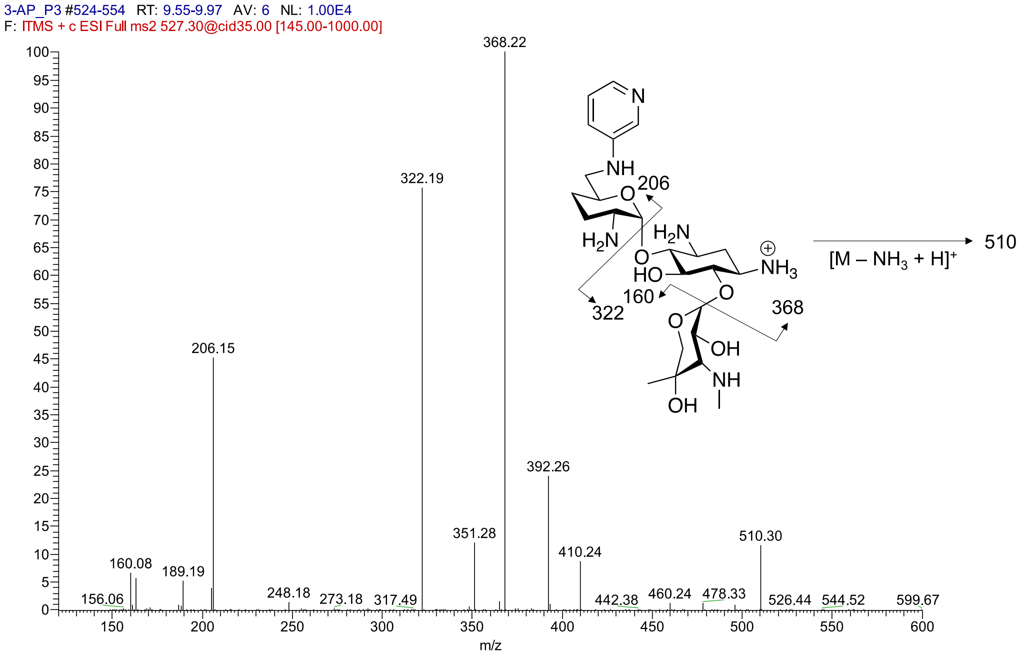

Figure S56: LC-MS analysis of 6’-*N*-(Pyridin-3-ylamino)-gentamicin C1a (**4e**).

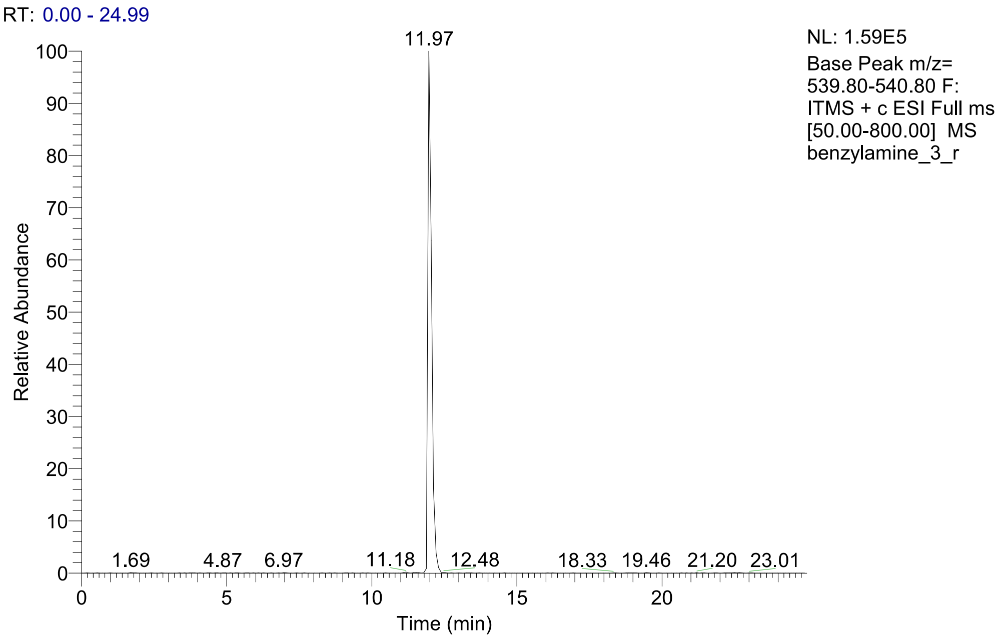

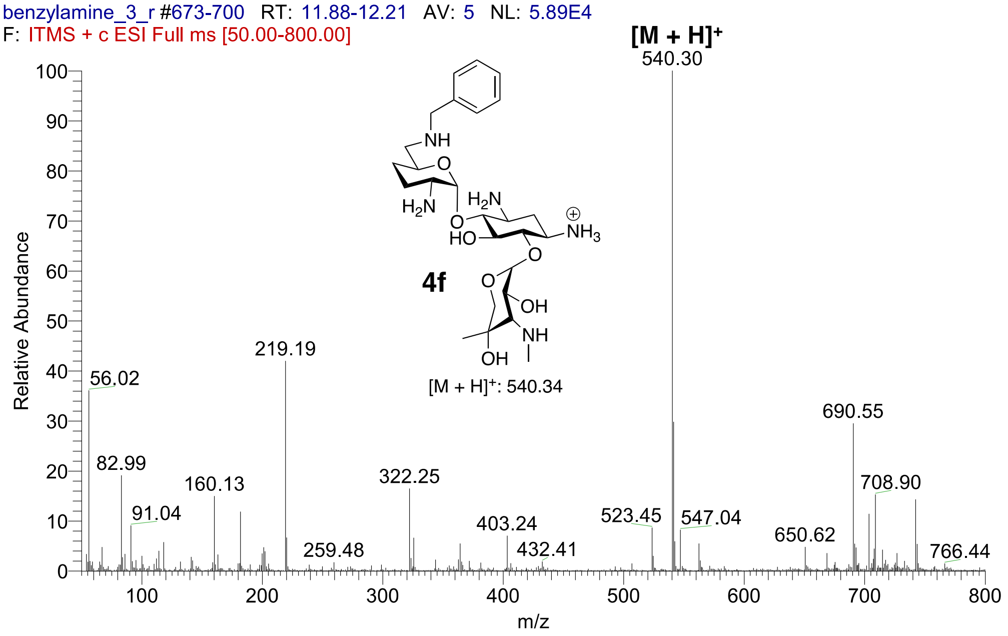

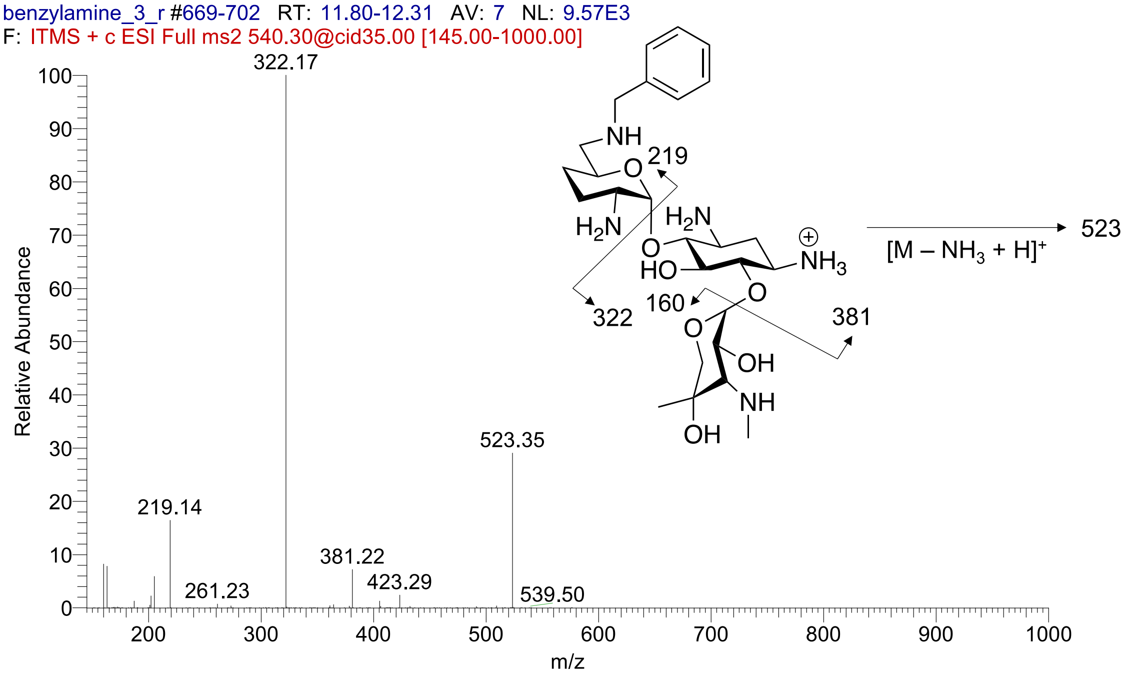

Figure S57: LC-MS analysis of 6'-*N*-(Benzyl)-gentamicin C1a (**4f**).

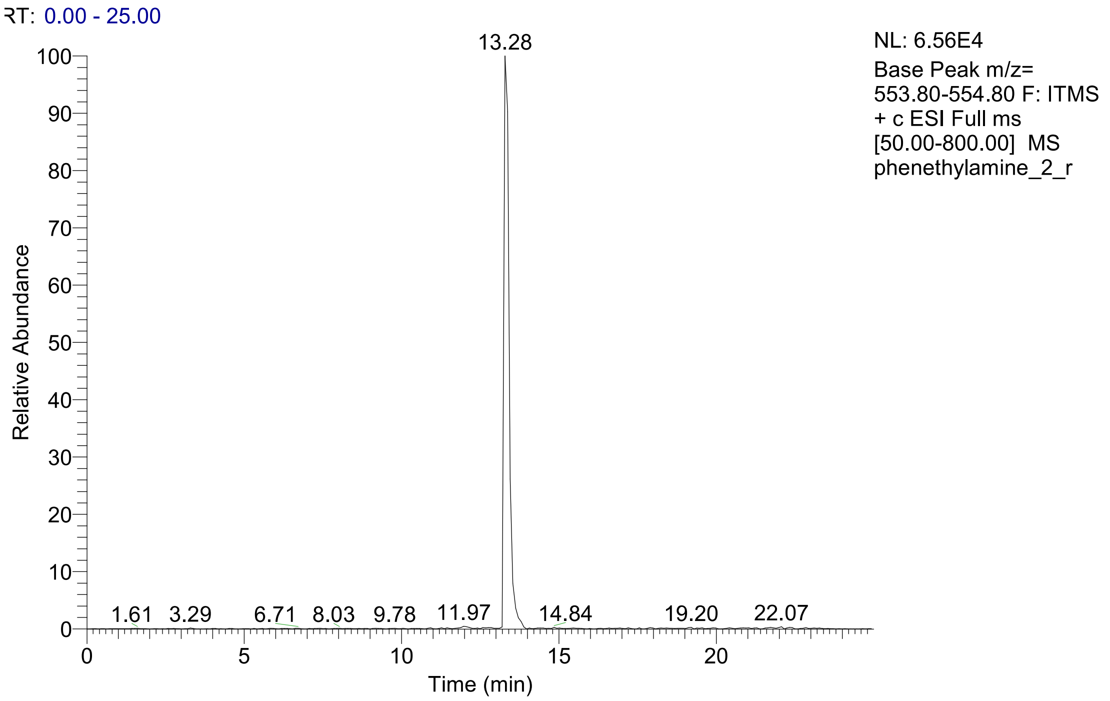

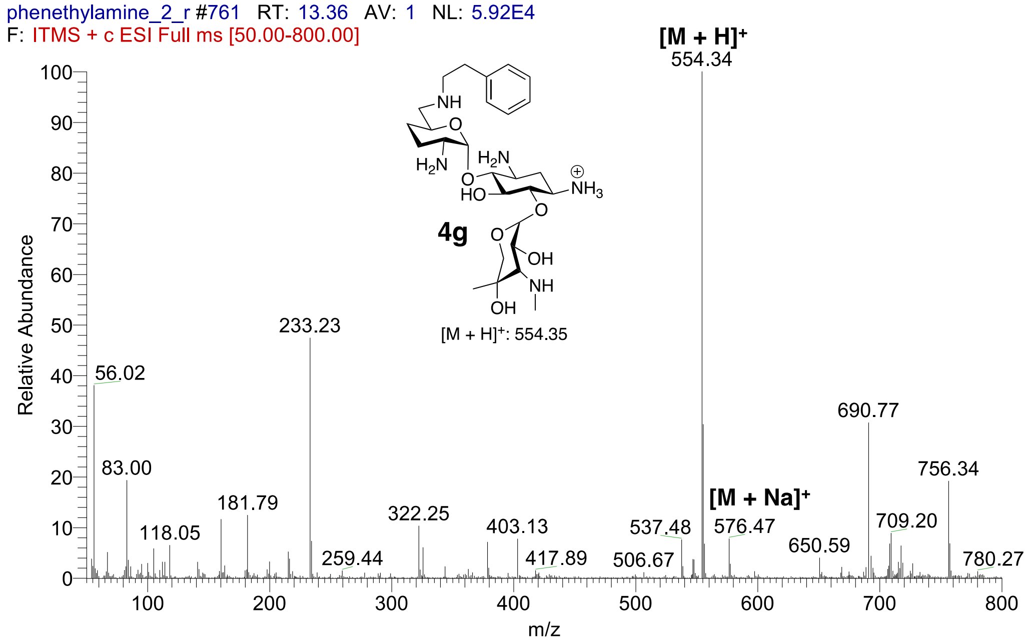

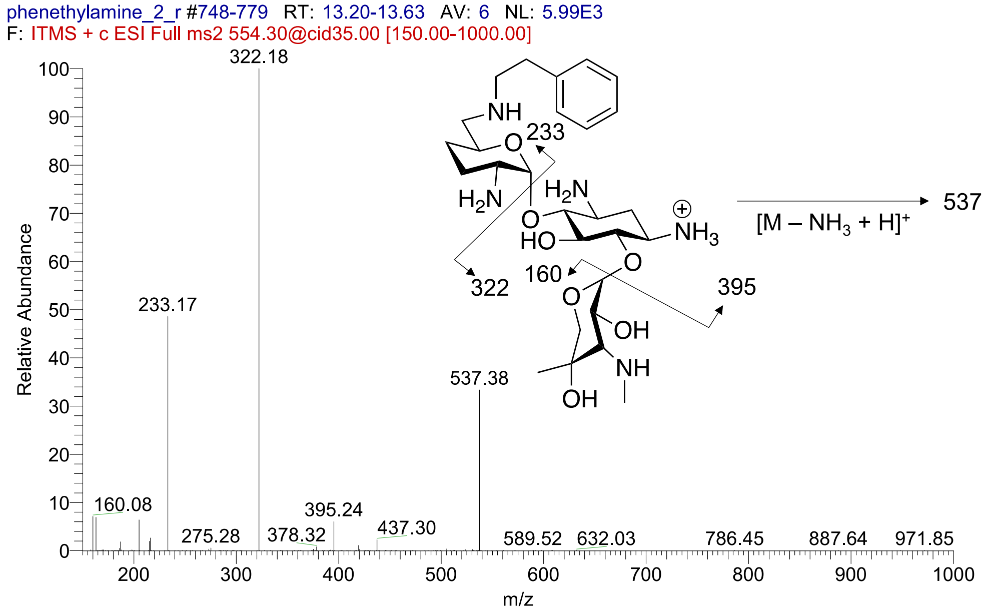

Figure S58: LC-MS analysis of 6'-*N*-(Phenethyl)-gentamicin C1a (**4g**).

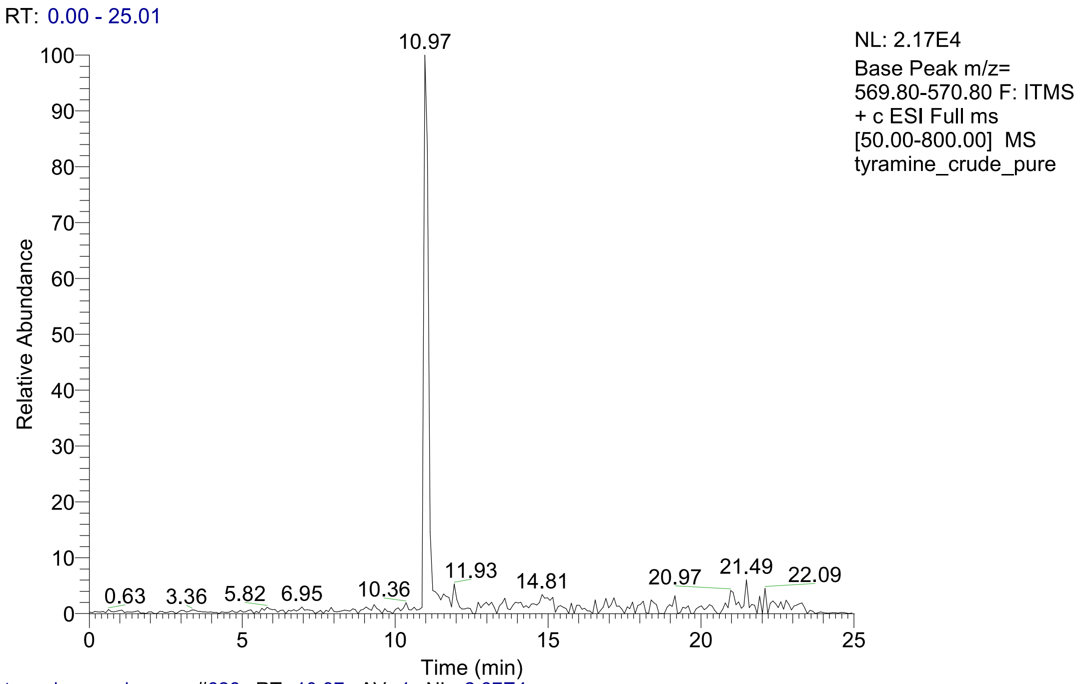

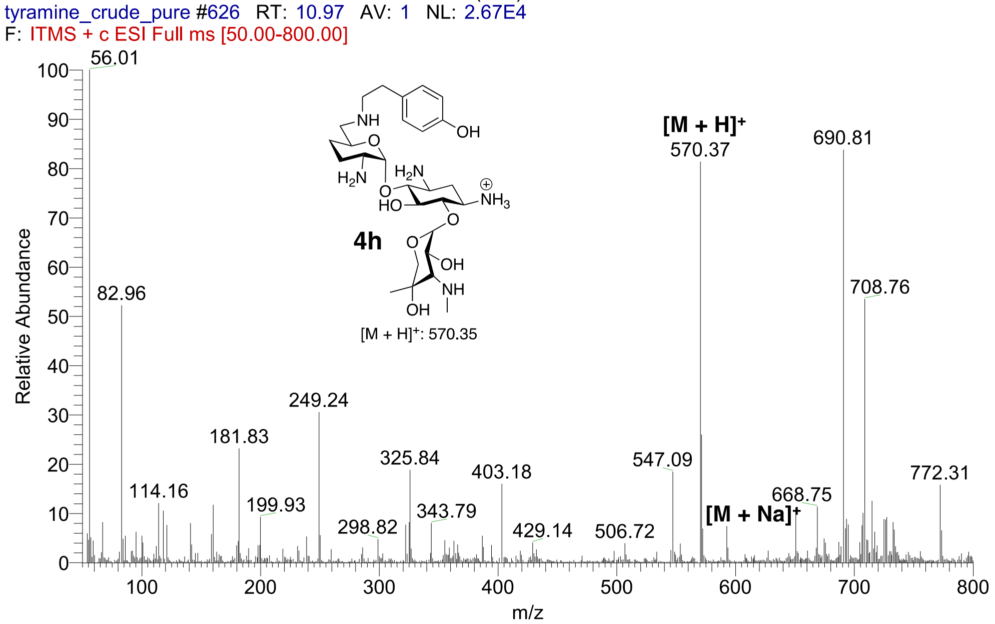

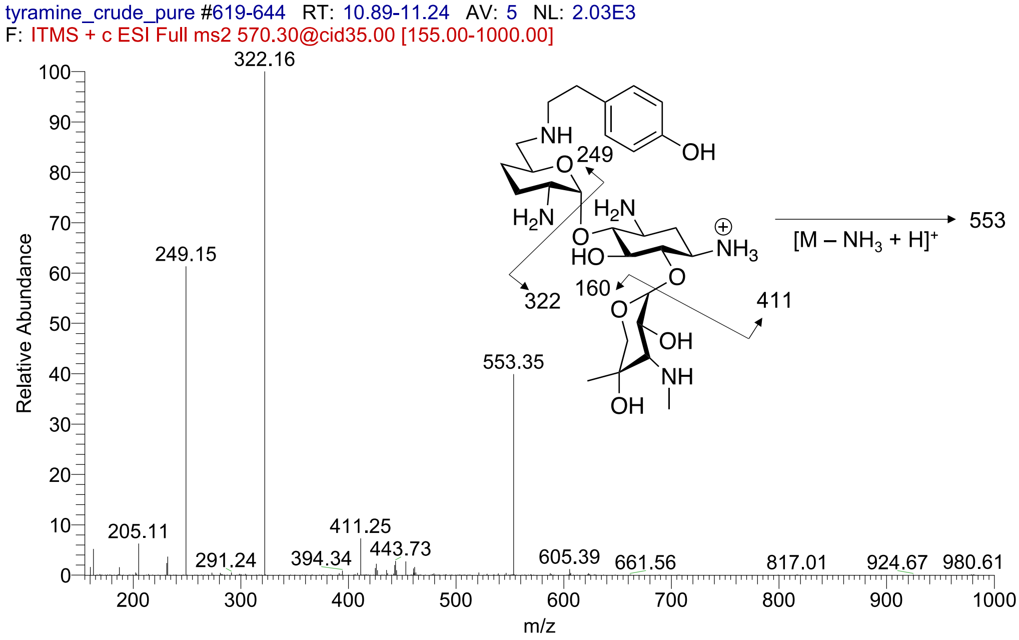

Figure S59: LC-MS analysis of 6’-*N*-(4-Hydroxyphenethyl)-gentamicin C1a (**4h**).

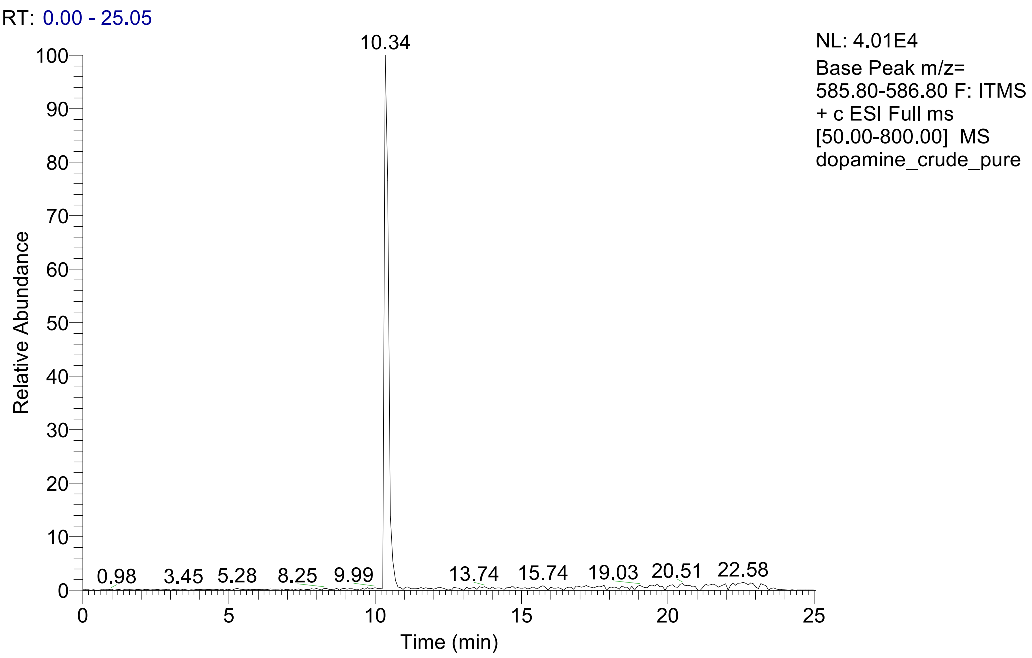

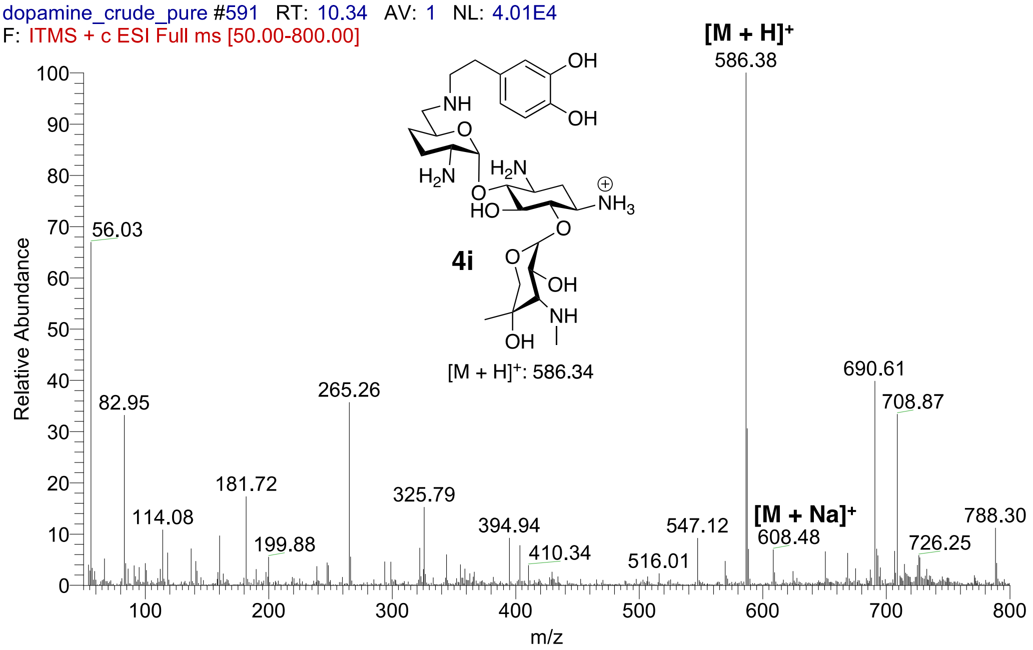

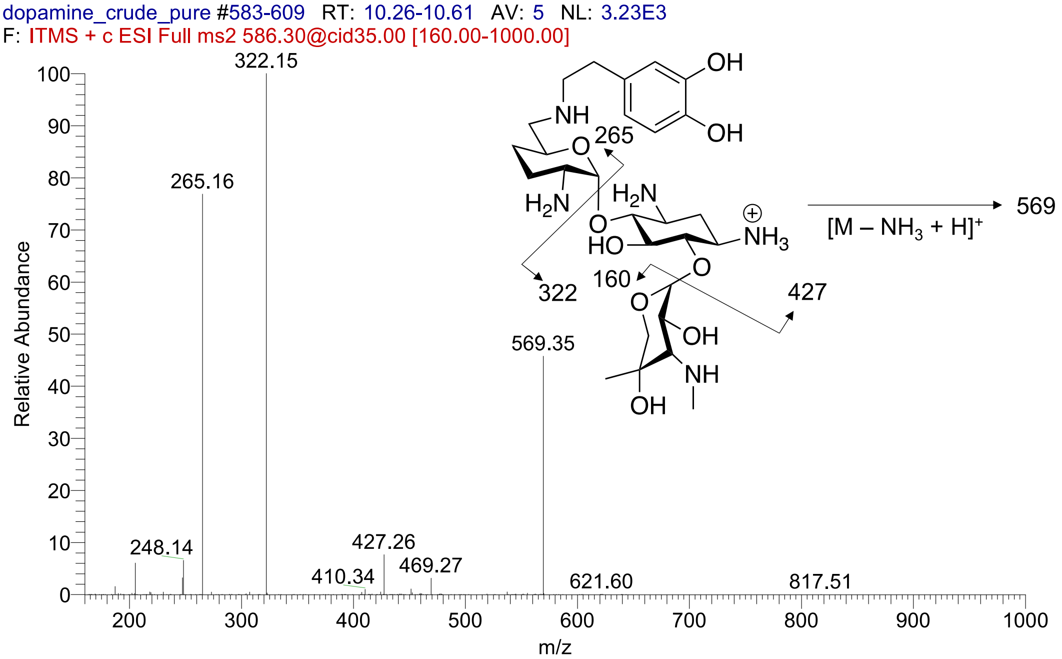

Figure S60: LC-MS analysis of 6'-*N*-(3,4-Dihydroxyphenethyl)-gentamicin C1a (**4i**).

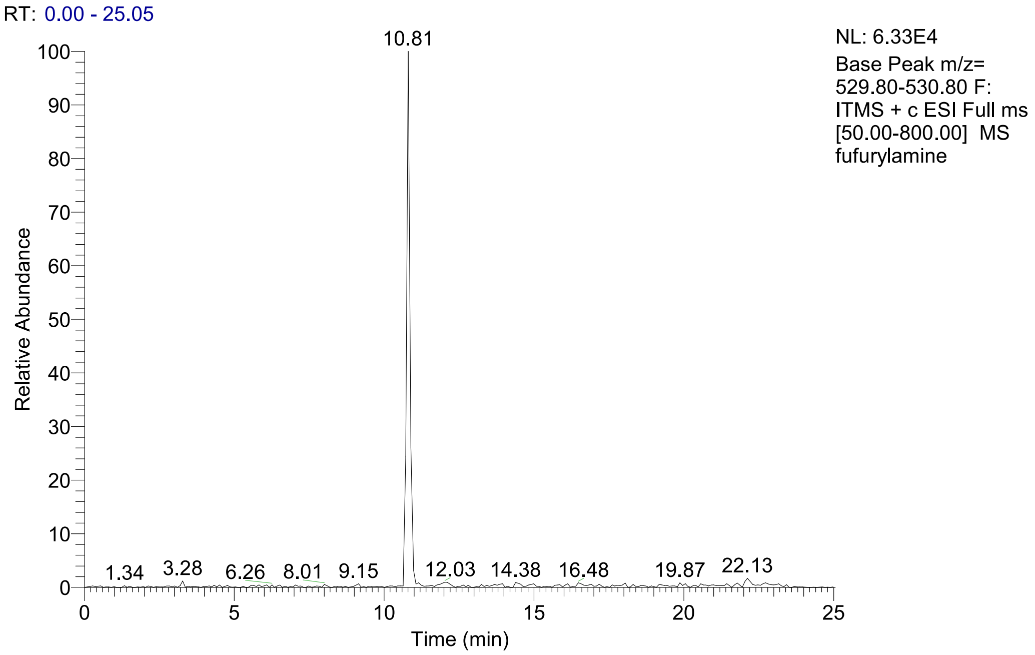

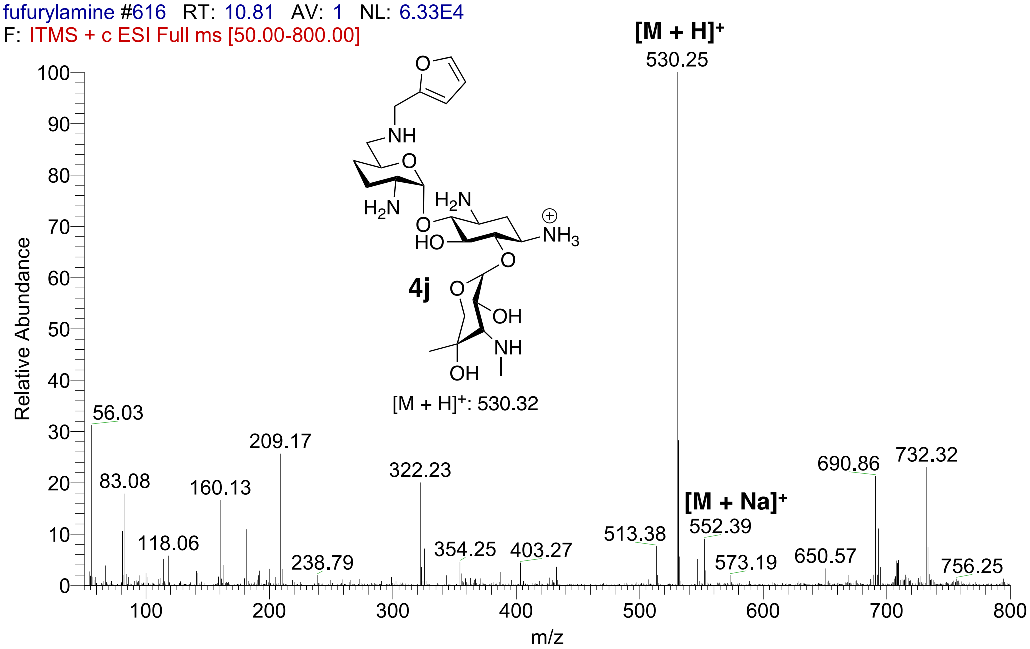

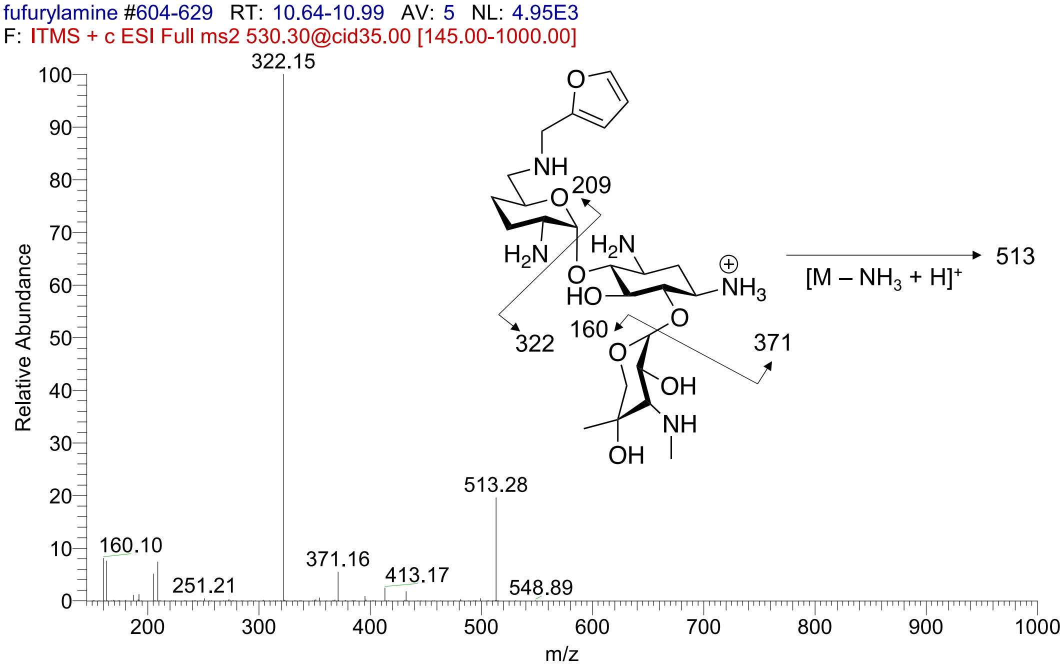

Figure S61: LC-MS analysis of 6'-*N*-(Furfuryl)-gentamicin C1a (**4j**).

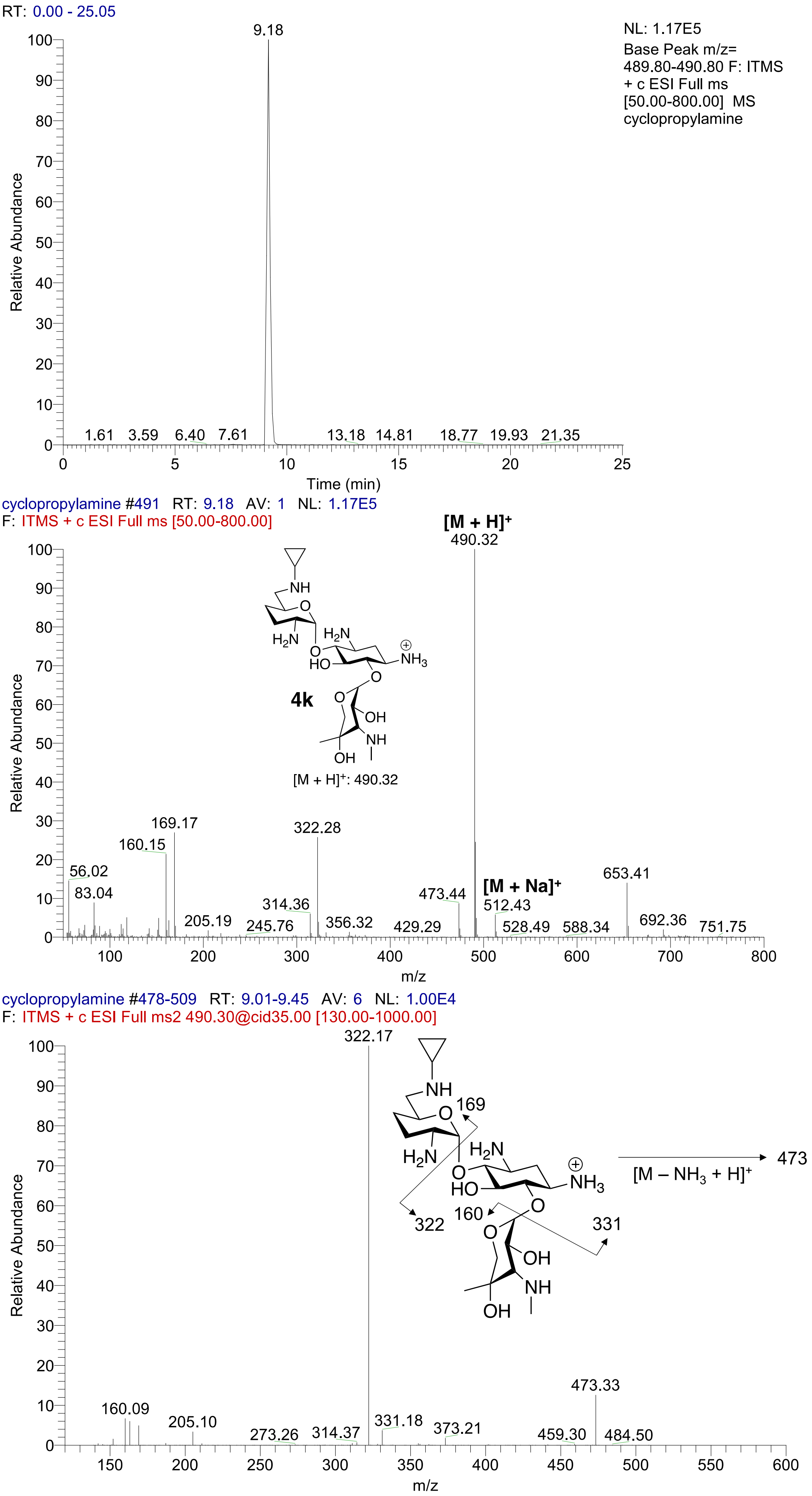

Figure S62: LC-MS analysis of 6'-*N*-(Cyclopropyl)-gentamicin C1a (**4k**).

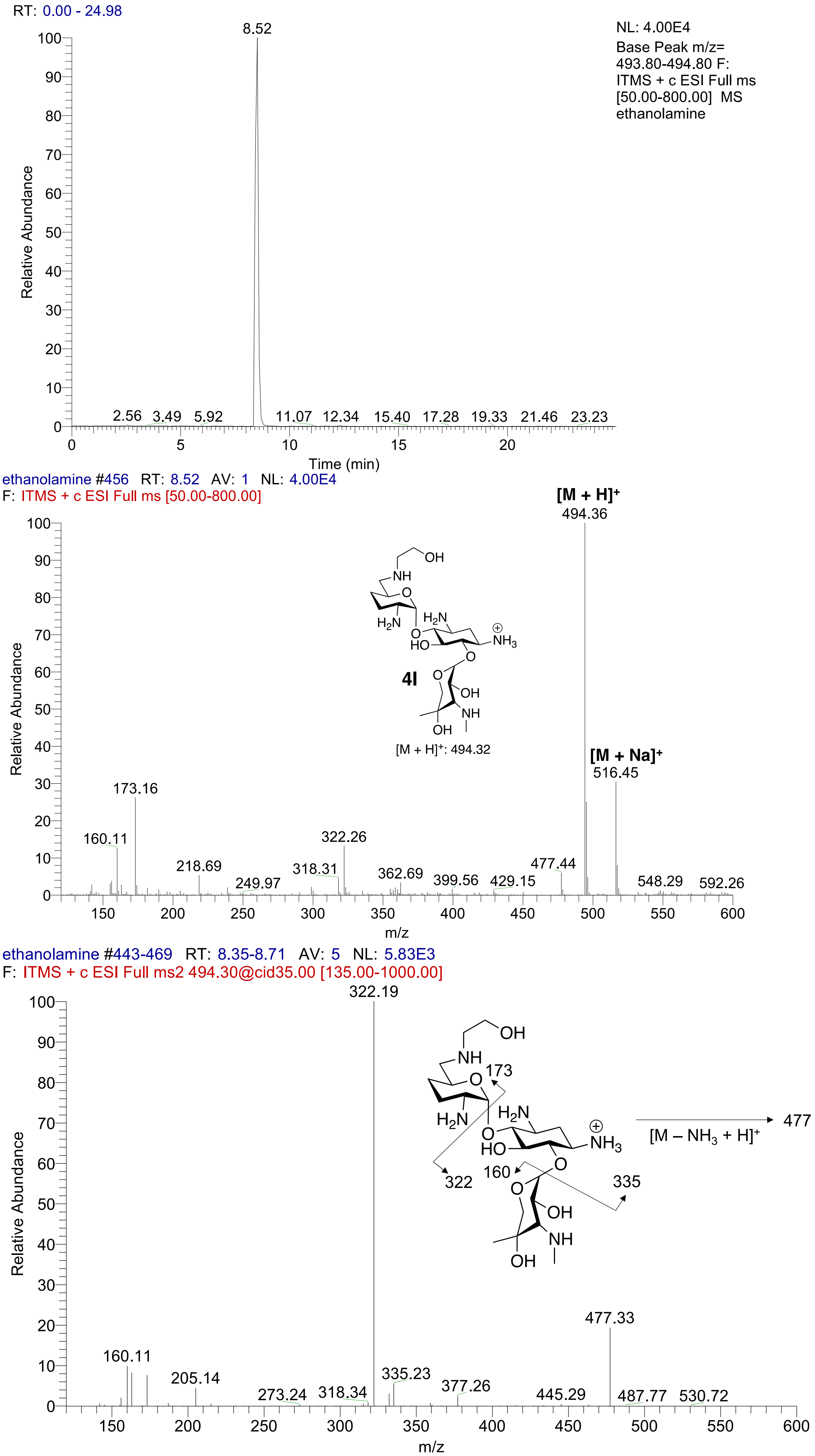

Figure S63: LC-MS analysis of 6’-*N*-(2-Hydroxyethyl)-gentamicin C1a (**4l**).

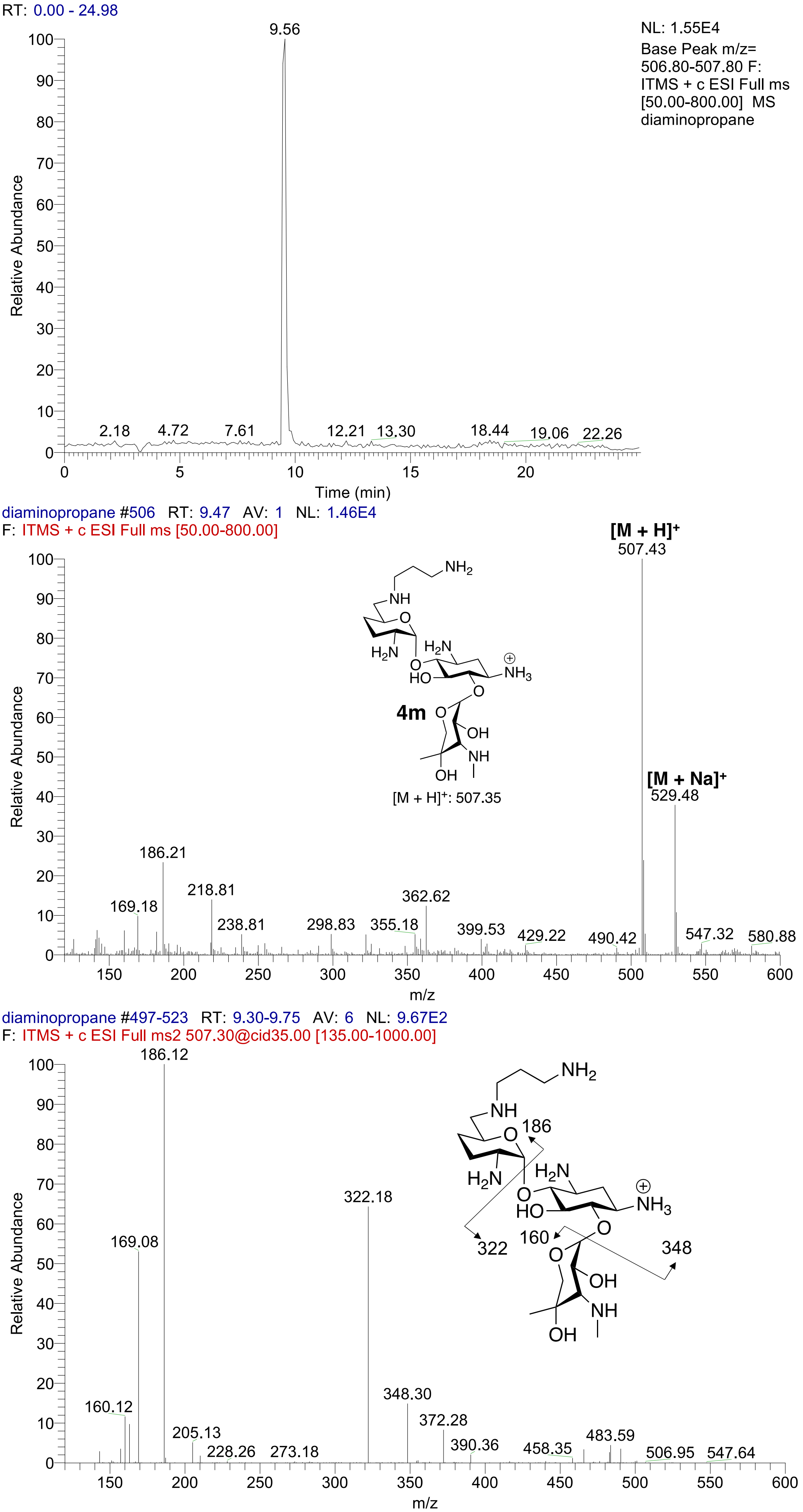

Figure S64: LC-MS analysis of 6'-*N*-(3-Aminopropyl)-gentamicin C1a (**4m**).

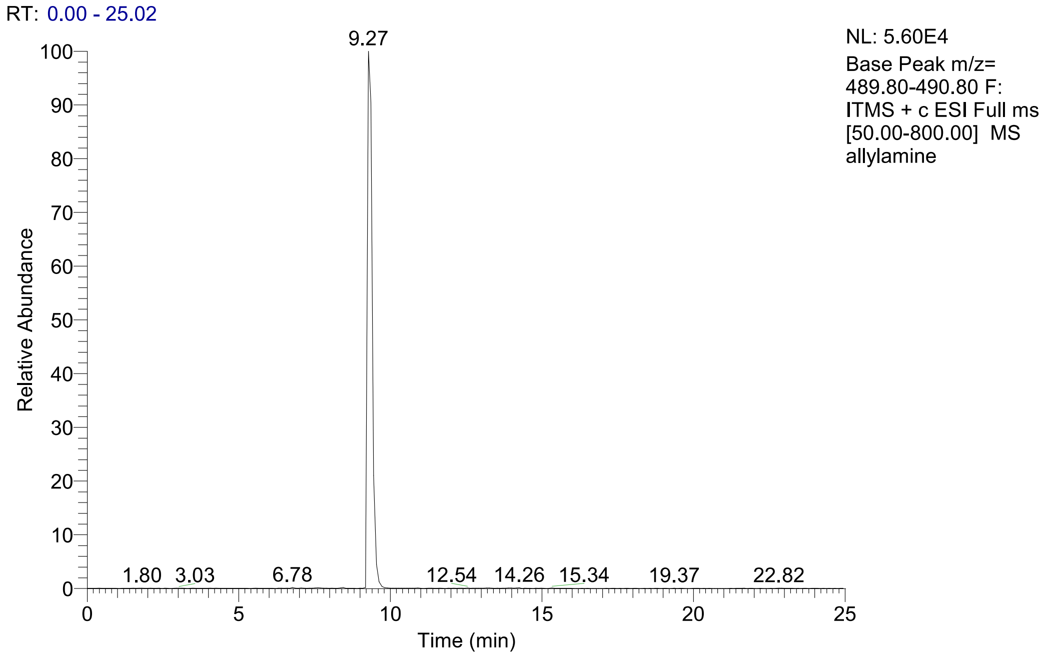

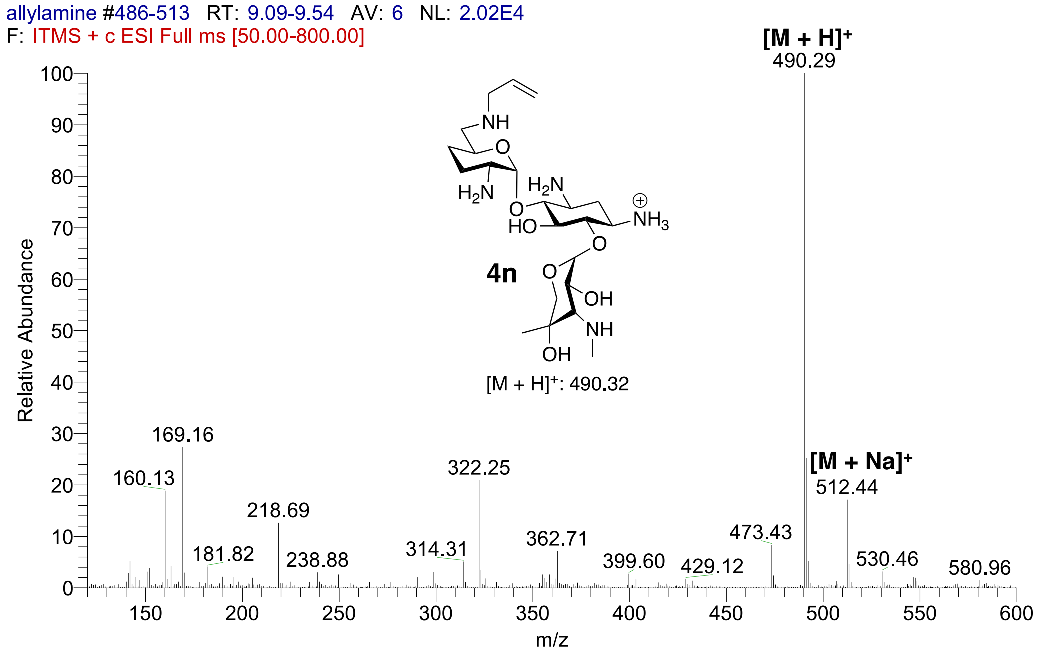

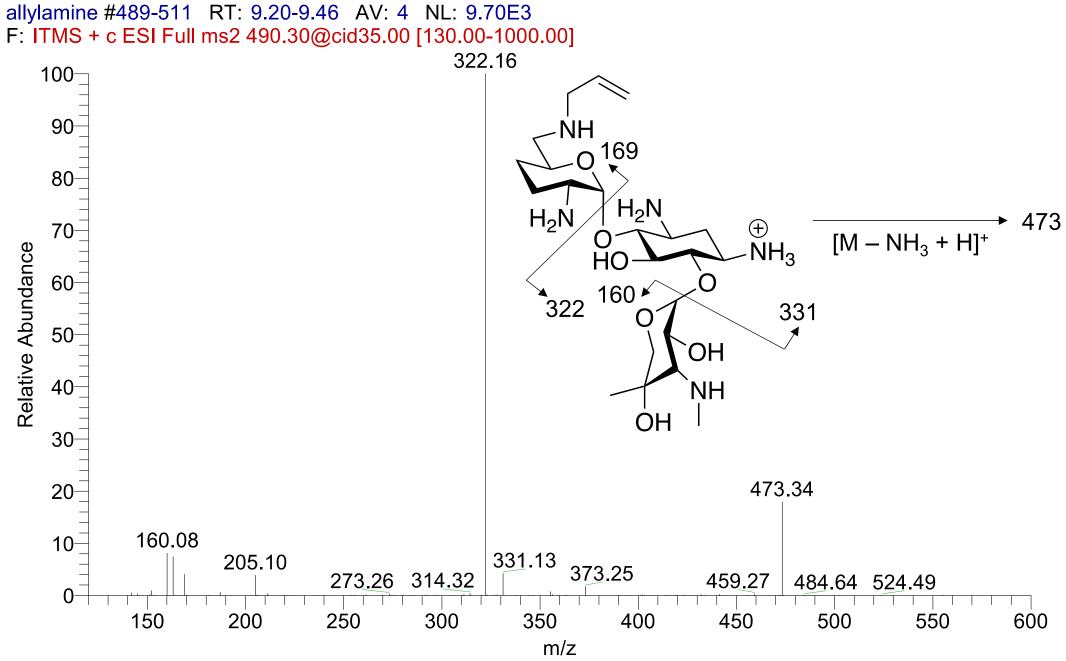

Figure S65: LC-MS analysis of 6'-*N*-(Allyl)-gentamicin C1a (**4n**).

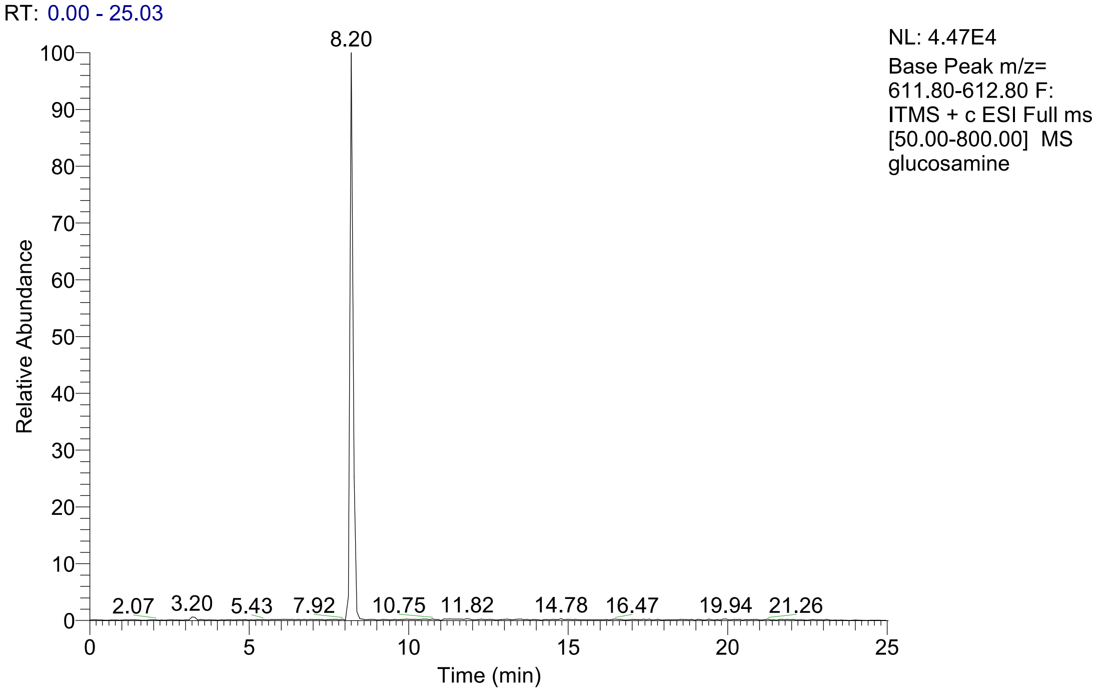

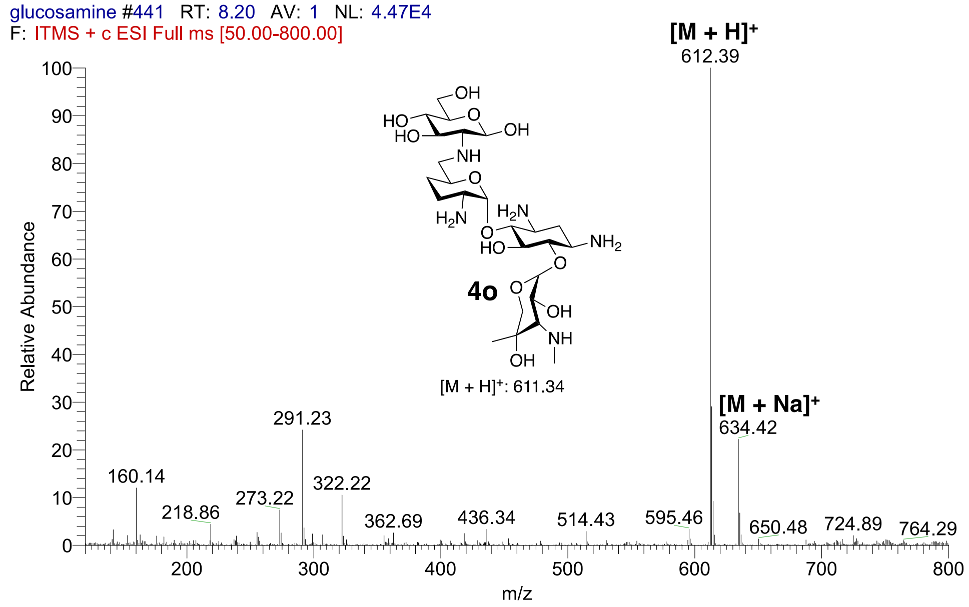

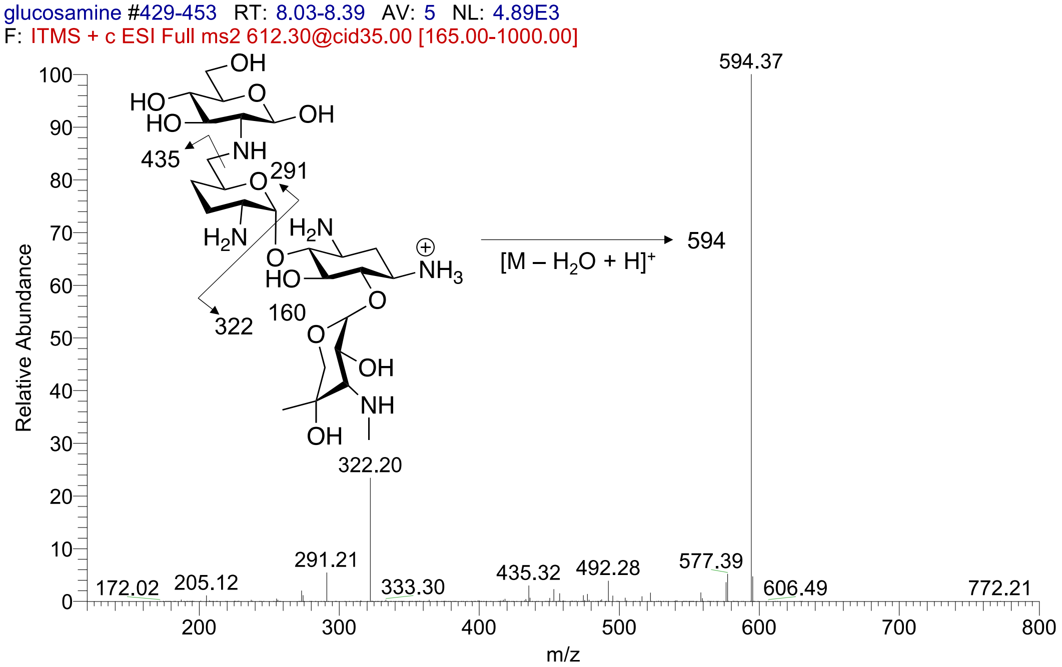

Figure S66: LC-MS analysis of 6'-*N*-(Glucosaminyl)-gentamicin C1a (**4o**).

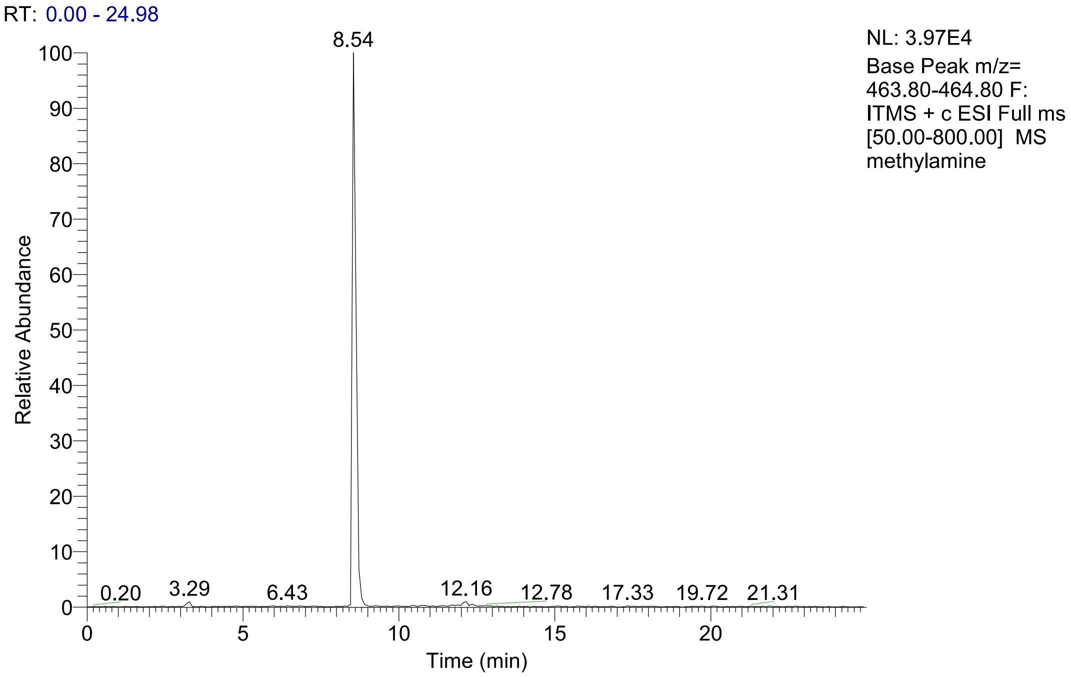

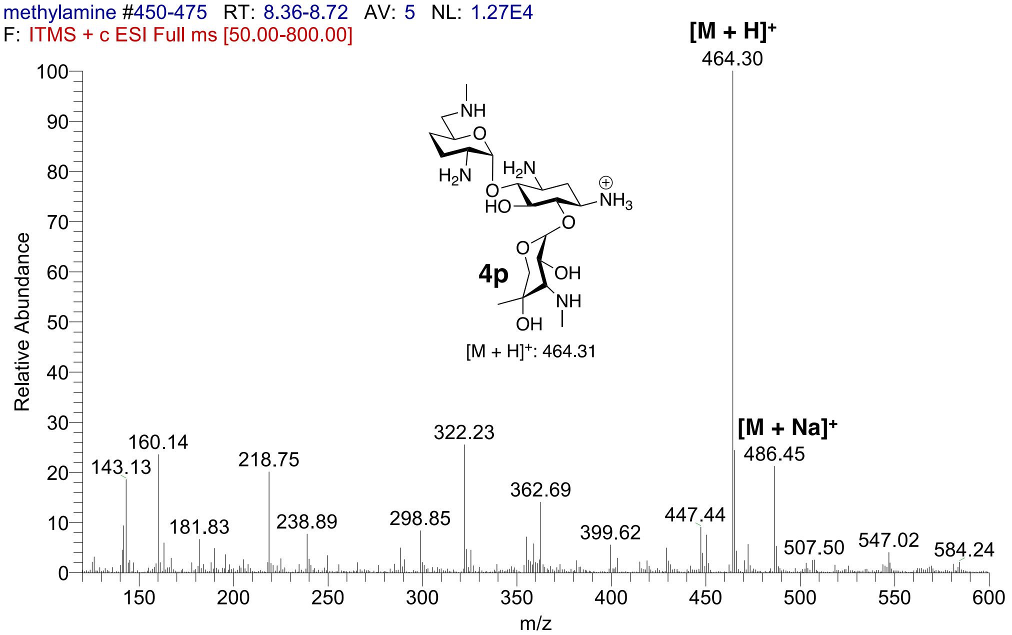

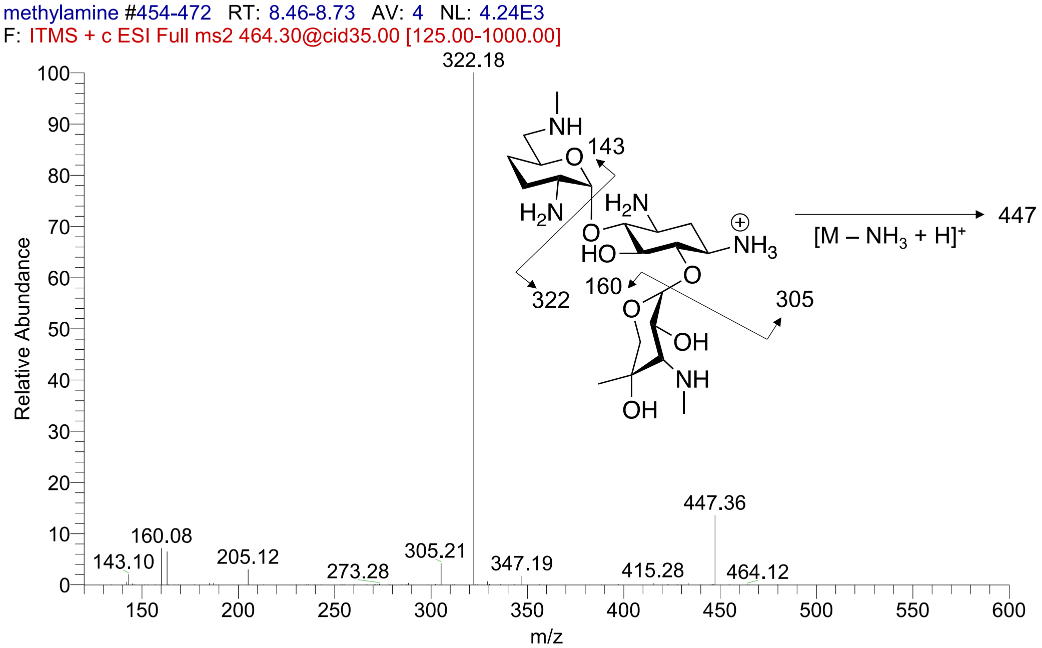

Figure S67: LC-MS analysis of 6’-*N*-(Methyl)-gentamicin C1a (**4p**).

8. References

1 D. Dobrijevic, L. A. Nematollahi, H. C. Hailes and J. M. Ward, *Biotechniques*, 2020, **69**, 384–387.

2 F. Huang, D. Spiteller, N. A. Koorbanally, Y. Li, N. M. Llewellyn and J. B. Spencer, *ChemBioChem*, 2007, **8**, 283–288.

3 X. Chen, H. Zhang, S. Zhou, M. Bi, S. Qi, H. Gao, X. Ni and H. Xia, *Microb. Cell Fact.*, 2020, **19**, 1–12.

4 European Committee for Antimicrobial Susceptibility Testing (EUCAST), *Clin. Microbiol. Infect.*, 2003, **9**, 1–7.

5 H. Katano, Y. Kuroda, S. Taira, C. Maruyama and Y. Hamano, *Anal. Sci.*, 2017, **33**, 499–504.

6 X. Chen, Q. Li, H. He, J. Zhang and Z. Mao, *RSC Adv.*, 2019, **9**, 12174–12181.

7 H. Umezawa and S. Kondo, in *Methods in Enzymology*, 1975, vol. 43, pp. 263–278.

8 I. Nudelman, L. Chen, N. M. Llewellyn, E. H. Sahraoui, M. Cherniavsky, J. B. Spencer and T. Baasov, *Adv. Synth. Catal.*, 2008, **350**, 1682–1688.

9 T. D. Minogue, H. A. Daligault, K. W. Davenport, K. A. Bishop-Lilly, S. M. Broomall, D. C. Bruce, P. S. Chain, O. Chertkov, S. R. Coyne, T. Freitas, K. G. Frey, H. S. Gibbons, J. Jaissle, C. L. Redden, C. N. Rosenzweig and S. L. Johnson, *Genome Announc.*, 2014, **2**, 1–2.

10 K. M. A. Aboshanab, Bergische Wuppertal University, 2005.

11 Y. Ota, H. Tamegai, F. Kudo, H. Kuriki, A. Koike-Takeshita, T. Eguchi and K. Kakinuma, *J. Antibiot.*, 2000, **53**, 1158–1167.

12 A. Hosoyama, M. Hashimoto, K. Tsuchikane, S. Hirakata, A. Uohara, S. Ohji, N. Ichikawa, A. Kimura, A. Yamazoe and N. Fujita, Whole genome shotgun sequence of Paenibacillus chitinolyticus NBRC 15660, https://www.ncbi.nlm.nih.gov/nuccore/BBJT00000000.1/, (accessed 24 March 2022).

13 W. R. Hong, M. Ge, Z. H. Zeng, L. Zhu, M. Y. Luo, L. Shao and D. J. Chen, *Biotechnol. Lett.*, 2009, **31**, 449–455.
